# Lattice dynamics of the tin sulphides SnS_2_, SnS and Sn_2_S_3_: vibrational spectra and thermal transport†
†Electronic supplementary information (ESI) available. See DOI: 10.1039/c7cp01680h



**DOI:** 10.1039/c7cp01680h

**Published:** 2017-05-03

**Authors:** Jonathan M. Skelton, Lee A. Burton, Adam J. Jackson, Fumiyasu Oba, Stephen C. Parker, Aron Walsh

**Affiliations:** a Department of Chemistry , University of Bath , Claverton Down , Bath BA2 7AY , UK . Email: j.m.skelton@bath.ac.uk; b Laboratory for Materials and Structures , Institute of Innovative Research , Tokyo Institute of Technology , 4259 Nagatsuta , Midori-ku , Yokohama 226-8503 , Japan; c Kathleen Lonsdale Materials Chemistry , Department of Chemistry , University College London , 20 Gordon Street , London WC1H 0AJ , UK; d Department of Materials , Imperial College London , Exhibition Road , London SW7 2AZ , UK; e Global E3 Institute and Department of Materials Science and Engineering , Yonsei University , Seoul 120-749 , Korea

## Abstract

We present an in-depth first-principles study of the lattice dynamics of the tin sulphides SnS_2_, *Pnma* and π-cubic SnS and Sn_2_S_3_. An analysis of the harmonic phonon dispersion and vibrational density of states reveals phonon bandgaps between low- and high-frequency modes consisting of Sn and S motion, respectively, and evidences a bond-strength hierarchy in the low-dimensional SnS_2_, *Pnma* SnS and Sn_2_S_3_ crystals. We model and perform a complete characterisation of the infrared and Raman spectra, including temperature-dependent anharmonic linewidths calculated using many-body perturbation theory. We illustrate how vibrational spectroscopy could be used to identify and characterise phase impurities in tin sulphide samples. The spectral linewidths are used to model the thermal transport, and the calculations indicate that the low-dimensional Sn_2_S_3_ has a very low lattice thermal conductivity, potentially giving it superior performance to SnS as a candidate thermoelectric material.

## Abstract 2

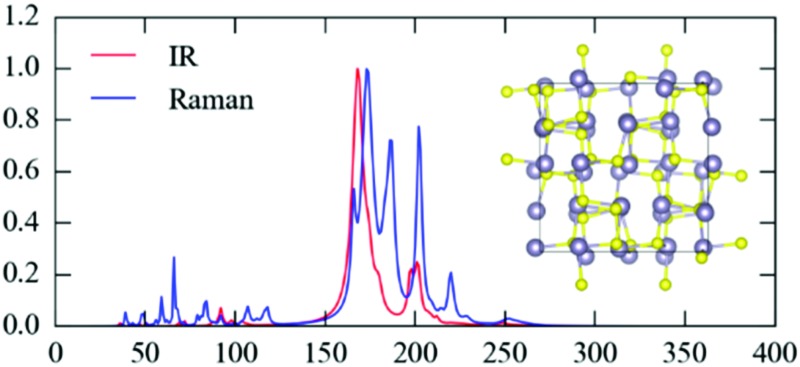
First-principles lattice-dynamics calculations are used to model and compare the vibrational spectra and thermal transport of four bulk tin-sulphide materials.

## 1. Introduction

The drive for “green” energy has spurred a search for new, high-performance materials for photovoltaic (PV) devices for solar-energy conversion, and thermoelectric generators for waste heat recovery. The key challenge in both fields is to find materials with favourable electrical properties that are cheap, earth abundant and non-toxic, and that are easily synthesised and processed.

Due to their simple binary composition and the abundance of tin and sulphur, the tin sulphides Sn_*x*_S_*y*_ have been widely studied as candidate energy materials. SnS has significant potential as a PV absorber, with a large optical absorption coefficient and a bandgap close to the theoretical optimum for peak PV efficiency.^1–3^ It has also been investigated as a thermoelectric material,^4^ in the context of a sustainable replacement for SnSe, which was recently shown to possess record thermoelectric efficiency.^5^

SnS_2_ is of interest due to its layered bonding structure, which is similar to that of 2D transition-metal dichalcogenides such as MoS_2_,^6^ and a wide bandgap that makes it suitable for use as a buffer layer in photovoltaic devices,^7^ as a high surface-area photocatalyst,^8^ and as a photodetector.^9^

Sn_2_S_3_ occurs naturally as the mineral ottemanite,^10,11^ but has been comparatively less intensively studied.^12^ However, preliminary characterisation has indicated that the properties of Sn_2_S_3_ may make it an excellent PV material in its own right.^12^

The rich phase chemistry of the Sn_*x*_S_*y*_ system presents a challenge to synthesis and characterisation.^13^ In addition to the three stable compositions, SnS itself has several known or proposed polymorphs, *viz.* the pseudo-2D herzenbergite *Pnma* structure,^14^ the high-temperatures *Cmcm* phase,^15^ and cubic phases obtained by epitaxial growth^16^ and as nanoparticles.^17–19^ Many early reports identified bulk cubic SnS as a zincblende structure,^17,18,20^ but it has been demonstrated that this phase is highly unlikely to form,^13,21^ and that nanoparticulate cubic SnS most likely corresponds to the recently-identified π-cubic phase with a 64-atom unit cell.^21–23^ Furthermore, Sn_2_S_3_ can be easily misidentified as SnS, due to its similar bandgap and hence colour,^12^ and its similar macroscopic morphology. Indeed, commercial samples of SnS have been reported to be more than 50% Sn_2_S_3_ by mass in the past.^24^

The difficulty in identifying and characterising samples and possible phase impurities may be an important factor in SnS-based PV devices having so far failed to achieve a conversion efficiency above 5%.^25^ Despite near-ideal material properties and a large body of research,^26^ this efficiency falls well short of other current flagship materials such as Cu(In,Ga)Se_2_ (CIGS; >21%),^27,28^ Cu_2_ZnSn(S,Se)_4_ (CZTS/Se; 12%)^29,30^ and hybrid halide perovskites.^31^ The lower mass of S and the stronger chemical bonding in SnS also render it uncompetitive as a thermoelectric in comparison to the benchmark SnSe and nanostructured PbTe.^4,5,32^

It has been shown that the presence of multiple phases in SnS films would likely have a negative impact on photovoltaic (and thermoelectric) performance, with SnS_2_ in particular being predicted to act as a recombination centre for holes and electrons.^33^ Similar issues are of relevance to devices based on CZTS, as tin sulphides are known to occur as secondary phases during CZTS deposition,^34,35^ and again are expected negatively to impact performance.

It is therefore essential to explore techniques for reliably confirming the identity of Sn_*x*_S_*y*_ materials, as well as for identifying tin sulphide impurities in materials such as CZTS.

Recent X-ray photoemission spectroscopy (XPS) measurements showed that, with suitable energy resolution and careful analysis, this technique can reliably distinguish phase-pure *Pnma* SnS and SnS_2_.^12^ However, the presence of both Sn(ii) and Sn(iv) in Sn_2_S_3_ would make it difficult to distinguish from mixtures of SnS and SnS_2_, and hence to unambiguously assign impurity peaks.

X-ray diffraction (XRD) can also indicate the presence of multiple phases. However, the assignment can be ambiguous, and it is not uncommon in the literature to find samples assigned as “predominantly” one phase based on this technique.^36–38^ There are also examples where XRD has conflicted with macroscopic appearance: one study reported the synthesis of yellow plates of Sn_2_S_3_ and black needles of SnS_2_ based on XRD,^39^ which is the opposite expected from theoretical modelling and single-crystal characterisation.^33^

Infrared (IR) and Raman spectroscopy are ubiquitous techniques for materials characterisation, which have proven extremely useful for CZTS among others.^35,40–42^ To use them effectively, however, requires high-quality reference spectra, which can be challenging to obtain for systems with complex phase diagrams. Recent advances in *ab initio* modelling techniques have enabled the accurate prediction of phonon spectra,^43,44^ including IR and Raman intensities and spectral linewidths,^45^ providing a valuable aid to experimental characterisation.

In this work, we present the simulation and complete assignment of the spectra of the four bulk-stable tin sulphide systems,^21^ *viz.* SnS_2_, *Pnma* and π-cubic SnS and Sn_2_S_3_. In the following section, we provide an overview of the lattice-dynamics techniques employed in this work. Section 3a details the four compounds under study, and Section 3b presents their harmonic phonon dispersions and vibrational density-of-states curves. In Section 3c, we present a full set of simulated infrared and Raman spectra, and discuss how the different sulphide phases could be identified and distinguished from their spectral signatures. Finally, in Section 3d we model and compare the lattice thermal conductivity of the four sulphides, and investigate the potential of the as-yet unexplored Sn_2_S_3_ and π-cubic SnS as candidate thermoelectric materials.

## 2. *Ab initio* lattice-dynamics calculations

### a. Harmonic phonon frequencies and eigenvectors

Within the harmonic approximation, the phonon frequencies and eigenvectors of a periodic system can be determined from the second-order interatomic force-constant matrices *Φ*(*jl*,*j*′*l*′), which capture the change in force ***F*** on atom *j* in response to the displacement of atom *j*′ from its equilibrium position ***r***:1
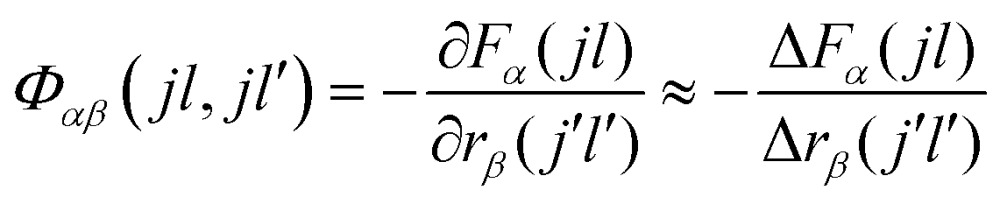
The indices *l* and *l*′ in eqn (1) label the unit cells of the two atoms, and *α* and *β* label the three Cartesian directions *x*, *y* and *z*. As illustrated by the right-hand side expression, a common method for computing the force-constant matrices is to perform small displacements of atoms along symmetry-inequivalent directions and to compute the resultant forces, thereby building up the required derivatives using finite differences.

The force-constant matrices can be transformed to the dynamical matrices ***D***(**q**) for a given phonon wavevector **q**, which captures the wavelength and propagation of the atomic-displacement wave (eqn (2)). Diagonalisation of ***D***(**q**) then yields the set of phonon frequencies (eigenvalues) *ω*(**q**,*s*) and atomic-displacement patterns (eigenvectors) ***W***(**q**,*s*) for the given wavevector.2
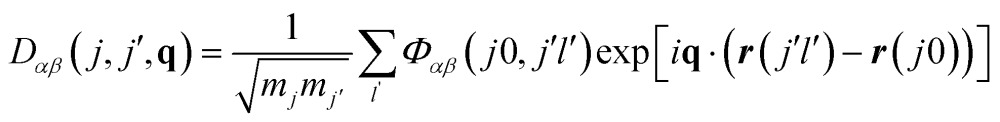
where *m*_*j*_ are the atomic masses. The phonon frequencies *ω* are indexed by the **q**-point and a band index *s* which runs over the 3*n*_a_ modes arising from the *n*_a_ atoms in the primitive cell.

The (real-space) range of the force-constant matrices determines the accuracy with which the dynamical matrices for wavevectors away from the Brillouin zone centre (*Γ*; **q** = (0,0,0)) can be calculated, and so the finite-displacement calculations are often performed on supercell expansions of the primitive unit cell, *e.g.* using the Parlinski–Li–Kawazoe method.^46^ Alternatively, the dynamical matrices for a given set of **q**-vectors can be evaluated directly using perturbation theory, and force-constant matrices to an appropriate range obtained by back transformation.

### b. Infrared and Raman activities

The visible and infrared radiation used in conventional infrared (IR) and Raman spectroscopy only interacts with phonon modes close to the zone centre where the phonon wavelength approaches infinity. IR and Raman spectra are therefore effectively a phonon density of states (DoS) for **q**-vectors close to the *Γ* point, with the spectral lines weighted by the spectroscopic activity of the modes and broadened by the lifetimes.

The eigenvectors obtained directly from the diagonalisation of the dynamical matrix are weighted by the atomic masses. The relation between ***W***(**q**,*s*) and atomic displacements ***u***(**q**,*s*,*jl*) is given by:3
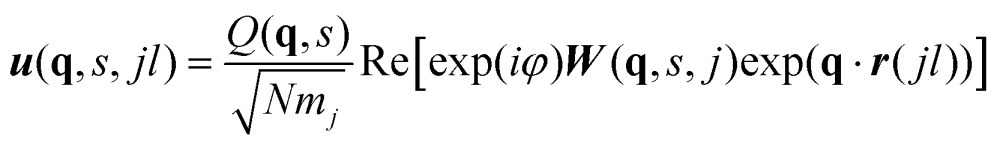
where *Q*(**q**,*s*) is the normal-mode coordinate (amplitude), *N* is the number of unit cells in the supercell used to model the displacement and *φ* is an arbitrary phase factor. Setting *φ* = 0 and considering *Γ*-point modes, for which ***W***(**q**,*s*) are real, *N* = 1 and the second exponent term is zero, simplifies eqn (3) considerably.

To calculate the infrared intensities, we follow ref. 47 and define the *Γ*-point “eigendisplacements” ***X***(*s*,*j*) as the eigenvectors after division by the square root of the atomic masses, such that:4
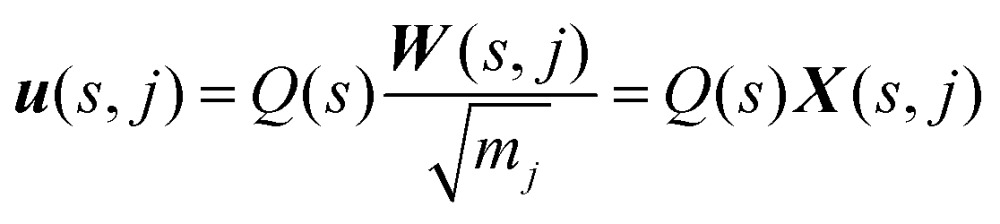
This expression is equivalent to the *Γ*-point simplification of eqn (3); we have also dropped the indexing by **q**, and assume **q** = (0,0,0) in the remainder of this section.

The absolute infrared (IR) activities *I*_IR_(*s*) are given by the square of the change in macroscopic polarisation ***P*** (*i.e.* the dipole moment per unit volume) with respect to displacement along the normal-mode coordinates. The change in polarisation with respect to atomic displacements is captured by the Born effective-charge tensors Z* defined as:5
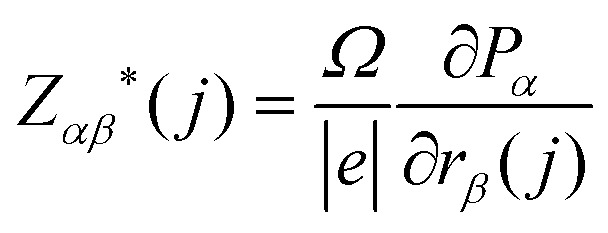
where *Ω* is the volume of the primitive cell. The Born charges can be used to obtain the polarisation derivatives along the mode eigenvectors using the relationship:6
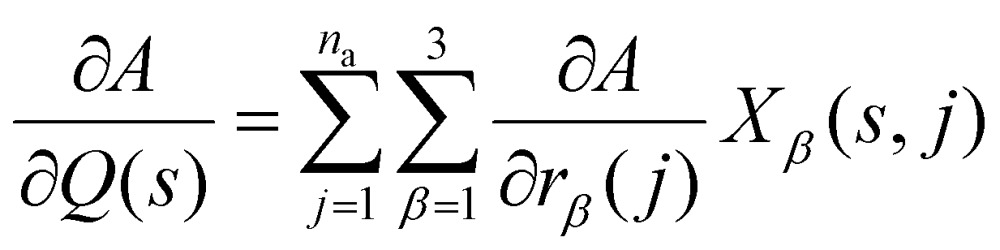
where *A* is a generic physical quantity, and the sum runs over the *n*_a_ atoms in the primitive cell. *I*_IR_ is calculated as:^47,48^
7
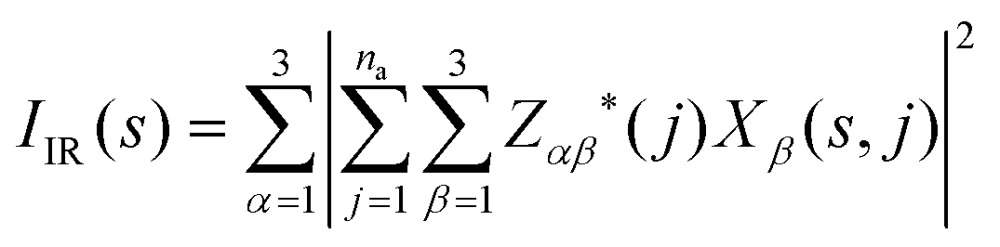
The three components of the dipole derivative in eqn (7) are summed to give the overall intensity.

The Raman activity tensors ***I***_Raman_ are given by the change in the polarisability tensor ***α*** along the mode eigenvectors, which can be recast in terms of the macroscopic high-frequency dielectric constant ***ε*** ^∞^:^49,50^
8
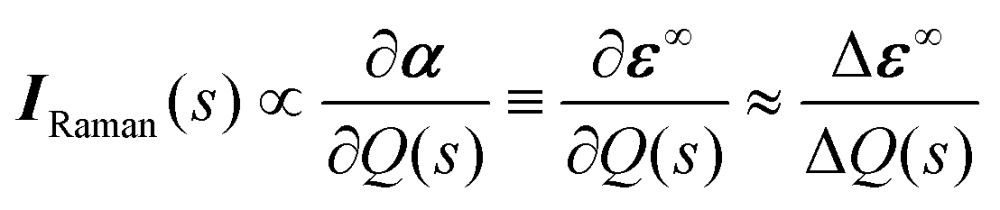
The implementation of the method in ref. 47 used in this work is the central-difference scheme:^50^
9
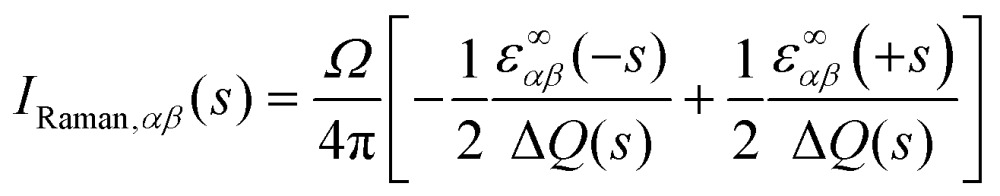
where *ε*∞*αβ*(±*s*) are the components of the dielectric tensor evaluated at positive and negative displacements along the mode *s*.

To obtain the scalar Raman intensities, *I*_Raman_, measured in a typical experiment, the Raman tensor must be averaged for the measurement geometry. This typically corresponds to the direction and polarisation of the incident laser beam, and the direction of the scattered light measured, being perpendicular to one another, which yields the following expression for *I*_Raman_:^47^
10
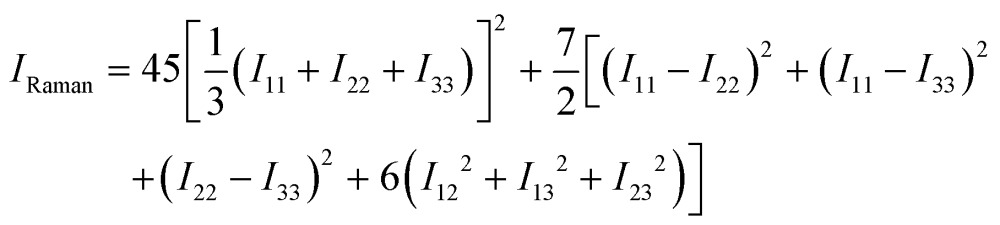
In eqn (10), we have used the notation *I*_*αβ*_ in place of *I*_Raman,*αβ*_(*s*) for brevity.

With *m*_*j*_ in amu, and Z* in *e*, the calculated IR intensities will have units *e* ^2^ amu^–1^. With *Q* in 
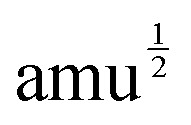
 Å, the elements of the Raman-activity tensor (eqn (9)) have units of 
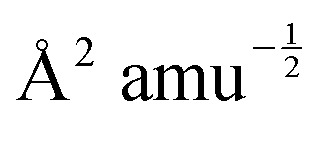
, and the units of the scalar Raman intensity (eqn (10); the square of the Raman activity) are Å^4^ amu^–1^.

### c. Phonon linewidths and thermal conductivity

As discussed in detail in ref. 51, temperature-dependent phonon linewidths *Γ*(*λ*,*T*) for a phonon mode *λ* (with wavevector **q** and band index *s*; we use this single-letter notation in the following for brevity) can be calculated as the imaginary part of the phonon self-energy from many-body perturbation theory.

The many-body equation assumes three-phonon interactions to be the dominant contribution to finite mode lifetimes. The key quantities are the three-phonon interaction strengths *Φ*(*λ*,*λ*′,*λ*′′) between triplets of phonon modes, which can be computed from the harmonic phonon frequencies and eigenvectors and the third-order force-constant matrices *Φ*(*jl*,*j*′*l*′,*j*′′*l*′′) using:^51^
11
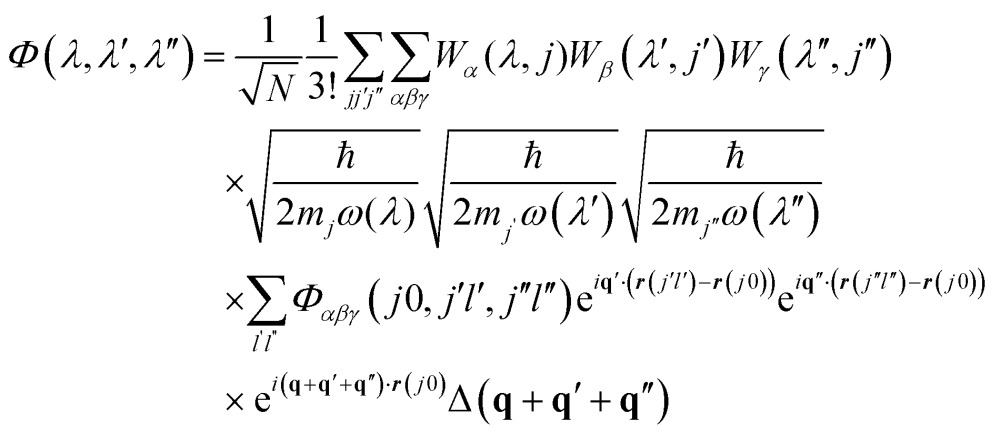



The delta function Δ(**q** + **q**′ + **q**′′) enforces conservation of momentum. *Φ*(*λ*,*λ*′,*λ*′′) are then used to compute the phonon linewidths according to:12
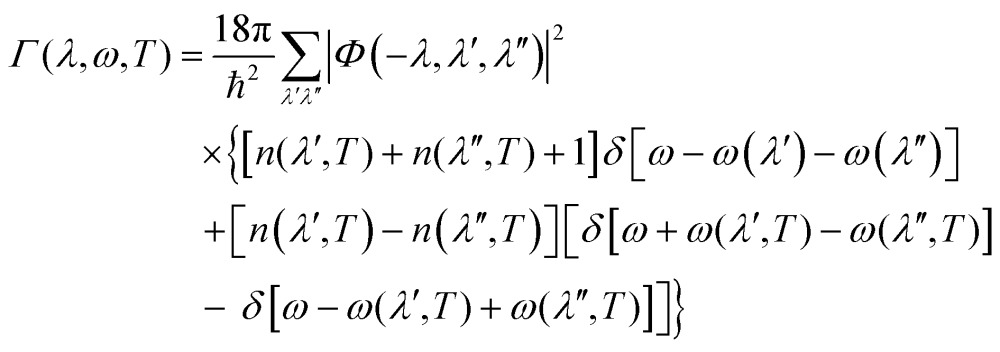
where *n*(*λ*,*T*) are the mode occupation numbers computed from a Bose–Einstein distribution:13
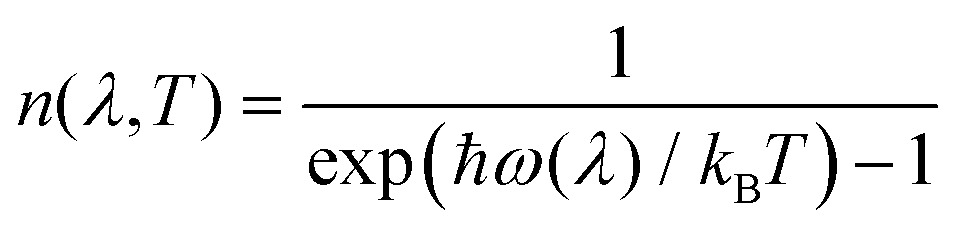



The delta functions in eqn (12) enforce conservation of energy, and the mode linewidths *Γ*(*λ*,*T*) are obtained by setting *ω* = *ω*(*λ*).

The key quantities in eqn (11) and (12) are the phonon frequencies and eigenvectors, which are calculable within the harmonic approximation as described above, and the third-order force constants *Φ*(*jl*,*j*′*l*′,*j*′′*l*′′). These can be obtained in an analogous manner to the second-order force constants, by enumerating the unique pairwise atomic displacements. However, the number of two-atom displacements scales with the supercell size, meaning that the third-order force constants typically must be calculated up to a shorter real-space range than the second-order ones. A longstanding assumption in anharmonic phonon theory is that the range of the third-order interaction is shorter than that of the second-order one, which provides some justification.

Also, it is noteworthy that during the post processing to obtain the phonon lifetimes, the application of eqn (11) scales with the number of phonon bands (and hence the number of atoms in the primitive cell) as well as number of irreducible wavevectors in the Brillouin-zone sampling mesh, and this step can easily require a non-trivial amount of computer time for large and/or low-symmetry primitive cells and dense Brillouin-zone sampling.

The linewidths obtained from the third-order calculations are equivalent to the intrinsic full-width at half-maxima (FWHM) of the peak profiles observed in vibrational-spectroscopic techniques such as IR and Raman. *Γ*(*λ*,*T*) are related to the phonon lifetimes *τ*(*λ*,*T*) according to:^51^
14
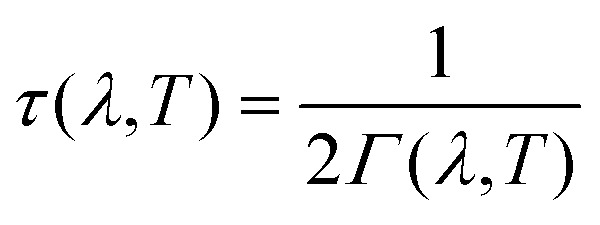
By the Heisenberg uncertainty principle, the uncertainty in energy *E*(*λ*,*T*) = *hν*(*λ*,*T*) is related to *τ*(*λ*,*T*) according to:15
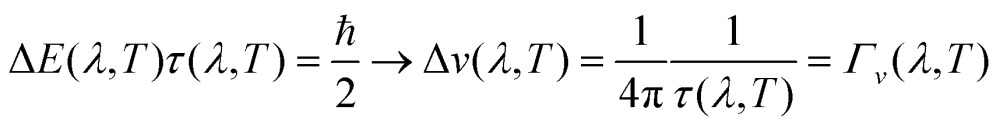
where *Γ*_*v*_ are the linewidths in ordinal frequency units (*i.e. Γ*/2π). Equating *Γ*_*v*_(*λ*,*T*) with the FWHM, IR and Raman spectra can be modelled as a sum of the spectral lines from the *Γ*-point modes, with the lineshapes given by Lorentzian functions with the central frequencies and areas set to the calculated phonon frequencies and IR/Raman activities, respectively:16
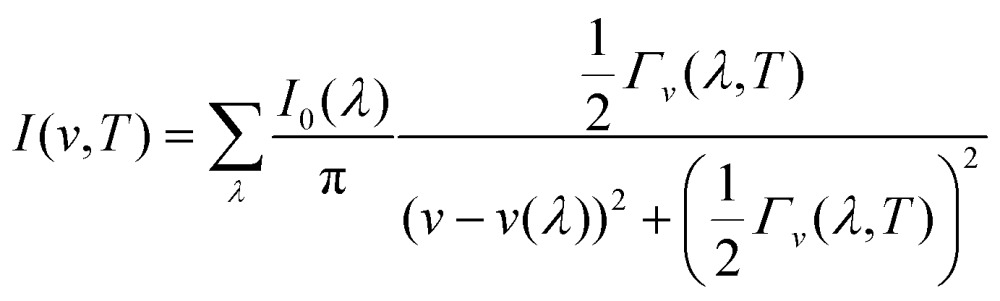
where *I*(*v*,*T*) is the spectroscopic intensity at frequency *v* and temperature *T*. For a given mode, *I*_0_(*λ*) is the height of a delta function at *v* = *v*(*λ*), and the base intensity is spread over a range of frequencies due to the measurement uncertainty arising from the finite lifetime of the mode.

Finally, the mode lifetimes can also be used to calculate the lattice thermal conductivity, ***κ***_latt_, using the single-mode relaxation-time approximation (RTA) solution to the Boltzmann transport equation.^51^ The RTA gives a simple closed-form expression for ***κ***_latt_ in terms of the phonon lifetimes and quantities readily calculable within the harmonic approximation:17
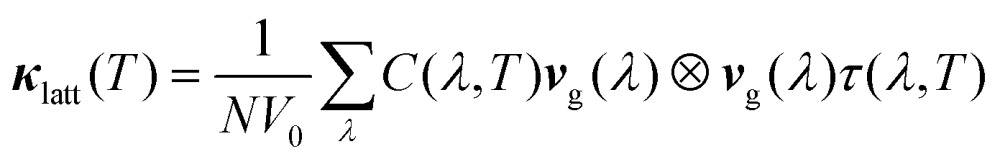
where *N* is again the number of unit cells in the crystal, equivalent to the number of reciprocal-space wavevectors **q** included in the sum over phonon modes, *C*(*λ*,*T*) are the modal heat capacities and ***v***_g_(*λ*) are the mode group velocities. We note that the product ***v***_g_(*λ*)*τ*(*λ*,*T*) gives the phonon mean free path ***Λ***(*λ*,*T*), which appears in the frequently-used alternative form of eqn (17).

## 3. Results and discussion

### a. Optimised crystal structures

**Fig. 1:**
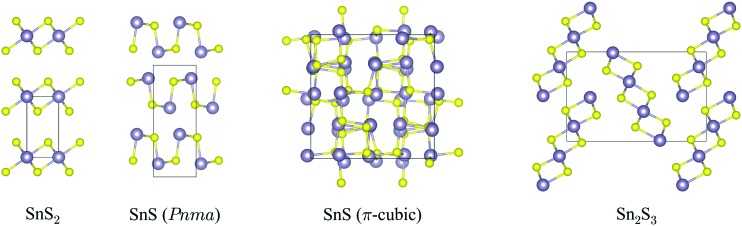
Crystal structures of SnS_2_, *Pnma* and π-cubic SnS and Sn_2_S_3_, viewed along the crystallographic *a* (SnS_2_), *c* (both SnS phases) and *b* axes (Sn_2_S_3_). The Sn and S atoms are coloured grey and yellow, respectively. These snapshots were generated with the VESTA software.^53^

The crystal structures of SnS_2_, *Pnma* and π-cubic SnS and Sn_2_S_3_, optimized at the generalised-gradient approximation (GGA) level of theory using the PBEsol functional,^52^ are shown in Fig. 1, and the corresponding lattice parameters are listed and compared to experimental data in Table 1.

**Table 1:** Optimised lattice parameters of SnS_2_, *Pnma* and π-cubic SnS and Sn_2_S_3_ (DFT/PBEsol). Experimental measurements from ref. 11, 15, 23 and 54 are shown in parentheses. The angles *α*, *β* and *γ* for all four compounds are fixed by symmetry to *α* = *β* = 90° and *γ* = 120° for SnS_2_ and *α* = *β* = *γ* = 90° for both polymorphs of SnS and Sn_2_S_3_

Compound	Space group	*a* [Å]	*b* [Å]	*c* [Å]	*V* [Å^3^]
SnS_2_	*P*3*m*1	3.651 (3.638^54^)	—	6.015 (5.880^54^)	69.42 (69.44^54^)
SnS (*Pnma*)	*Pnma*	4.251 (4.33^15^)	11.082 (11.18^15^)	3.978 (3.98^15^)	187.4 (192.7^15^)
SnS (π-cubic)	*P*2_1_3	11.506 (11.603^23^)	—	—	1523 (1562^23^)
Sn_2_S_3_	*Pnma*	8.811 (8.878^11^)	3.766 (3.751^11^)	13.813 (14.020^11^)	458.4 (458.3^11^)

SnS_2_ contains Sn(iv), which tends to favour octahedral (*e.g.* in SnO_2_) or tetrahedral (*e.g.* in Cu_2_ZnSnS_4_) coordination environments. The compound adopts a hexagonal layered structure in which 2D planes of edge-sharing SnS_6_ octahedra are separated by a van der Waals’ gap.

Both polymorphs of SnS contain Sn(ii), which tends to favour asymmetric coordination environments owing to a stereochemically-active 5s^2^ lone electron pair. The *Pnma* phase of SnS is a “pseudo-2D” orthorhombic structure in which each cation bonds to three S atoms with two slightly different bond lengths (2.633/2.671 Å). The Sn lone pair occupies the fourth coordination site and facilitates weaker bonding along the crystallographic *b* direction. The π-cubic phase (space group *P*2_1_3) contains 64 atoms (32 formula units) in the primitive cell, and can be thought of as a heavily-distorted supercell expansion of a rocksalt (*Fm*3*m*) conventional cell. The local coordination is similar to that in the *Pnma* phase, but in this polymorph the covalent bonding forms a 3D network.

Sn_2_S_3_ is a mixed oxidation state material containing equal proportions of Sn(ii) and Sn(iv). As with *Pnma* SnS, it crystallises in an orthorhombic *Pnma* structure, but consisting of 1D chains of six-coordinate Sn(iv)S_6_ octahedra capped by tetrahedral Sn(ii), with one coordination site occupied by the lone pair as in SnS. The mixed-valence Sn_2_S_3_ can thus be regarded as having a reduced dimensionality compared to the single-valence phases.

### b. Phonon dispersion and density of states curves

**Fig. 2:**
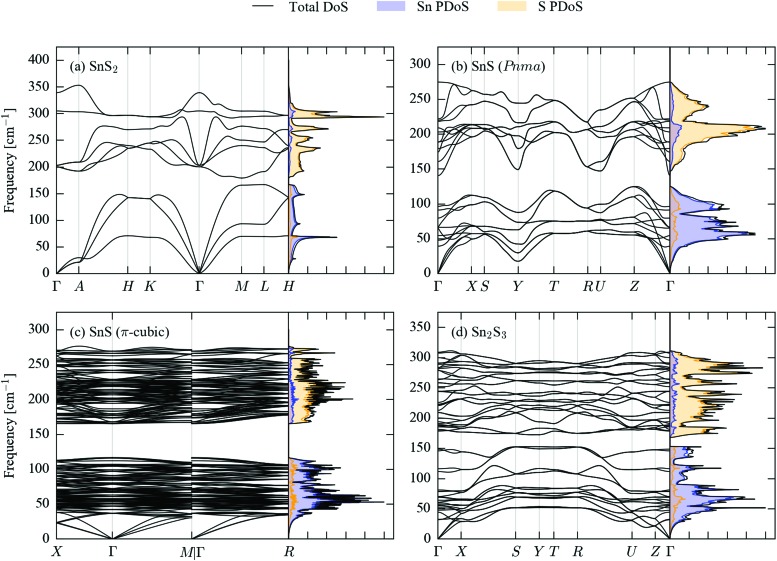
Simulated phonon dispersion and density of states (DoS) curves for SnS_2_ (a), *Pnma* (b) and π-cubic (c) SnS, and Sn_2_S_3_ (d). The partial DoS (PDoS) projected onto Sn and S is overlaid as filled curves with blue and orange shading, respectively.

Fig. 2 shows the calculated phonon dispersion and density of states (DoS) curves for SnS_2_, *Pnma* and π-cubic SnS and Sn_2_S_3_.

The primitive cells of the four structures contain 3, 8, 64 and 20 atoms, respectively, giving rise to 9, 24, 192 and 60 phonon bands at each phonon wavevector (**q**-point). The larger crystal structures therefore have considerably more complex band dispersions and DoS curves with more fine structure. The phonon frequencies in the four compounds span a range of approx. 400 cm^–1^, with the upper limits falling in the order SnS_2_ > Sn_2_S_3_ > SnS; this could be related to the different Sn oxidation states in the three systems, as Sn(iv) would be expected to have a higher charge than Sn(ii), leading to a larger ionic component to the Sn–S bonding.

All four sets of phonon data have in common a separation between the low- and high-frequency modes (a so-called “phonon bandgap”). A projection of the mode eigenvectors onto the Sn and S atoms shows that the lower-frequency branches are primarily motions of the Sn atoms, while the higher-frequency branches are mainly associated with S.

Another common feature shared by the SnS_2_, *Pnma* SnS and Sn_2_S_3_ band structures is a large frequency dispersion along segments of the Brillouin zone corresponding to real-space directions with strong covalent bonding, contrasting with noticeably shallower variation along directions with weak non-bonded interactions. In the dispersion of SnS_2_, the segments *Γ*–*A*, *H*–*K* and *M*–*L* along the band path correspond to the hexagonal *c* axis, and the branches are flat in comparison to the others. A similar contrast can be seen between the *X*–*S* and *R*–*U* segments of the Sn_2_S_3_ dispersion, which correspond to the short crystallographic *b* direction along which the chains are bonded, and the other directions, particularly in the lower-frequency branches. In *Pnma* SnS, which has the same space group, the *b* axis is the layering direction, and the same segments of the band structure thus generally show a shallower dispersion than the others, although the difference is not as striking as in Sn_2_S_3_.

The acoustic modes of π-cubic SnS show a large frequency dispersion, comparable to the equivalent modes in the *Pnma* phase, but the high density of branches above ∼40 cm^–1^ make it difficult to discern whether or not the higher-frequency modes also exhibit large dispersion.

The shallow phonon-frequency dispersion along the notionally weakly-bonded directions in the 2D/1D crystals evidence the bond-strength hierarchy in these materials, and in particular lend some support to considering *Pnma* SnS and Sn_2_S_3_ to be “pseudo 2D/1D”. The different bonding strengths are also reflected to a large extent through an anisotropy in the elastic constants (Tables S1–S4, ESI†). The *C*_33_ elastic constant of SnS_2_ was calculated to be 13.9 GPa, which is ∼9× smaller than the *C*_11_/*C*_22_ elastic constants of 124 GPa and indicates the structure to be much less resistant to compression along the *c* direction. The *C*_11_, *C*_22_ and *C*_33_ elastic constants of Sn_2_S_3_ are 34.0, 93.7 and 44.5, which suggests that the structure is least compressible along the strongly-bonded crystallographic *b* direction, as expected. On the other hand, as with the phonon dispersion the calculated elastic constants of *Pnma* SnS give a less clear picture – while the largest *C*_33_ elastic constant (89.4 GPa) is that associated with the shortest of the three crystallographic axes, the *C*_22_ elastic constant is larger than the *C*_11_ by a significant margin (69.5 *vs.* 44.3 GPa).

### c. Infrared and Raman spectra

The differences in structure and bonding and in the phonon densities of states of the four sulphides suggest that there should be significant differences in the frequencies and spectral intensities of the *Γ*-point modes probed in IR and Raman experiments. We therefore calculated the spectroscopic activities of these modes and used them to generate simulated spectra. As shown in previous work, lifetime-broadening effects can lead to significant differences in the form of measured spectra when compared to simulations assuming a uniform linewidth.^45^ To account for this possibility, we also included phonon linewidths in the simulated spectra, which were calculated using the many-body perturbative approach implemented in the Phono3py software.^51^

**Fig. 3:**
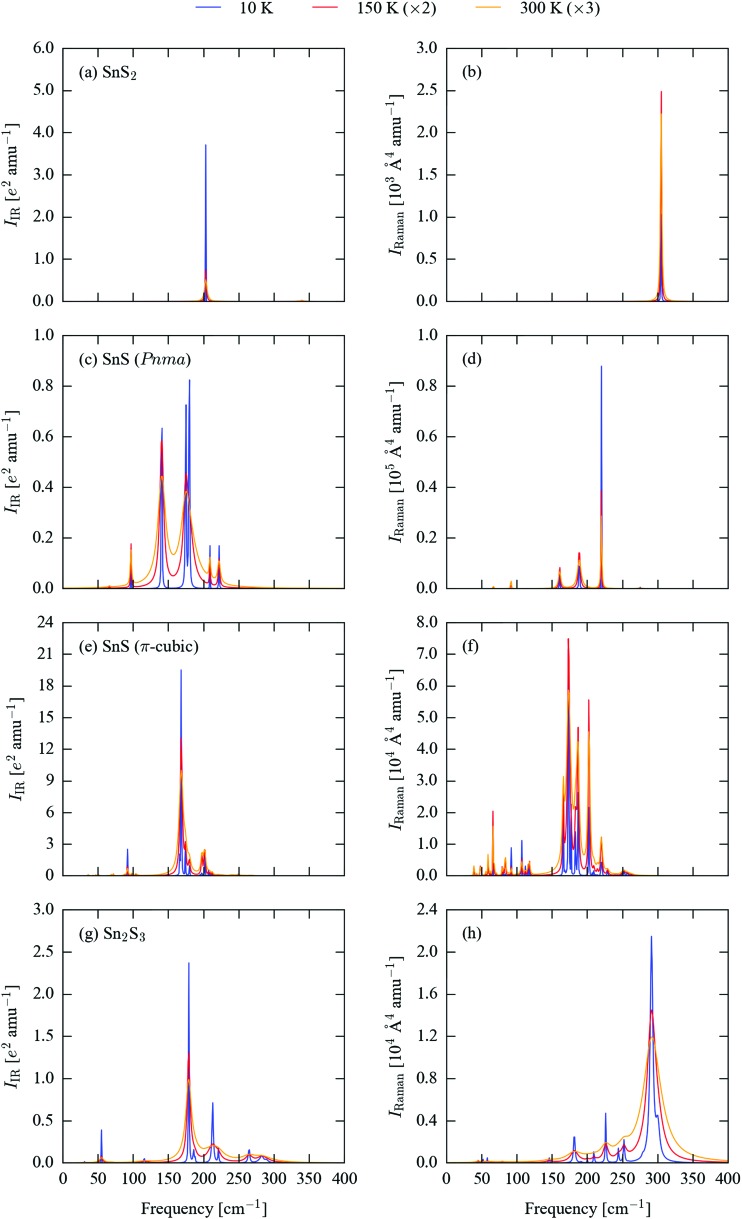
Simulated infrared (IR; a, c, e and g) and Raman (b, d, f and h) spectra of SnS_2_ (a and b), *Pnma* SnS (c and d), π-cubic SnS (e and f) and Sn_2_S_3_ (g and h). The spectral lines have been broadened using calculated 10 K (blue), 150 K (red) and 300 K (orange) linewidths. For clarity, the simulated spectra at the latter two temperatures have been enhanced by 2× and 3×, respectively.

**Fig. 4:**
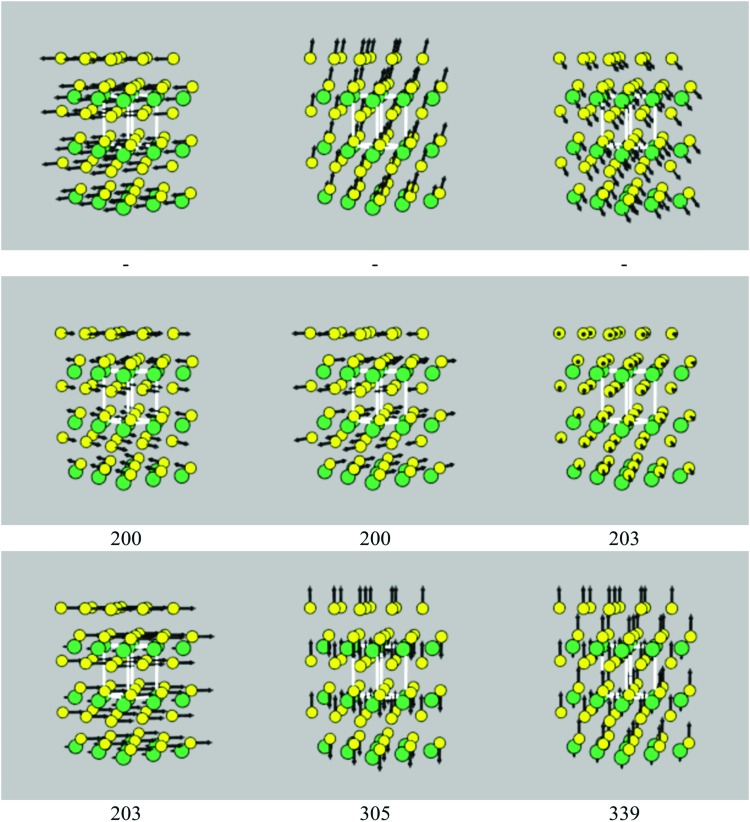
Phonon eigenvectors of the nine *Γ*-point modes of SnS_2_, with frequencies as marked (cm^–1^). The Sn and S atoms are coloured green and yellow, respectively. The three acoustic modes, which correspond to rigid translations of the crystal lattice, necessarily have zero frequency, and so the frequencies of these modes are not shown. These images were generated using the ascii-phonons software.^55^

Spectra broadened with 10 K, 150 K and 300 K linewidths are shown in Fig. 3, and a complete set of peak tables, including assignments of the irreducible representations of the modes, are given in Tables S5–S8 (ESI†). The mode eigenvectors are shown in Fig. 4 and Fig. S1–S3 (ESI†).

Excluding the three acoustic modes, which at *Γ* correspond to rigid translations of the lattice, SnS_2_ has six vibrational modes, with the irreducible representation *Γ* = A_1g_ ⊕ A_2u_ ⊕ E_g_ ⊕ E_u_. The eigenvectors of the nine modes are shown in Fig. 4. The E_u_ modes at 203 cm^–1^, which correspond to planes of Sn and S atoms sliding with respect to one another, are IR active, while the A_1g_ mode at 305 cm^–1^, corresponding to compression of the SnS_2_ layers, is Raman active, resulting in a single main band in each spectrum (Fig. 3a and b). The E_u_ modes are predicted to have a narrow linewidth of 0.03 cm^–1^ at 10 K, resulting in a sharp peak. The linewidth increases to 2.0 and 4.3 cm^–1^ at 150 and 300 K, respectively, and results in a substantial relative reduction in the intensity at the central wavelength. The linewidth of the Raman-active A_1g_ mode undergoes a less marked increase with temperature, with a correspondingly less dramatic change to the peak shape.

*Pnma* SnS has 21 optic modes, which reduce to *Γ* = 4A_g_ ⊕ 2A_u_ ⊕ 4B_1g_ ⊕ B_1u_ ⊕ 2B_2g_ ⊕ 3B_2u_ ⊕ 2B_3g_ ⊕ 3B_3u_. The corresponding mode eigenvectors are illustrated in Fig. S1 (ESI†). The B_1u_ and one each of the B_2u_ and B_3u_ modes are strongly IR active, leading to three sharp peaks between ∼125–200 cm^–1^ in the 10 K IR spectrum (Fig. 3c). At higher temperatures, line broadening causes the closely-spaced peaks at 174 and 180 cm^–1^ to merge into a single broad feature with an apparent intensity similar to the third absorption at 141 cm^–1^. The other two B_3u_ and one of the B_2u_ modes are also weakly IR active and give rise to a set of smaller features at 10 K, which remain visible against the more intense peaks from the other three modes at 150 and 300 K. The A_g_ modes at 189 and 220 cm^–1^ and the B_2g_ mode at 161 cm^–1^ are Raman active. The 189 cm^–1^ mode has the highest base intensity, but the 220 cm^–1^ mode has a narrower linewidth, resulting in the latter forming the most prominent of the three peaks in the spectrum (Fig. 3d). The 161 cm^–1^ mode gives rise to a relatively weak feature at all three temperatures. Several of the other modes show weak Raman activity, most notably an A_g_ mode around 92 cm^–1^; this mode maintains a relatively narrow linewidth up to room temperature, and as such becomes more prominent relative to the three main peaks in the 300 K spectrum.

The high-symmetry space group of π-cubic SnS allows for only three irreducible representations, *viz.* the singly-degenerate A, doubly-degenerate E and triply-degenerate T. Most of the *Γ*-point modes are degenerate, and the 189 optic branches reduce to *Γ* = 16A ⊕ 16E ⊕ 47T. The mode eigenvectors are shown in Fig. S2 (ESI†). The low-temperature IR spectrum (Fig. 3e) is dominated by a strongly IR-active T mode at 168 cm^–1^, which is around an order of magnitude stronger than the other IR-active modes at 92, 166, 174, 180, 197–202 and 249 cm^–1^. Around half of the other T modes are also weakly IR active, but have much lower intensities than the primary absorption bands. The linewidth increases significantly with temperature, such that at 300 K much of the fine structure visible at low temperature is lost. There are three strongly Raman-active A modes at 174, 187 and 202 cm^–1^, two prominent E modes at 166 and 183 cm^–1^, and a collection of weaker modes between ∼50–125 and 200–250 cm^–1^. As for the IR modes, temperature leads to peak broadening, but the majority of the low-temperature features remain discernible at 300 K (Fig. 3f).

The 57 *Γ*-point optic modes of Sn_2_S_3_ reduce to *Γ* = 10A_g_ ⊕ 5A_u_ ⊕ 5B_1g_ ⊕ 9B_1u_ ⊕ 10B_2g_ ⊕ 4B_2u_ ⊕ 5B_3g_ ⊕ 9B_3u_ (eigenvectors shown in Fig. S3, ESI†). As for the other sulphides, however, only a small subset display significant spectroscopic activity. The three prominent features in the 10 K IR spectrum (Fig. 3g) arise from a sharp B_3g_ band at 55 cm^–1^, B_1u_ and B_2u_ bands at ∼179 cm^–1^ and a B_2u_ mode at 213 cm^–1^. There are some notable smaller peaks at 186 and 221 cm^–1^, plus a cluster of weakly IR-active modes from 260–290 cm^–1^. The calculated phonon lifetimes for this system are comparatively very short, leading to substantial line broadening at 150 and 300 K. A similar pattern is observed in the Raman spectrum in Fig. 3h. The A_g_ mode at 291 cm^–1^ has a significantly higher Raman intensity than any of the others, but is characterised by very broad linewidths of 34, 85 and 159 cm^–1^ at 10, 150 and 300 K, respectively. A second A_g_ mode at 300 cm^–1^ has a more moderate intensity, but a narrower linewidth. In combination, the two features result in a broad, asymmetric peak at 150 and 300 K. Several comparatively weaker features at 182, 210, 226, 244 and 252 cm^–1^ are resolved at 10 K and are visible as fine structure against the main feature at higher temperatures.

We note that for all four materials the calculated Raman intensities were found to be relatively insensitive to the choice of the displacement step used to compute the polarisability derivatives (see Section S4 of the ESI†). Although more sophisticated exchange–correlation functionals, *e.g.* hybrids such as PBE0 or HSE06,^56–59^ may predict more accurate electronic structures and hence Raman activities, the large primitive cell and the number of modes in π-cubic SnS and Sn_2_S_3_, together with the sensitivity of the dielectric constant to the electronic **k**-point sampling, make it infeasible to perform such higher-level calculations at present. We therefore make the assumption that, while the absolute calculated Raman intensities may be in error, the relative intensities should nonetheless be comparable.

We also carefully checked the convergence of the calculated spectral linewidths with respect to the **q**-point grid and interpolation technique used to form the integral over three-phonon interactions (Section S5, ESI†). However, given the prohibitive cost of calculating the third-order force constants with larger supercells, we cannot test whether the range of the third-order force constant are fully converged.

The positions of the main peaks in the simulated room-temperature Raman spectrum of *Pnma* SnS at 92, 161, 189 and 220 cm^–1^ are an excellent match for the Raman shifts reported in ref. 18 and 60 (97/95, 160, 191/190 and 216/218 cm^–1^), although the assignment of the symmetries of the 160 and 190 cm^–1^ modes in ref. 18 are at odds with those determined from the calculations. The intensity pattern in the spectrum in ref. 60 differs from the calculated one, although this could be due in part to experimental issues such as instrumental broadening and/or the presence of a fluorescence background. The prominent features in the room-temperature spectra of SnS_2_ and Sn_2_S_3_ occur at ∼305 and 295 cm^–1^, respectively, again in excellent agreement with the values of 312 and 305 cm^–1^ quoted in ref. 18. The simulated 300 K spectra of SnS_2_, *Pnma* SnS and Sn_2_S_3_ are a reasonable match for the data in ref. 39, albeit with differences in the intensity pattern and some apparently missing peaks in the low-frequency part of the simulated Sn_2_S_3_ spectrum below 100 cm^–1^.

The spectroscopy in ref. 23 identifies the major Raman bands of π-cubic SnS to occur at 59, 71, 90, 112, 123, 176, 192, 202 and 224 cm^–1^, the majority of which are present as features in our simulated room-temperature spectrum (∼59, 66, 83, 109, 119, 175, 187, 203 and 221 cm^–1^). As with the spectrum of *Pnma* SnS, there are some notable differences in band intensities between the simulated and experimentally-recorded spectra, particularly at lower frequencies, although this could again be due in part to the measurement setup. The mode symmetries assigned to the bands are not consistent with the irreducible representations of the T point group of *P*2_1_3; based on the calculations, the modes can be tentatively labelled as follows: 59: E, 66/83: A, 109: A/E, 119/175: A, 187: A/E, 203: A/E/T, 221: A.

The simulated spectra in Fig. 3, particularly those of SnS and Sn_2_S_3_ (Fig. 3c–f) illustrate the impact of lifetime-broadening effects in potentially rendering characteristic spectral features difficult to identify, a problem which would in many cases be further exacerbated by instrumental broadening. These simulations therefore suggest that for characterisation, and in particular for detecting small amounts of phase impurities, it would be desirable to record the spectra at as low a temperature as possible, and to optimise instrumental parameters to maximise resolution.

The four sets of simulated IR spectra show markedly different characteristic bands that should allow impurities of some phases to be identified in bulk samples of others. For example, the multiple intense absorptions of *Pnma* SnS should allow this phase to be identified by IR measurements, and the lower-frequency absorptions are sufficiently well separated from the bands in the spectra of the other sulphides that it may be possible to use IR to detect *Pnma* SnS as an impurity in bulk samples of the other materials. On the other hand, the simulated IR spectra of π-cubic SnS and Sn_2_S_3_ are broadly similar in overall form, and would be difficult to tell apart except using a high-resolution instrument and/or a low measurement temperature. The purity of bulk SnS_2_ could be assessed by IR from its single sharp absorption peak at low temperatures, but it may be difficult conclusively to identify a weak SnS_2_ impurity peak in the spectra of the other three sulphides.

Comparing Fig. 3b, d, f and g suggests that Raman would be a very good technique for distinguishing the four materials. The single sharp Raman feature in SnS_2_ is well separated from the peaks in the two SnS phases, which suggests it would be possible to detect S-rich impurities in SnS samples. Similarly, it should be relatively easy to use Raman to check for the presence of Sn-rich phases in bulk SnS_2_. The primary Sn_2_S_3_ Raman band overlaps the SnS_2_ peak, but is fairly well separated from the features from the two SnS phases. The large difference in linewidth between the SnS_2_ and Sn_2_S_3_ spectra should however make it possible to distinguish between SnS_2_ and Sn_2_S_3_ impurities in SnS samples.

Overall, these simulations suggest that both IR and Raman measurements could be a useful tool for characterising tin sulphide samples. In addition, modern Raman setups often operate as microscopes and have the ability to map a substrate, which could be useful, for example, for checking the phase identity of individual particles in a nanoparticle batch, or the homogeneity of SnS films prepared using different techniques.

### d. Thermal-transport properties

The broad spectral linewidths calculated for SnS and Sn_2_S_3_ are indicative of short-lived phonon modes, which would be conducive to a low lattice thermal conductivity.

The performance of thermoelectric materials is typically quantified using the figure of merit *ZT*, defined by:18
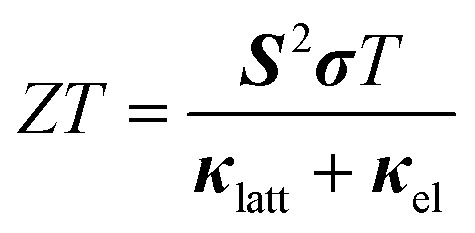
where ***S*** is the Seebeck coefficient, ***σ*** is the electrical conductivity, and ***κ***_latt_ and ***κ***_el_ are the lattice and electronic contributions to the thermal conductivity, respectively. All four quantities, and hence the *ZT* score itself, are implicitly temperature dependent. ***κ***_el_ is usually negligible for semiconductors, and so thermoelectric research typically focuses on systems with large power factors ***S*** ^2^ ***σ*** and low ***κ***_latt_. SnS and Sn_2_S_3_ are both known to be good semiconductors,^12^ and with low lattice thermal conductivities would potentially make good candidate thermoelectrics. The thermal-transport properties of SnS are of particular interest due to the recently-demonstrated very low bulk thermal conductivity of the selenide analogue, *Pnma* SnSe,^5^ which was shown to arise from its strongly-anharmonic lattice dynamics.^61,62^

**Fig. 5:**
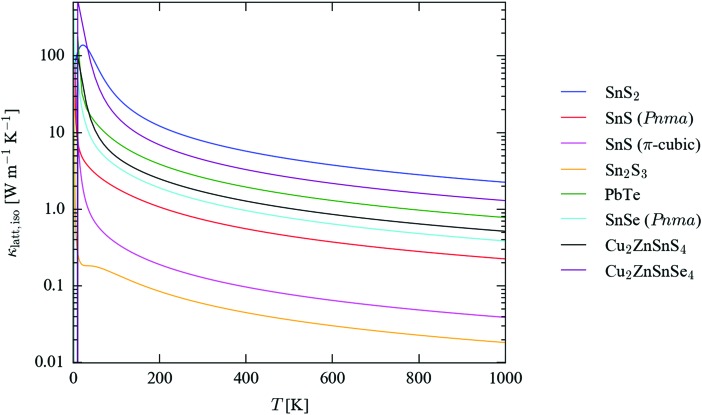
Isotropically-averaged lattice thermal conductivity (*κ*_latt,iso_) as a function of temperature for SnS_2_ (blue), *Pnma* (red) and π-cubic SnS (magenta) and Sn_2_S_3_ (orange), compared to similar calculations on PbTe (green),^43^ *Pnma* SnSe (cyan)^62^ and kesterite Cu_2_ZnSnS_4_ (CZTS; black) and the selenide analogue Cu_2_ZnSnSe_4_ (CZTSe; purple).^45^

Fig. 5 compares the calculated isotropically-averaged lattice thermal conductivities (*κ*_iso_) of SnS_2_, *Pnma* and π-cubic SnS and Sn_2_S_3_ to those of four established and candidate thermoelectric materials, *viz.* PbTe,^43^ *Pnma* SnSe^62^ and the quaternary chalcogenides Cu_2_ZnSnS_4_ and Cu_2_ZnSnSe_4_ (CZTS/Se).^45^ The temperature dependence of the thermal conductivity along each Cartesian direction is plotted separately in Fig. S8–S10 (ESI†). The calculated room-temperature (300 K) thermal conductivities are collected in Table 2, which lists the three diagonal components of the tensors together with the isotropic average, *κ*_iso_ = (*κ*_*xx*_ + *κ*_*yy*_ + *κ*_*zz*_)/3. We also calculate an “anisotropy” value as the ratio of the largest and smallest conductivities along the three directions. For comparison, we show the isotropic thermal conductivities calculated including isotope-scattering effects, which are generally not included in the relaxation-time approximation, and which were not accounted for in the previous calculations in ref. 45 and 62. Comparisons of the full *κ*_iso_(*T*) curves computed with and without isotope effects for each system, together with the axial components of the 300 K tensors, are given in Fig. S11–S17 and Table S13 (ESI†).

**Table 2:** Calculated 300 K lattice thermal conductivities (***κ***_latt_) of SnS_2_, *Pnma* and π-cubic SnS and Sn_2_S_3_, compared to values for PbTe, *Pnma* SnSe, and kesterite Cu_2_ZnSnS_4_ and Cu_2_ZnSnSe_4_ (CZTS/Se) from other calculations.^43,45,62^ Each row lists the thermal conductivities along each Cartesian direction together with the isotropic average *κ*_iso_. Values of *κ*_iso_ calculated including isotope-scattering effects, assuming the natural atomic-mass variances for the constituent elements, are also given where possible, and experimental values are listed for comparison where available.^5,63–66^ The final column presents “anisotropy” values for each system, defined here as the ratio of the maximum and minimum diagonal components of the ***κ***_latt_ tensors

Compound	*κ*_latt,300K_ [W m^–1^ K^–1^]						Anisotropy
	*κ*_*xx*_	*κ*_*yy*_	*κ*_*zz*_	*κ*_iso_	*κ*_iso,isotope_	Expt.	
SnS_2_	11.40	11.40	0.48	7.76	7.60	—	23.99
SnS (*Pnma*)	0.74	0.36	1.10	0.73	0.72	1.25^4^ ^*a*^	3.02
SnS (π-cubic)	—	—	—	0.13	0.13	—	—
Sn_2_S_3_	0.03	0.14	0.01	0.06	0.06	—	10.29
PbTe	—	—	—	2.59^43^ ^*b*^	—	1.99, 2.2^63,64^	—
SnSe (*Pnma*)	1.44	0.53	1.88	1.28^62^	1.24	0.64 (*κ*_*xx*_/*κ*_*zz*_: 0.70, *κ*_*yy*_: 0.45)^5^	3.57
Cu_2_ZnSnS_4_	1.75	1.75	1.57	1.69^45^	1.60	2.95, 4.7^65,66^ ^*c*^	1.12
Cu_2_ZnSnSe_4_	4.68	4.68	3.98	4.44^45^	4.36	3.75^66^ ^*c*^	1.18

^*a*^The thermal-conductivity measurements in ref. 4 are given as *κ*_tot_ = *κ*_latt_ + *κ*_el_.

^*b*^For consistency with the other calculations, we report the 300 K value from the *κ*_latt,iso_(*T*) curve computed at the 0 K lattice volume, as shown in Fig. 5.

^*c*^The CZTS/Se lattice thermal conductivities reported in ref. 66 were measured slightly above 300 K.

The isotropically-averaged thermal conductivity of the four sulphides falls into the range SnS_2_ > *Pnma* SnS > π-cubic SnS > Sn_2_S_3_. Strikingly, π-cubic SnS and Sn_2_S_3_ are both predicted to have a very low *κ*_latt_, some order of magnitude lower than that of all the other compounds, including the benchmark thermoelectrics PbTe and SnSe. Above 100 K, SnS_2_ has the highest *κ*_latt_ of the seven systems included in the comparison, although the conductivity is highly anisotropic, being much smaller along the layered *c* direction than along the two in-plane directions. The calculated *κ*_latt_ of the two SnS phases falls between those of Sn_2_S_3_ and *Pnma* SnSe.

The calculations on *Pnma* SnS predict it to have a lower thermal conductivity than the structurally-analogous selenide, which is unexpected, and indeed with reference to experimental measurements there appears to be a larger error in the calculated isotropic 300 K conductivity of SnSe than in that calculated for SnS. The difference in thermal conductivity is largest between the *κ*_*xx*_ components of the ***κ***_latt_ tensors (corresponding to transport along the crystallographic *a* direction), but the calculations predict the thermal transport to be larger along all three axes of the selenide compared to the sulphide. This is particularly odd given that the chemical bonding in the selenide would be expected to be weaker. CZTS is also predicted in calculations to show a lower thermal conductivity than CZTSe,^45^ whereas the experimental measurements in ref. 66 suggest the opposite.

One possible explanation for this discrepancy is the omission of isotope effects from these calculations: in principle, the natural mass variation among atoms of the same species has an effect on ***κ***_latt_, but it is generally thought that neglecting this compensates for other deficiencies in the relaxation-time approximation, thereby giving overall good agreement with experiment.^43^ A comparison of the *κ*_iso_ values in Table 2 with and without isotope-scattering effects included shows that this alone cannot explain the higher predicted thermal conductivity of the sulphides than the selenides. For both SnS/Se and CZTS/Se, isotope effects reduce *κ*_iso_, but the effect is not large, and is insufficient to lead to a reordering.

Other possible explanations could be the neglect of quasi-harmonic effects (*i.e.* thermal expansion) and/or higher-order anharmonic effects such as four-phonon processes. Phonon frequencies and thermal conductivity have been shown to be strongly volume dependent,^43,44^ and it is possible that the selenides, with “softer” chemical bonding, would have a larger thermal-expansion coefficient than the sulphides, leading to a more significant decrease in the thermal conductivity at higher temperatures. Thermal expansion can be modelled using the quasi-harmonic approximation, but this is beyond the scope of the present study.

Yet another possibility is that calculations and experimental measurements are often simply not directly comparable. Although it is possible to carry out measurements on large single crystals,^5^ it is more typical to prepare *e.g.* pressed powders,^66^ which will naturally have a high concentration of defects such as grain boundaries that are not easily accounted for in calculations on bulk materials.

It is worth noting, however, that despite these caveats the calculated 300 K thermal conductivities are generally within a factor of 2–3 of the experimental measurements, and we would therefore be surprised if the predicted very low thermal conductivities of π-cubic SnS and Sn_2_S_3_ compared to the other compounds were to be qualitatively incorrect.

Previous theoretical studies^62,67^ have reported an axial anisotropy in the thermal conductivity of *Pnma* SnSe, which was ascribed to the different strengths of the bonding interactions along the three crystallographic directions. These calculations indicate that *Pnma* SnS likewise shows a particularly low thermal-conductivity along the non-bonded crystallographic *b* axis, with one of the two perpendicular directions representing an “easy axis” for transport. This anisotropy in thermal-transport properties mirrors that in its electrical properties.^68^ The weak interlayer interactions along the *c* axis of SnS_2_ lead to poor thermal transport in that direction compared to the in-plane conductivity. By analogy, the very low ***κ***_latt_ of Sn_2_S_3_ compared to SnS could be ascribed to its reduced dimensionality, and indeed the thermal conductivity is an order of magnitude larger along the crystallographic *b* axis, corresponding to the direction of the bonding along the chains; however, at 0.14 W m^–1^ K^–1^ at 300 K, this is still close to an order of magnitude smaller than the conductivity along the “easy” direction in SnS at the same temperature (1.10 W m^–1^ K^–1^), implying that intrinsic anharmonicity, as well as low dimensionality, is responsible for its poor thermal transport.

The high thermal conductivity of SnS_2_ is likely to make it a poor candidate thermoelectric in comparison to existing materials. The thermoelectric performance of SnS has previously been investigated,^4^ but its unfavourable electrical properties led to an averaged *ZT* score of 0.16, which is not competitive with either of PbTe or SnSe.^5,69^ The π-cubic phase of SnS has an unusual band dispersion with shallow valence and conduction bands,^70^ which would in principle allow for the formation of multiple “carrier pockets” on doping,^71^ and in conjunction with its low thermal conductivity this may make it a good candidate thermoelectric. Sn_2_S_3_ has been shown to have good electrical properties^12^ and is predicted to have ambipolar dopability,^72^ which, together with the very low ***κ***_latt_ predicted in the present calculations, suggests it too may be a good candidate thermoelectric. If the intrinsic electrical properties of either or both of π-cubic SnS or Sn_2_S_3_ are suitable, or can be made so by doping,^4,71^ taking into account the relative abundance of Sn and S our calculations indicate that the thermoelectric properties of this system may be well worth pursuing further.^73^

## 4. Conclusions

We have presented a complete characterisation of the lattice dynamics of four bulk-stable phases of tin sulphide, *viz.* SnS_2_, *Pnma* and π-cubic SnS and Sn_2_S_3_.

Analysis of the phonon dispersion and DoS curves confirms that all four phases are dynamically-stable energy minima. The complex structures of SnS and Sn_2_S_3_ lead to intricate band dispersions, and the upper limit on the range of phonon frequencies observed in the three systems is relatable to the presence of distinct Sn(ii) and Sn(iv) species. The shallow dispersions along the crystallographic directions associated with weak interactions indicate that the lattice dynamics, and hence the thermal transport, are heavily influenced by the strength of the chemical bonding, and thus intimately linked to the dimensionality of the bonding network in the crystal structures.

From a detailed analysis of the simulated infrared and Raman spectra, we have identified and assigned the key spectral signatures of each phase. Our calculations suggest that both spectroscopic techniques could serve as valuable tools for characterising tin sulphide samples and for identifying phase impurities in bulk materials or thin films. Due to lifetime broadening at room temperature, which is particularly pronounced in the spectra of the two SnS phases and Sn_2_S_3_, low-temperature measurements are expected to give the best feature resolution. The good agreement between the calculated spectra and available experimental data is highly encouraging, and suggests that first-principles simulations such as these can serve as a valuable support to interpreting and assigning vibrational spectra.

Modelling of the thermal-transport properties predict π-cubic SnS and Sn_2_S_3_ to have an unexpectedly-low lattice thermal conductivity. The poor thermal transport of Sn_2_S_3_ is due in part to the 1D covalent bonding, but also to strong intrinsic anharmonicity leading to short phonon lifetimes. With reference to work on SnS, and given its favourable electrical properties, Sn_2_S_3_ is expected to have considerable potential as a high-performance thermoelectric material.

Finally, this study illustrates the utility of lattice-dynamics methods both for general materials modelling and as a tool to inform experimental characterisation. We expect these techniques to be applicable to a broad range of other systems and problems, particularly as the infrastructure and computational power required to tackle systems with complex phase spaces becomes more widely available.

## 5. Methods

All calculations were performed using the plane-wave pseudopotential density-functional theory formalism, as implemented in the Vienna *ab initio* Simulation Package (VASP) code.^74^ The PBEsol exchange–correlation functional^52^ was used in conjunction with projector-augmented wave (PAW) pseudopotentials^75,76^ treating the S 3s and 3p and the Sn 5s, 5p and 4d electrons as valence.

The starting points for the calculations were the primitive unit cells of SnS_2_ (space group *P*3*m*1), SnS (*Pnma*, *P*2_1_3) and Sn_2_S_3_ (*Pnma*),^11,14,23,54^ which were stress relaxed until the magnitude of the forces on the ions fell below 10^–2^ eV Å^–1^.

The elastic constants of the four optimised structures were calculated using the finite-displacement method outlined in ref. 77, with a step size of 0.01 Å.

Harmonic phonon calculations were performed using the Phonopy code,^78,79^ with VASP as the force calculator and a finite-displacement step of 0.01 Å. Force-constant matrices for SnS_2_, *Pnma* and π-cubic SnS and Sn_2_S_3_ were computed using 6 × 6 × 2, 6 × 1 × 6, 2 × 2 × 2 and 2 × 4 × 2 expansions of the primitive unit cells, containing 216, 288, 512 and 320 atoms, respectively.

During post processing, symmetrisation of the force constants was performed within Phonopy, and DoS curves were constructed by evaluating the phonon frequencies on a uniform *Γ*-centred **q**-point mesh with 48 × 48 × 48 subdivisions and interpolating using the linear tetrahedron method. Phonon dispersions were obtained by computing the frequencies along band paths passing through all the high-symmetry points in the *Pnma*, *P*2_1_3 and *P*3*m*1 Brillouin zones.

We also computed the vibrational frequencies and mode eigenvectors at the zone centre (*Γ* point) using the DFPT routines implemented VASP, and found very good agreement between the calculated frequencies and those obtained using Phonopy (see Tables S5–S8 in the ESI†).

The IR intensities of the *Γ*-point modes were calculated using a custom-written script implementing the formula in ref. 47 and 80, while the Raman activities were obtained using the vasp_raman script.^50^ The Born charges and dielectric constants required in these calculations were both computed using the density-functional perturbation theory (DFPT) routines in VASP.^81^

The Phono3py code^51^ was used to set up and post process the linewidth/thermal-conductivity calculations. The third-order force constants were computed using 3 × 3 × 2, 3 × 1 × 3 and 1 × 3 × 1 supercell expansions for SnS_2_, *Pnma* SnS and Sn_2_S_3_, containing 54, 72 and 60 atoms, respectively. Due to its size, the third-order force constants for the π-cubic phase were computed in a single primitive cell (64 atoms). During post processing, the phonon lifetimes of the four systems were sampled on regular *Γ*-centred **q**-point grids with 24 × 24 × 24 (SnS_2_), 16 × 16 × 16 (*Pnma* SnS), 8 × 8 × 8 (π-cubic SnS) and 12 × 12 × 12 (Sn_2_S_3_) subdivisions. Tests of the convergence of the *Γ*-point phonon linewidths and lattice thermal conductivity with respect to mesh sampling are shown in Fig. S4–S7 and S18–S21 (ESI†), respectively.

During all calculations, the kinetic-energy cutoffs for the plane-wave basis sets were set to 500 eV for *Pnma* SnS and 550 eV for SnS_2_, π-cubic SnS and Sn_2_S_3_. The Brillouin zone of SnS was sampled using a regular Monkhorst–Pack (MP) **k**-point mesh^82^ with 8 × 4 × 8 subdivisions, while 8 × 8 × 6, 2 × 2 × 2 and 4 × 8 × 3 *Γ*-centred MP meshes were used for SnS_2_, π-cubic SnS and Sn_2_S_3_, respectively. A tolerance of 10^–8^ eV was applied during the electronic minimisations. The PAW projections were performed in reciprocal space, and the precision of the fast Fourier-transform (FFT) grids was chosen automatically so as to avoid aliasing errors. Finally, for the supercell force and DFPT phonon calculations, an additional FFT grid with 8× the number of points was used to evaluate the PAW augmentation charges, in order to obtain accurate forces.

## 6. Data-access statement

Key raw data from these calculations, including the optimised structures, data from the lattice-dynamics calculations, the simulated spectra and the thermal-conductivity tensors, are available free of charge online from https://doi.org/10.15125/BATH-00357. All other data may be obtained from the authors on request.
