# Supplementary material for: Lattice dynamics of the tin sulphides SnS2, SnS and Sn2S3: vibrational spectra and thermal transport
Source: Phys Chem Chem Phys. 2017 May 3;19(19):12452–65. doi: 10.1039/c7cp01680h (PMC5450010; doi:10.1039/c7cp01680h)
Supplement: Supplementary file 1 [file CP-019-C7CP01680H-s001.pdf]

Electronic Supplementary Material (ESI) for Physical Chemistry Chemical Physics.
This journal is © the Owner Societies 2017

Lattice dynamics of the tin sulphides SnS_2, SnS and Sn_2S_3: vibrational spectra and thermal transport

Electronic supporting information

Jonathan M. Skelton,[1]* Lee A. Burton,[2] Adam J. Jackson,[3] Fumiyasu Oba,[2] Stephen C. Parker[1] and Aron Walsh[1,4,5]

[1] *Department of Chemistry, University of Bath, Claverton Down, Bath BA2 7AY, UK*

[2] *Laboratory for Materials and Structures, Institute of Innovative Research, Tokyo Institute of Technology, 4259 Nagatsuta, Midori-ku, Yokohama 226-8503, Japan*

[3] *Kathleen Lonsdale Materials Chemistry, Department of Chemistry, University College London, 20 Gordon Street, London WC1H 0AJ, UK*

[4] *Department of Materials, Imperial College London, Exhibition Road, London SW7 2AZ, UK*

[4] *Global E^3 Institute and Department of Materials Science and Engineering, Yonsei University, Seoul 120-749, Korea*

* E-mail: j.m.skelton@bath.ac.uk

1. Elastic constants of SnS_2, *Pnma* and π-cubic SnS and Sn_2S_3

[GPa]	1	2	3	4	5	6
1	123.78	31.64	6.98	0.00	-0.32	0.00
2	31.64	123.78	6.98	0.00	0.32	0.00
3	6.98	6.98	13.85	0.00	0.00	0.00
4	0.00	0.00	0.00	46.07	0.00	-0.32
5	-0.32	0.32	0.00	0.00	6.15	0.00
6	0.00	0.00	0.00	-0.32	0.00	6.15

Table S1 Calculated elastic constants C_{ij} of SnS_2.

[GPa]	1	2	3	4	5	6
1	44.35	23.52	38.04	0.00	0.00	0.00
2	23.52	69.45	19.65	0.00	0.00	0.00
3	38.04	19.65	89.40	0.00	0.00	0.00
4	0.00	0.00	0.00	25.64	0.00	0.00
5	0.00	0.00	0.00	0.00	26.31	0.00
6	0.00	0.00	0.00	0.00	0.00	44.43

Table S2 Calculated elastic constants C_{ij} of *Pnma* SnS.

[GPa]	1	2	3	4	5	6
1	68.53	14.98	14.98	0.00	0.00	0.00
2	14.98	68.53	14.98	0.00	0.00	0.00
3	14.98	14.98	68.53	0.00	0.00	0.00
4	0.00	0.00	0.00	15.25	0.00	0.00
5	0.00	0.00	0.00	0.00	15.25	0.00
6	0.00	0.00	0.00	0.00	0.00	15.25

Table S3 Calculated elastic constants C_{ij} of π-cubic SnS.

[GPa]	1	2	3	4	5	6
1	33.98	25.18	17.77	0.00	0.00	0.00
2	25.18	93.66	24.57	0.00	0.00	0.00
3	17.77	24.57	44.53	0.00	0.00	0.00
4	0.00	0.00	0.00	32.91	0.00	0.00
5	0.00	0.00	0.00	0.00	19.31	0.00
6	0.00	0.00	0.00	0.00	0.00	17.11

Table S4 Calculated elastic constants C_{ij} of Sn_2S_3.

2. Peak tables for SnS_2, *Pnma* and π-cubic SnS and Sn_2S_3

Tables S5-S8 present a list of the calculated Γ-point phonon frequencies of SnS_2, *Pnma* and π-cubic SnS and Sn_2S_3 together with the irreducible representations of the mode eigenvectors, the calculated infrared (IR) and Raman activities, and the calculated linewidths at 10, 150 and 300 K. This is the data used to model the spectra in Fig. 3 in the text. The frequencies obtained from both the density-functional perturbation theory (DFPT) routines in the VASP code[1] and using the Phonopy code[2, 3] are shown for comparison (υ_{DFPT} and υ_{FD}, respectively), and typically agree to within 1 cm^{-1}. When determining the irreducible representations, doubly- and triply-degenerate modes are identified based on their frequencies being identical, and for degenerate modes the spectroscopic intensities and linewidths listed are the average of the modes in the set.

υ_{DFPT} [cm^{-1}]	υ_{FD} [cm^{-1}]	Ir. Rep.	I_{IR} [e^2 amu^{-1}]	I_{Raman} [10^3 Å^4 amu^{-1}]	$\Gamma_{10\mathrm{K}}$ [cm^{-1}]	$\Gamma_{150\mathrm{K}}$ [cm^{-1}]	$\Gamma_{300\mathrm{K}}$ [cm^{-1}]
200.26	200.45	E_g	0.00	0.01	0.24	1.06	2.16
203.03	203.13	E_u	0.58	0.00	0.03	1.97	4.26
304.71	305.14	A_{1g}	0.00	2.69	0.54	1.29	2.41
339.46	339.44	A_{2u}	0.07	0.00	1.64	3.45	6.29

Table S5 Spectroscopic data for SnS_2. The first and second columns compare the frequencies obtained from density-functional perturbation theory (DFPT) and finite-displacement (FD) calculations. The doubly-degenerate modes are identified based on the phonon frequencies, and the intensities and linewidths given are the average of the two modes in each pair.

ν_{DFPT} [cm-1]	ν_{FD} [cm-1]	Ir. Rep.	I_{IR} [e^2 amu^{-1}]	I_{Raman} [10^4 Å^4 amu^{-1}]	$\Gamma_{10\mathrm{K}}$ [cm-1]	$\Gamma_{150\mathrm{K}}$ [cm-1]	$\Gamma_{300\mathrm{K}}$ [cm-1]
40.83	39.47	A_g	0.00	0.04	0.00	0.00	0.00
48.14	48.08	B_{2g}	0.00	0.02	0.00	0.00	0.00
54.63	54.59	A_u	0.00	0.00	0.00	0.00	0.00
66.27	65.32	B_{2u}	0.01	0.00	0.14	2.51	5.14
66.47	66.45	B_{3g}	0.00	0.06	0.01	0.46	0.94
66.89	66.57	B_{1g}	0.00	0.01	0.00	0.30	0.67
80.59	79.72	B_{1g}	0.00	0.00	0.11	1.42	2.87
91.55	91.31	A_g	0.00	0.30	0.01	0.28	0.60
96.83	96.42	B_{3u}	0.16	0.00	0.24	2.23	4.46
140.52	140.65	B_{1u}	2.90	0.00	0.60	3.97	8.02
160.70	160.89	B_{2g}	0.00	1.38	0.30	1.93	3.86
174.63	174.35	B_{3u}	1.72	0.00	0.17	1.05	2.13
179.55	179.51	B_{2u}	2.31	0.00	0.36	6.28	13.23
188.50	188.63	A_g	0.00	3.70	0.45	3.01	6.17
188.97	189.17	A_u	0.00	0.00	1.01	7.16	14.58
203.23	203.26	B_{3g}	0.00	0.00	0.76	13.57	27.56
208.92	209.09	B_{2u}	0.10	0.00	1.26	15.74	32.63
218.30	218.43	B_{1g}	0.00	0.11	0.16	1.43	2.98
219.96	220.10	A_g	0.00	2.17	0.09	0.71	1.46
221.82	221.97	B_{3u}	0.20	0.00	0.29	1.20	2.37
274.88	274.78	B_{1g}	0.00	0.07	0.67	3.71	7.38

Table S6 Spectroscopic data for *Pnma* SnS. The first and second columns compare the frequencies obtained from density-functional perturbation theory (DFPT) and finite-displacement (FD) calculations.

ν_{DFPT} [cm-1]	ν_{FD} [cm-1]	Ir. Rep.	I_{IR} [e^2 amu^{-1}]	I_{Raman} [10^4 Å^4 amu^{-1}]	Γ_{10K} [cm-1]	Γ_{150K} [cm-1]	Γ_{300K} [cm-1]
35.82	36.84	E	0.00	0.09	0.00	0.36	0.76
36.38	37.03	T	0.03	0.00	0.00	0.48	1.02
39.25	42.10	A	0.00	0.21	0.01	0.89	1.86
43.20	42.18	T	0.01	0.01	0.00	0.44	0.93
47.12	47.12	T	0.01	0.01	0.00	0.37	0.80
48.05	47.08	E	0.00	0.04	0.00	0.44	0.94
49.17	47.43	A	0.00	0.13	0.00	0.40	0.85
50.16	50.48	T	0.00	0.00	0.01	0.51	1.08
50.81	51.49	T	0.02	0.00	0.01	0.38	0.81
53.54	54.07	T	0.00	0.01	0.01	0.30	0.64
55.69	55.81	E	0.00	0.03	0.01	0.54	1.14
55.83	56.19	T	0.00	0.00	0.01	0.36	0.77
58.34	57.52	T	0.00	0.01	0.01	0.32	0.70
59.31	57.95	E	0.00	0.21	0.01	0.39	0.85
61.29	61.00	T	0.00	0.00	0.02	0.45	0.96
62.09	63.11	A	0.00	0.06	0.02	0.38	0.83
63.81	62.99	T	0.00	0.00	0.02	0.40	0.87
66.05	67.04	E	0.00	0.31	0.02	0.31	0.67
67.62	66.94	A	0.00	0.24	0.02	0.33	0.70
68.65	68.68	T	0.03	0.01	0.03	0.41	0.86
71.06	71.05	T	0.01	0.01	0.03	0.37	0.78
71.37	70.46	E	0.00	0.01	0.04	0.45	0.92
72.17	72.30	T	0.03	0.00	0.03	0.33	0.70
78.50	78.54	T	0.00	0.00	0.06	0.52	1.07
79.08	78.93	A	0.00	0.13	0.12	0.83	1.68
81.10	80.42	E	0.00	0.04	0.06	0.54	1.12
83.56	84.35	A	0.00	0.62	0.09	0.63	1.29

Table S7 Spectroscopic data for π-cubic SnS. The first and second columns compare the frequencies obtained from density-functional perturbation theory (DFPT) and finite-displacement (FD) calculations. The doubly- and triply-degenerate modes are identified based on the phonon frequencies, and the intensities and linewidths given are the average of the modes in each set.

ν_{DFPT} [cm-1]	ν_{FD} [cm-1]	Ir. Rep.	I_{IR} [e^2 amu^{-1}]	I_{Raman} [10^4 Å^4 amu^{-1}]	Γ_{10K} [cm-1]	Γ_{150K} [cm-1]	Γ_{300K} [cm-1]
85.73	86.07	T	0.00	0.00	0.09	0.62	1.27
87.54	87.22	T	0.03	0.01	0.08	0.63	1.31
89.17	88.80	T	0.00	0.00	0.12	0.82	1.68
91.98	91.12	T	0.27	0.06	0.19	1.10	2.23
97.80	97.05	T	0.06	0.00	0.16	0.96	1.95
99.07	99.24	T	0.01	0.00	0.15	0.92	1.87
103.68	104.31	T	0.07	0.01	0.20	1.07	2.16
106.50	105.62	T	0.02	0.03	0.26	1.31	2.64
106.96	107.02	E	0.00	0.24	0.22	1.12	2.25
112.04	112.21	A	0.00	0.14	0.23	1.19	2.40
115.90	115.98	T	0.00	0.07	0.19	1.05	2.14
117.78	116.88	A	0.00	0.42	0.44	2.21	4.44
165.22	165.39	A	0.00	0.03	0.28	4.11	8.59
165.78	165.75	E	0.00	1.57	0.31	3.20	6.64
166.21	166.29	T	0.77	0.00	0.32	3.24	6.75
168.26	168.23	T	11.15	0.07	0.38	3.30	6.86
173.35	173.56	A	0.00	19.70	0.35	3.55	7.37
174.22	174.38	T	1.32	0.01	0.28	2.24	4.64
176.99	177.23	E	0.00	0.47	0.15	1.39	2.89
177.74	177.85	T	0.04	0.00	0.20	2.14	4.48
179.69	179.76	T	0.77	0.00	0.31	2.24	4.62
183.30	183.31	E	0.00	1.51	0.28	2.22	4.59
186.65	186.66	A	0.00	8.07	0.23	2.21	4.59
194.79	194.66	T	0.06	0.00	0.23	2.95	6.18
197.45	197.48	T	0.92	0.01	0.13	1.48	3.09
200.56	200.52	T	0.52	0.00	0.16	1.62	3.35
201.83	201.79	T	0.94	0.00	0.16	1.69	3.52

Table S7 Spectroscopic data for π-cubic SnS. The first and second columns compare the frequencies obtained from density-functional perturbation theory (DFPT) and finite-displacement (FD) calculations. The doubly- and triply-degenerate modes are identified based on the phonon frequencies, and the intensities and linewidths given are the average of the modes in each set.

ν_{DFPT} [cm^{-1}]	ν_{FD} [cm^{-1}]	Ir. Rep.	I_{IR} [e^2 amu^{-1}]	I_{Raman} [10^4 Å^4 amu^{-1}]	Γ_{10K} [cm^{-1}]	Γ_{150K} [cm^{-1}]	Γ_{300K} [cm^{-1}]
202.30	202.18	A	0.00	7.95	0.22	2.43	5.07
206.92	206.91	T	0.08	0.01	0.16	1.31	2.69
208.86	208.96	T	0.08	0.05	0.16	1.33	2.73
209.75	209.73	E	0.00	0.06	0.16	1.70	3.54
212.21	212.30	T	0.08	0.02	0.18	1.09	2.20
214.44	214.50	E	0.00	0.09	0.21	1.18	2.37
218.59	218.55	A	0.00	0.02	0.25	1.56	3.15
219.57	219.59	E	0.00	0.30	0.45	2.01	3.98
219.60	219.61	T	0.01	0.21	0.36	1.57	3.10
220.60	220.59	A	0.00	0.82	0.52	2.11	4.15
222.21	222.20	T	0.03	0.00	0.86	2.95	5.73
224.17	224.19	T	0.01	0.00	0.79	2.69	5.22
226.80	226.83	T	0.03	0.00	0.81	2.66	5.13
228.66	228.70	E	0.00	0.12	0.71	2.34	4.52
228.69	228.69	T	0.00	0.04	0.72	2.42	4.68
238.94	238.95	T	0.04	0.01	1.03	2.94	5.61
241.92	241.93	T	0.04	0.01	0.96	2.74	5.22
248.82	248.82	T	0.10	0.03	1.24	3.39	6.43
252.03	252.09	T	0.01	0.12	1.65	4.44	8.41
256.59	256.66	A	0.00	0.04	1.89	5.09	9.64
256.73	256.79	E	0.00	0.11	2.03	5.27	9.94
256.89	256.91	T	0.02	0.00	1.52	3.92	7.40
262.69	262.72	A	0.00	0.09	1.65	4.12	7.76
266.29	266.21	T	0.04	0.00	1.74	4.32	8.12
269.31	269.28	T	0.01	0.00	1.82	4.46	8.37

Table S7 Spectroscopic data for π-cubic SnS. The first and second columns compare the frequencies obtained from density-functional perturbation theory (DFPT) and finite-displacement (FD) calculations. The doubly- and triply-degenerate modes are identified based on the phonon frequencies, and the intensities and linewidths given are the average of the modes in each set.

ν_{DFPT} [cm^{-1}]	ν_{FD} [cm^{-1}]	Ir. Rep.	I_{IR} [e^2 amu^{-1}]	I_{Raman} [10^4 Å^4 amu^{-1}]	Γ_{10K} [cm^{-1}]	Γ_{150K} [cm^{-1}]	Γ_{300K} [cm^{-1}]
30.93	31.93	A_u	0.01	0.00	0.04	3.52	7.19
32.27	32.62	B_{1u}	0.00	0.00	0.27	17.98	36.74
41.60	44.50	B_{3u}	0.01	0.00	0.61	13.56	27.55
45.09	47.71	A_g	0.00	0.03	0.38	7.89	16.10
52.09	51.65	B_{1g}	0.00	0.02	0.11	2.12	4.38
52.60	54.79	B_{2g}	0.00	0.00	0.72	9.43	19.40
55.04	55.47	B_{3g}	0.21	0.00	0.32	3.43	7.00
55.87	56.81	B_{1u}	0.00	0.00	2.85	26.53	53.34
57.99	59.44	A_g	0.00	0.06	2.64	21.46	43.23
60.57	60.85	B_{2g}	0.00	0.00	3.05	25.57	51.48
63.96	64.32	B_{3u}	0.04	0.00	4.36	39.59	80.64
69.22	68.98	B_{2u}	0.01	0.00	0.28	2.48	5.08
69.91	69.81	A_u	0.00	0.02	0.34	2.66	5.45
70.03	70.33	A_g	0.00	0.00	4.61	33.59	67.90
78.55	78.51	B_{1g}	0.00	0.01	0.28	2.26	4.74
78.99	78.93	B_{3g}	0.00	0.00	0.46	3.47	7.14
79.73	81.16	B_{2g}	0.00	0.11	1.16	9.06	18.41
111.11	111.73	B_{3u}	0.00	0.00	1.46	8.35	17.07
115.61	116.33	B_{1u}	0.17	0.00	1.76	10.54	21.68
145.69	145.91	A_g	0.00	0.11	1.70	10.48	21.35
148.58	148.76	B_{2g}	0.00	0.00	2.36	12.73	25.83
178.72	178.68	B_{2u}	4.11	0.00	0.34	4.41	9.22
179.35	179.41	B_{1u}	1.01	0.00	1.50	11.36	23.20
181.50	181.55	A_g	0.00	1.08	5.20	27.52	55.63
185.59	185.57	A_u	0.00	0.00	0.18	2.33	4.92
185.84	185.89	B_{3u}	0.45	0.00	1.88	10.48	21.35
187.93	187.92	B_{2u}	0.04	0.00	0.49	3.72	7.71
189.30	189.30	A_u	0.00	0.00	0.35	4.46	9.38
191.24	191.23	B_{1g}	0.00	0.03	0.28	3.91	8.20

Table S8 Spectroscopic data for Sn_2S_3. The first and second columns compare the frequencies obtained from density-functional perturbation theory (DFPT) and finite-displacement (FD) calculations.

ν_{DFPT} [cm^{-1}]	ν_{FD} [cm^{-1}]	Ir. Rep.	I_{IR} [e^2 amu^{-1}]	I_{Raman} [10^4 Å^4 amu^{-1}]	Γ_{10K} [cm^{-1}]	Γ_{150K} [cm^{-1}]	Γ_{300K} [cm^{-1}]
193.98	194.06	B_{2g}	0.00	0.00	1.48	12.24	25.11
200.23	200.23	B_{3g}	0.00	0.04	0.59	3.84	7.89
209.09	209.09	B_{3g}	0.00	0.03	0.35	4.34	9.10
209.54	209.54	B_{1g}	0.00	0.19	0.37	4.37	9.17
212.78	212.91	B_{3u}	2.48	0.00	2.12	15.51	31.80
220.91	220.90	B_{2u}	0.34	0.00	1.26	8.09	16.46
222.48	222.47	A_u	0.00	0.00	1.08	8.77	18.08
223.10	223.18	B_{1u}	0.00	0.00	1.80	12.24	25.02
226.13	226.14	A_g	0.00	0.96	5.90	46.83	97.68
243.96	243.93	B_{1g}	0.00	0.18	1.98	8.14	16.07
244.51	244.48	B_{2g}	0.00	0.09	3.48	18.69	37.45
244.64	244.62	B_{3g}	0.00	0.02	1.87	9.92	19.92
252.00	252.06	A_g	0.00	0.66	2.89	14.81	29.45
258.35	258.40	B_{3u}	0.00	0.00	3.09	14.44	28.59
258.64	258.68	B_{2g}	0.00	0.01	2.77	22.10	44.76
263.77	263.79	B_{3u}	0.33	0.00	2.85	12.17	23.92
264.71	264.76	B_{1u}	0.33	0.00	1.73	9.85	19.53
278.57	278.63	A_g	0.00	0.10	8.09	27.83	53.51
279.82	279.84	B_{1u}	0.42	0.00	8.58	26.50	50.48
282.21	282.23	B_{1u}	0.23	0.00	3.32	10.95	20.94
282.97	282.95	B_{2g}	0.00	0.15	4.54	14.44	27.59
283.85	283.86	B_{3u}	0.17	0.00	4.20	13.89	26.57
289.04	289.07	B_{1u}	0.32	0.00	6.23	18.68	35.42
291.08	291.04	A_g	0.00	15.02	33.85	84.72	158.51
291.19	291.08	B_{2g}	0.00	0.05	10.26	28.22	53.18
300.01	300.06	A_g	0.00	3.16	8.71	25.90	48.99
307.60	307.64	B_{3u}	0.01	0.00	8.86	24.25	45.54
309.07	309.07	B_{2g}	0.00	0.01	9.04	32.53	62.12

Table S8 Spectroscopic data for Sn_2S_3. The first and second columns compare the frequencies obtained from density-functional perturbation theory (DFPT) and finite-displacement (FD) calculations.

3. Mode eigenvectors of the *Pnma* and π-cubic SnS and Sn_2S_3

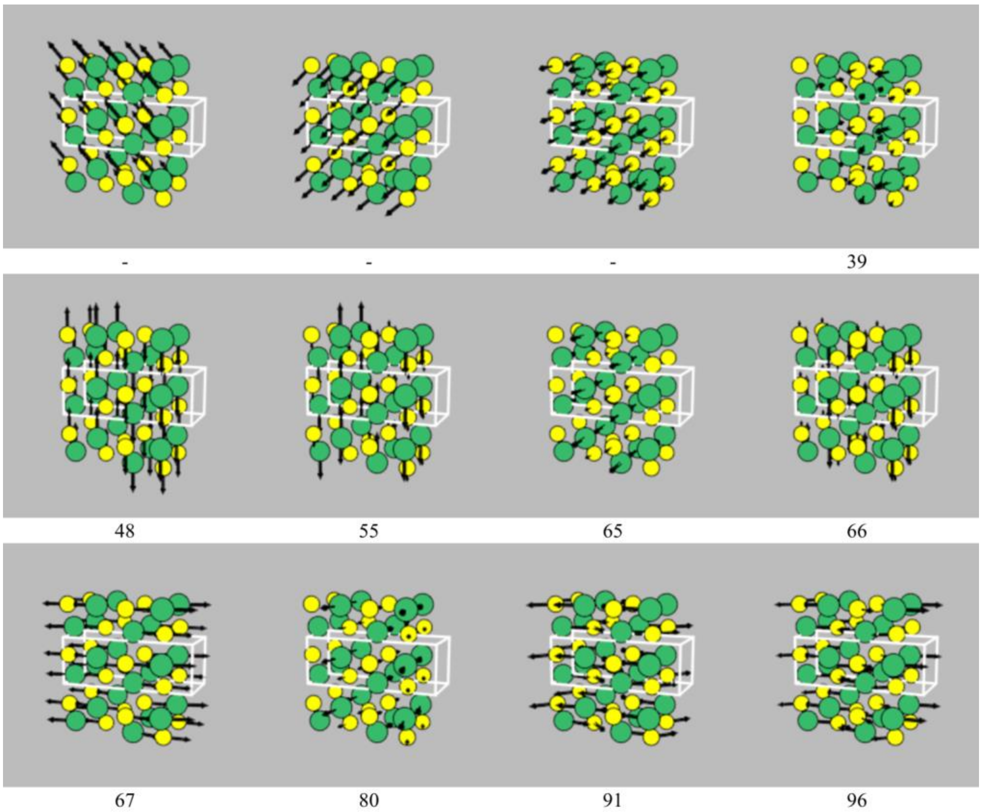

Figure S1 Eigenvectors of the 24 Γ-point phonon modes of *Pnma* SnS with frequencies as marked (cm^{-1}). The Sn and S atoms are coloured green and yellow, respectively. The three acoustic modes, which correspond to rigid translations of the crystal lattice, necessarily have zero frequency and are spectroscopically inactive, and so the frequencies of these modes are not shown. These images were generated using the ascii-phonons software.[4]

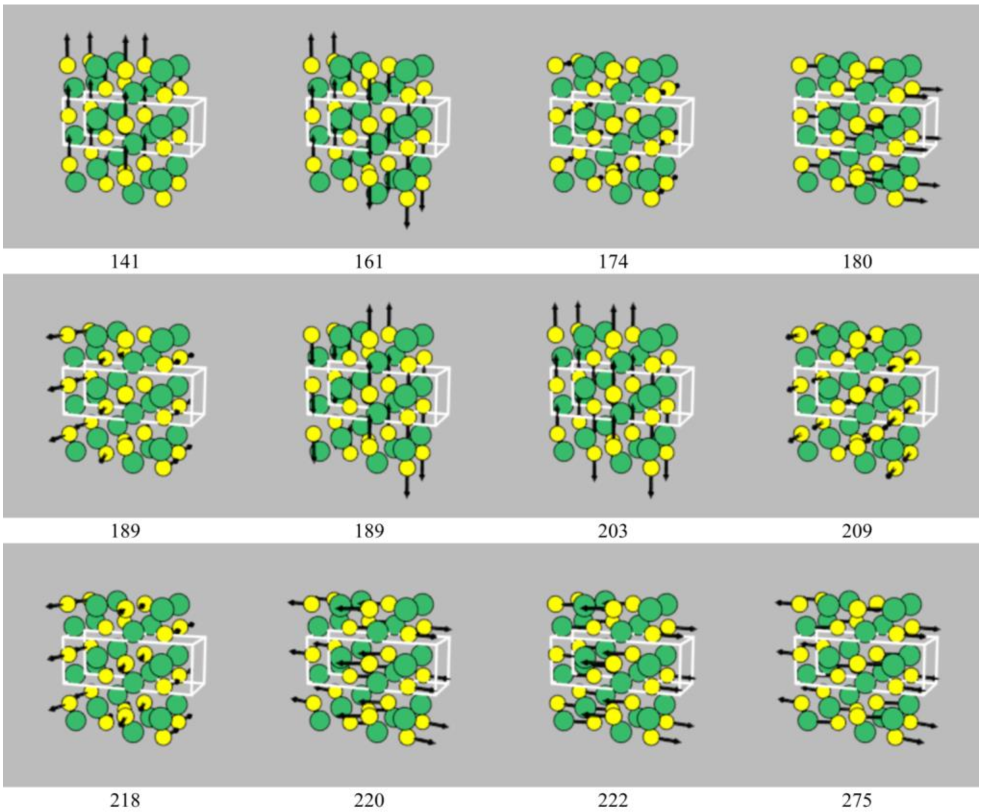

Figure S1 Eigenvectors of the 24 Γ-point phonon modes of *Pnma* SnS with frequencies as marked (cm^{-1}). The Sn and S atoms are coloured green and yellow, respectively. The three acoustic modes, which correspond to rigid translations of the crystal lattice, necessarily have zero frequency and are spectroscopically inactive, and so the frequencies of these modes are not shown. These images were generated using the ascii-phonons software.[4]

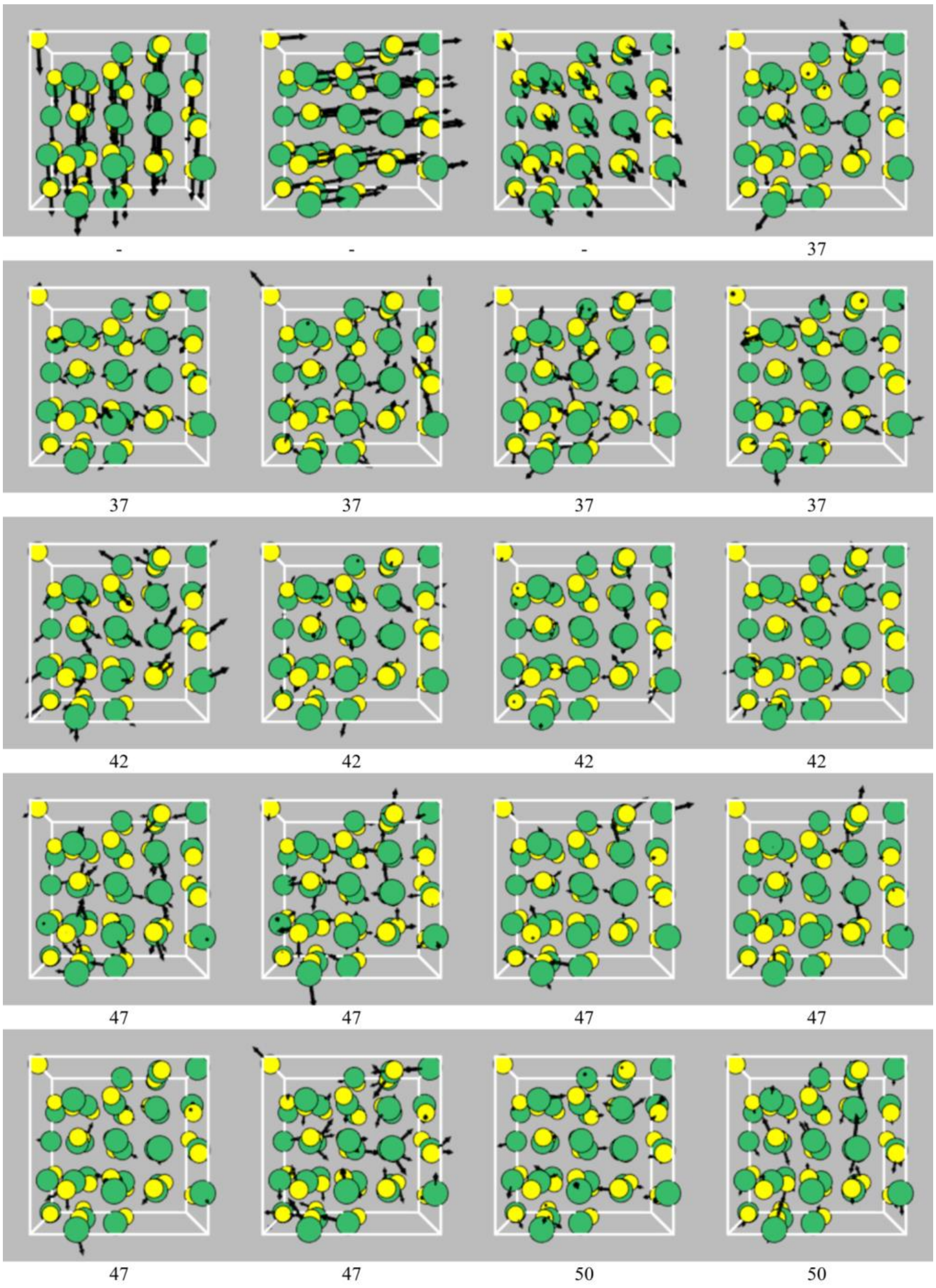

Figure S2 Eigenvectors of the 192 Γ-point phonon modes of π-cubic SnS with frequencies as marked (cm^{-1}). The Sn and S atoms are coloured green and yellow, respectively. The three acoustic modes, which correspond to rigid translations of the crystal lattice, necessarily have zero frequency and are spectroscopically inactive, and so the frequencies of these modes are not shown. These images were generated using the ascii-phonons software.[4]

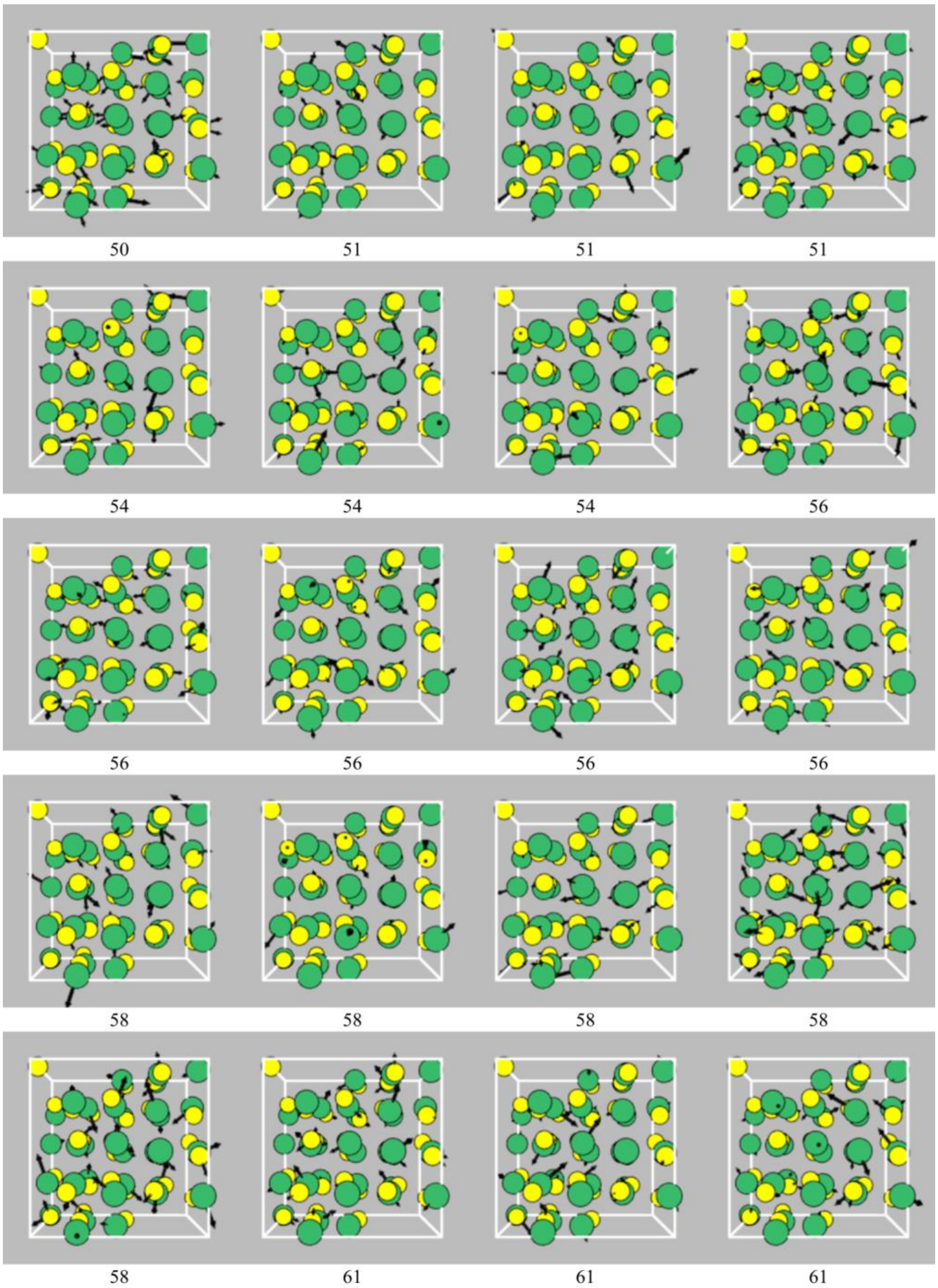

Figure S2 Eigenvectors of the 192 Γ-point phonon modes of π-cubic SnS with frequencies as marked (cm^{-1}). The Sn and S atoms are coloured green and yellow, respectively. The three acoustic modes, which correspond to rigid translations of the crystal lattice, necessarily have zero frequency and are spectroscopically inactive, and so the frequencies of these modes are not shown. These images were generated using the ascii-phonons software.[4]

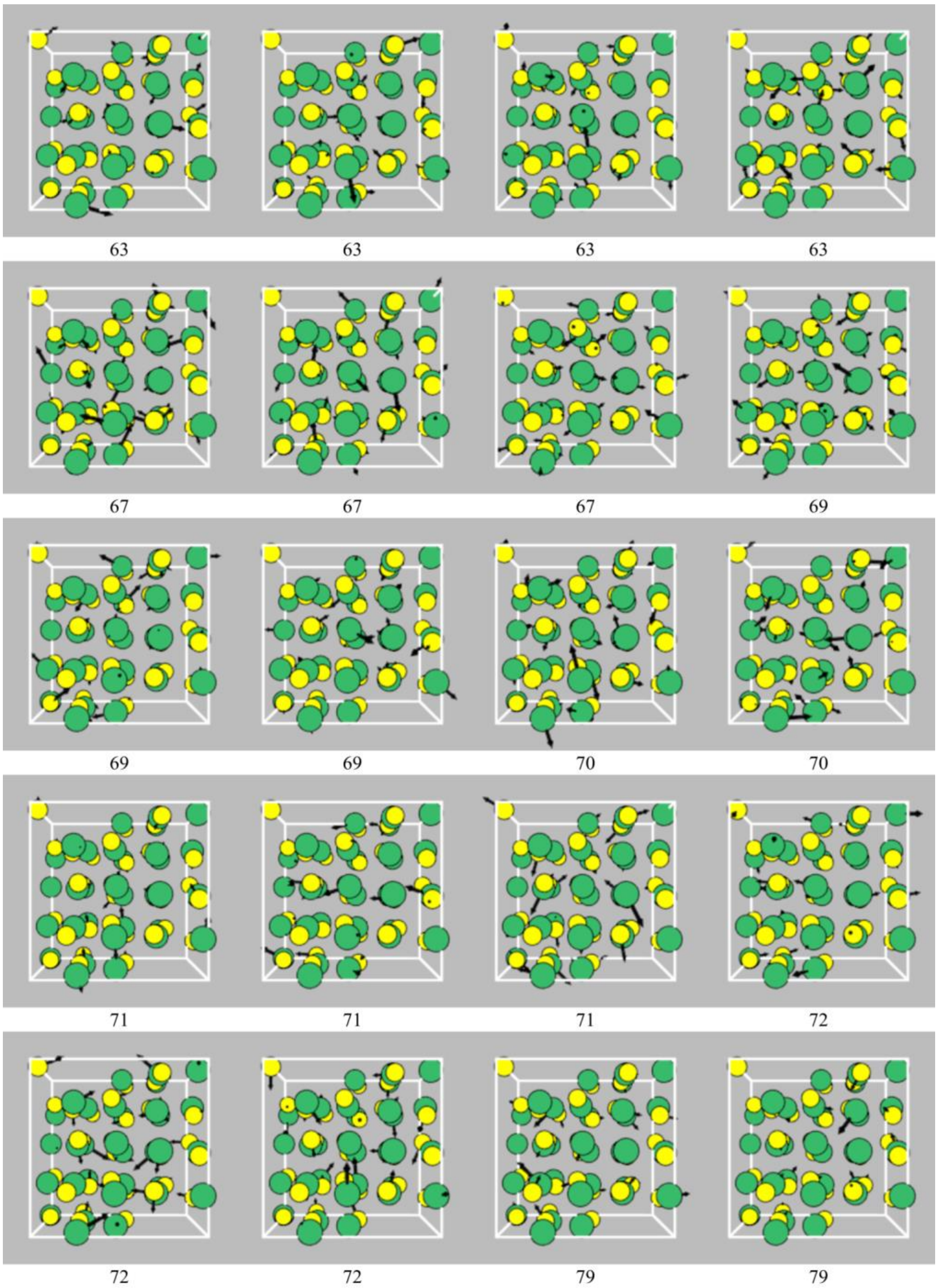

Figure S2 Eigenvectors of the 192 Γ-point phonon modes of π-cubic SnS with frequencies as marked (cm^{-1}). The Sn and S atoms are coloured green and yellow, respectively. The three acoustic modes, which correspond to rigid translations of the crystal lattice, necessarily have zero frequency and are spectroscopically inactive, and so the frequencies of these modes are not shown. These images were generated using the ascii-phonons software.[4]

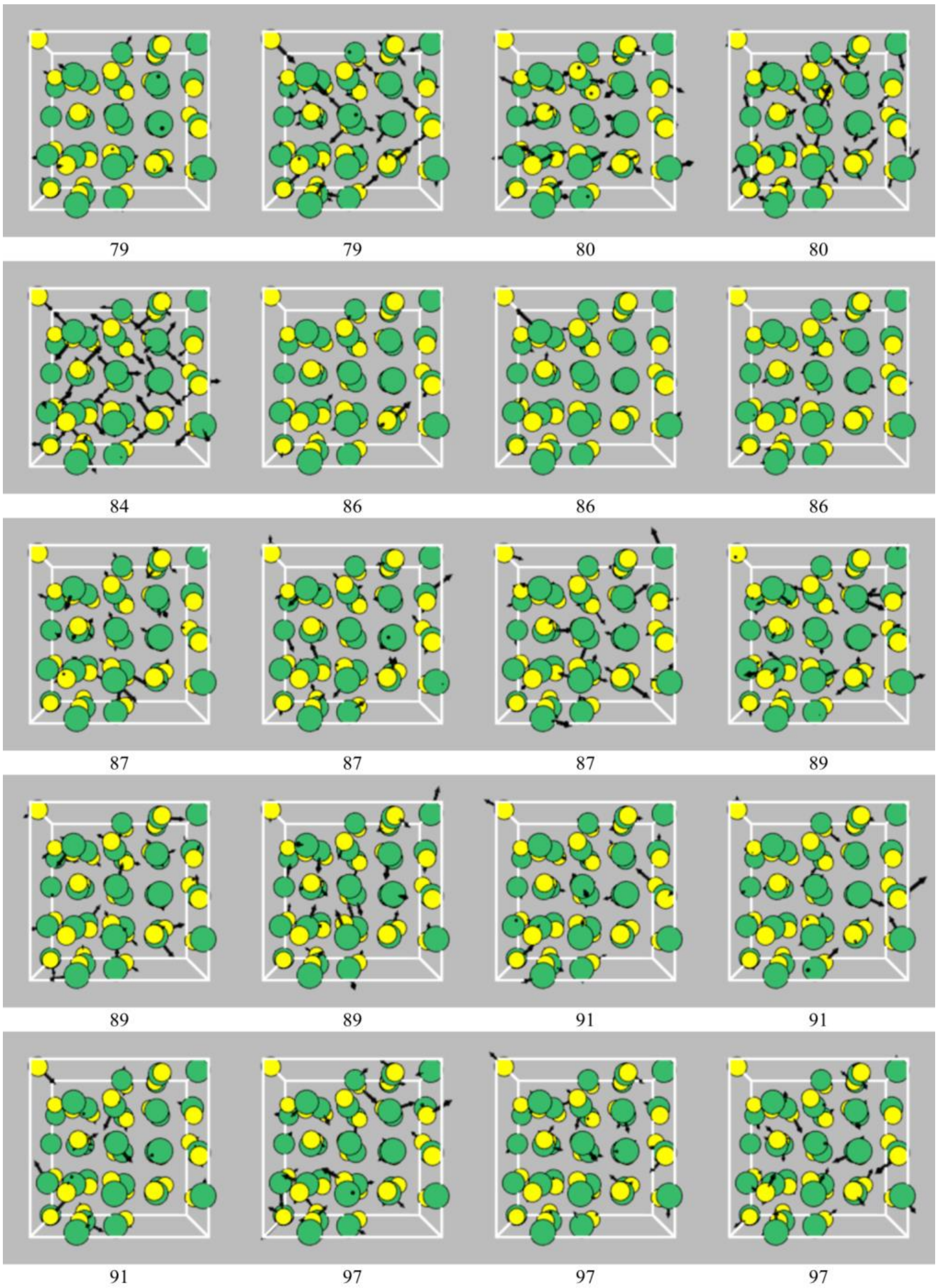

Figure S2 Eigenvectors of the 192 Γ-point phonon modes of π-cubic SnS with frequencies as marked (cm^{-1}). The Sn and S atoms are coloured green and yellow, respectively. The three acoustic modes, which correspond to rigid translations of the crystal lattice, necessarily have zero frequency and are spectroscopically inactive, and so the frequencies of these modes are not shown. These images were generated using the ascii-phonons software.[4]

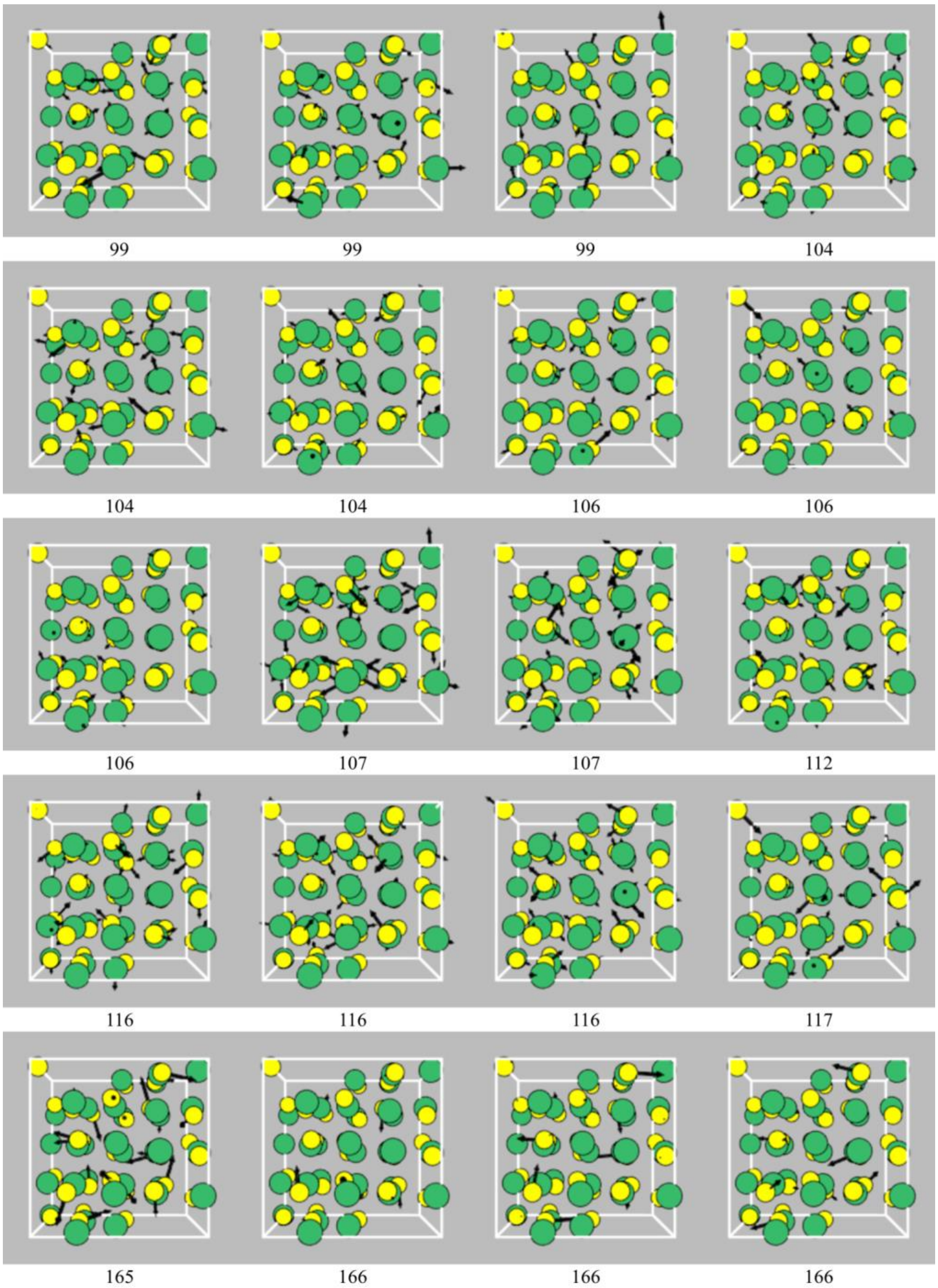

Figure S2 Eigenvectors of the 192 Γ-point phonon modes of π-cubic SnS with frequencies as marked (cm^{-1}). The Sn and S atoms are coloured green and yellow, respectively. The three acoustic modes, which correspond to rigid translations of the crystal lattice, necessarily have zero frequency and are spectroscopically inactive, and so the frequencies of these modes are not shown. These images were generated using the ascii-phonons software.[4]

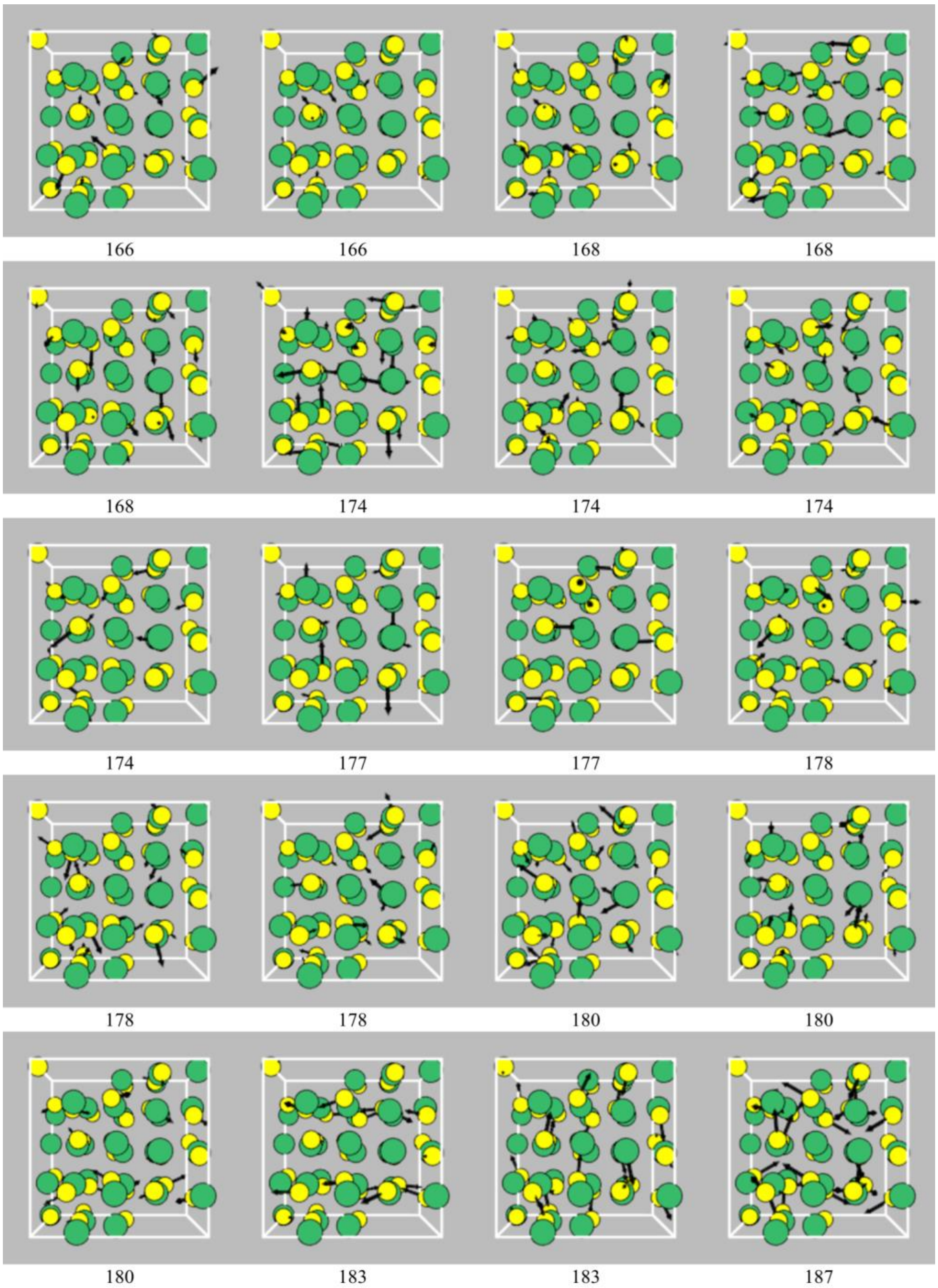

Figure S2 Eigenvectors of the 192 Γ-point phonon modes of π-cubic SnS with frequencies as marked (cm^{-1}). The Sn and S atoms are coloured green and yellow, respectively. The three acoustic modes, which correspond to rigid translations of the crystal lattice, necessarily have zero frequency and are spectroscopically inactive, and so the frequencies of these modes are not shown. These images were generated using the ascii-phonons software.[4]

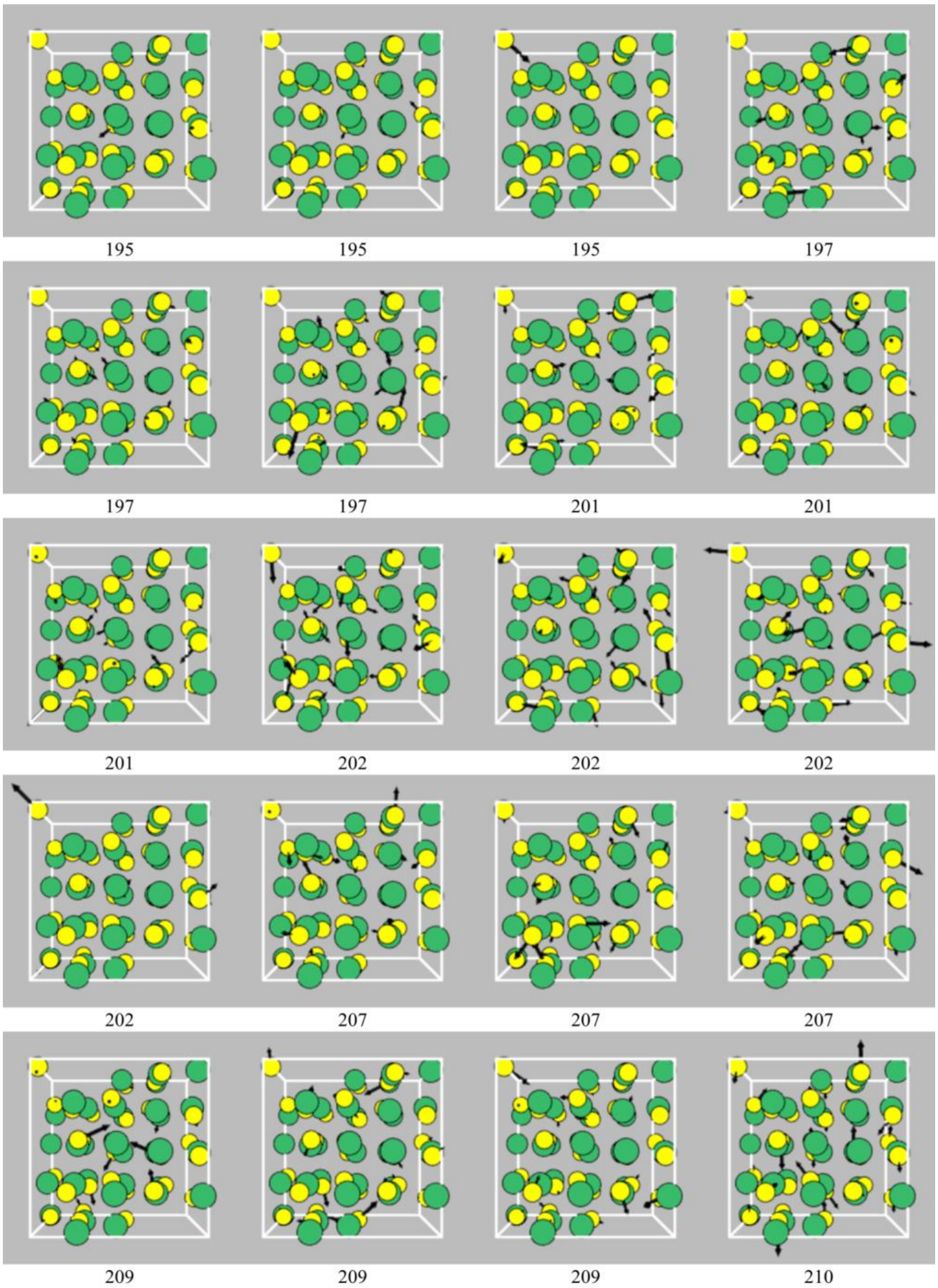

Figure S2 Eigenvectors of the 192 Γ-point phonon modes of π-cubic SnS with frequencies as marked (cm^{-1}). The Sn and S atoms are coloured green and yellow, respectively. The three acoustic modes, which correspond to rigid translations of the crystal lattice, necessarily have zero frequency and are spectroscopically inactive, and so the frequencies of these modes are not shown. These images were generated using the ascii-phonons software.[4]

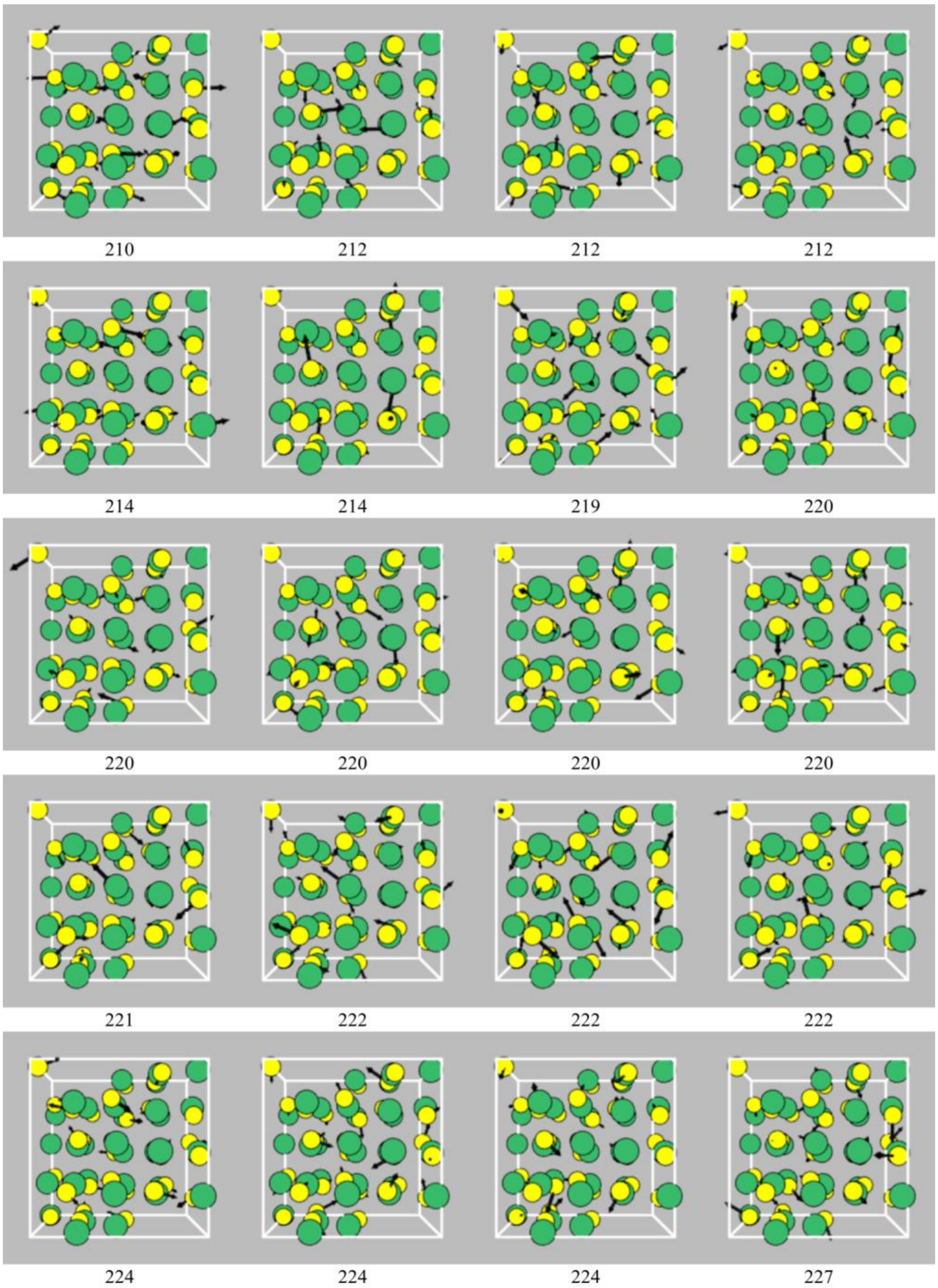

Figure S2 Eigenvectors of the 192 Γ-point phonon modes of π-cubic SnS with frequencies as marked (cm^{-1}). The Sn and S atoms are coloured green and yellow, respectively. The three acoustic modes, which correspond to rigid translations of the crystal lattice, necessarily have zero frequency and are spectroscopically inactive, and so the frequencies of these modes are not shown. These images were generated using the ascii-phonons software.[4]

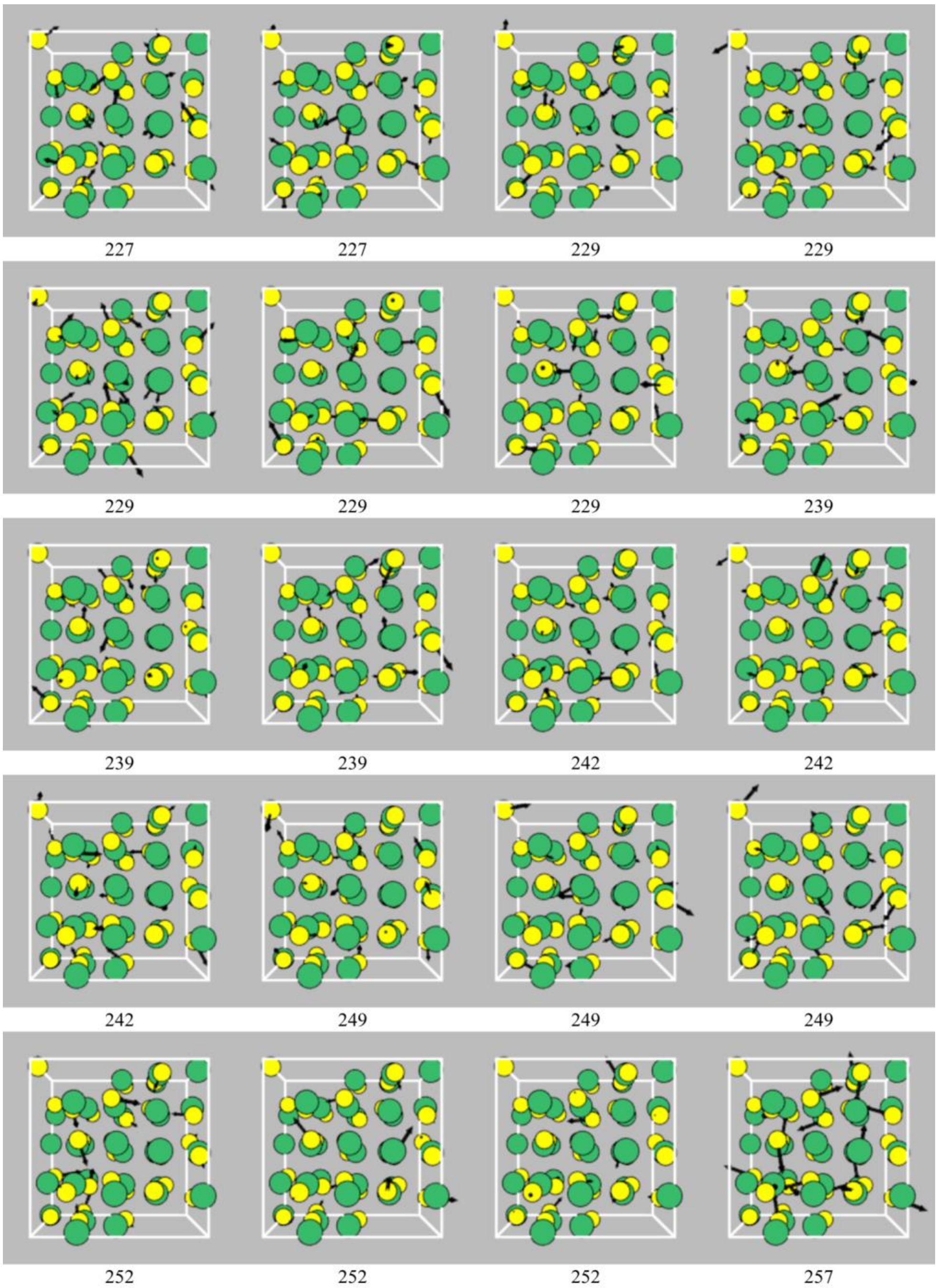

Figure S2 Eigenvectors of the 192 Γ-point phonon modes of π-cubic SnS with frequencies as marked (cm^{-1}). The Sn and S atoms are coloured green and yellow, respectively. The three acoustic modes, which correspond to rigid translations of the crystal lattice, necessarily have zero frequency and are spectroscopically inactive, and so the frequencies of these modes are not shown. These images were generated using the ascii-phonons software.[4]

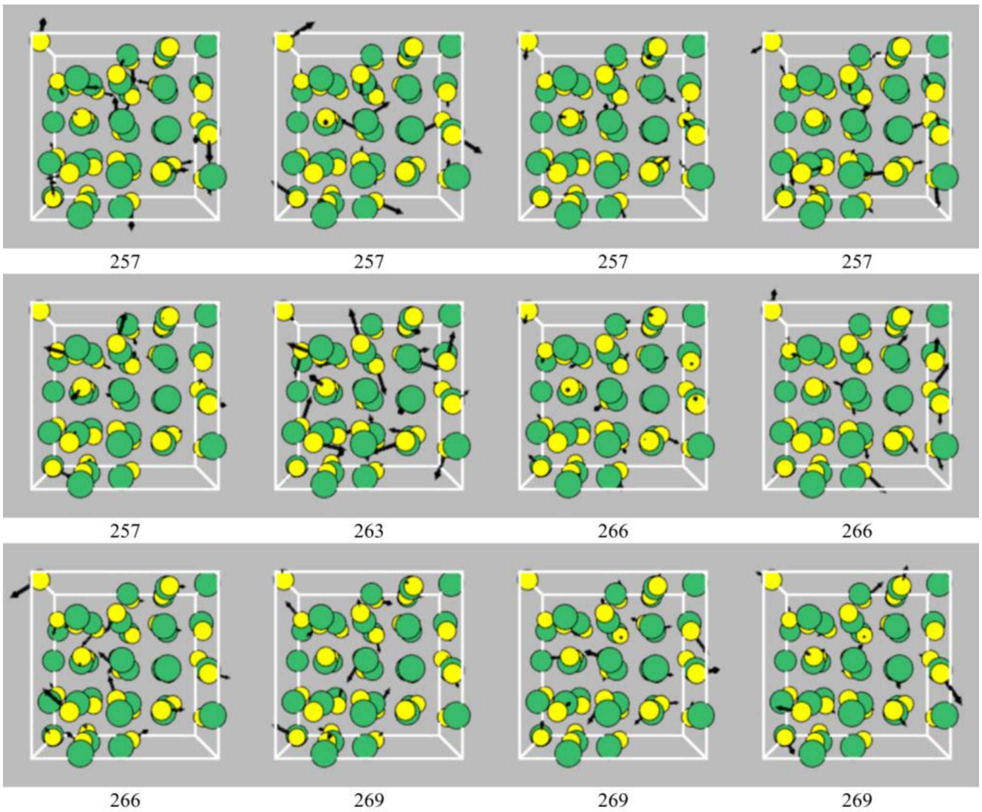

Figure S2 Eigenvectors of the 192 Γ-point phonon modes of π-cubic SnS with frequencies as marked (cm^{-1}). The Sn and S atoms are coloured green and yellow, respectively. The three acoustic modes, which correspond to rigid translations of the crystal lattice, necessarily have zero frequency and are spectroscopically inactive, and so the frequencies of these modes are not shown. These images were generated using the ascii-phonons software.[4]

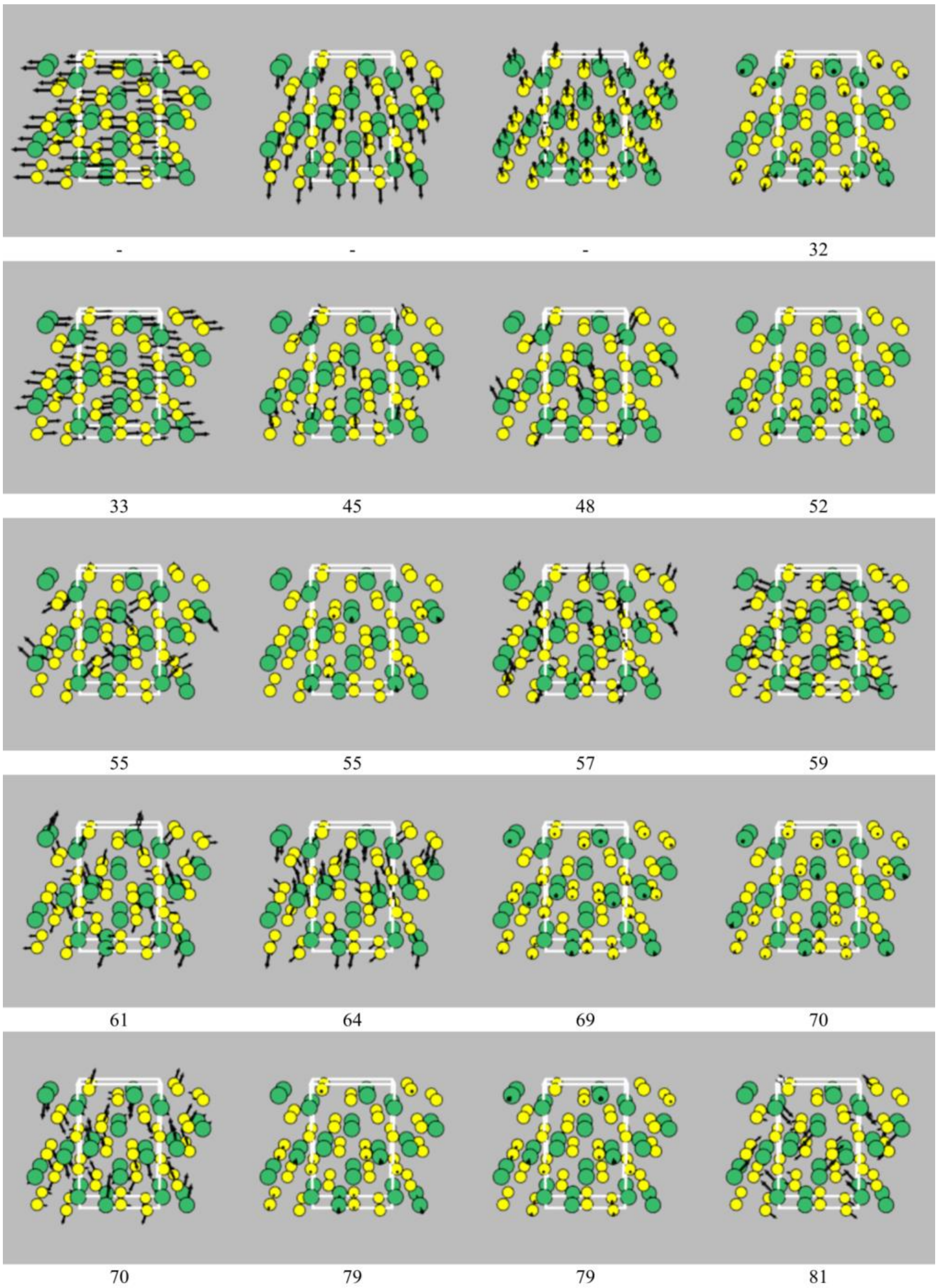

Figure S3 Eigenvectors of the 60 Γ-point phonon modes of Sn_2S_3 with frequencies as marked (cm^{-1}). The Sn and S atoms are coloured green and yellow, respectively. The three acoustic modes, which correspond to rigid translations of the crystal lattice, necessarily have zero frequency and are spectroscopically inactive, and so the frequencies of these modes are not shown. These images were generated using the ascii-phonons software.[4]

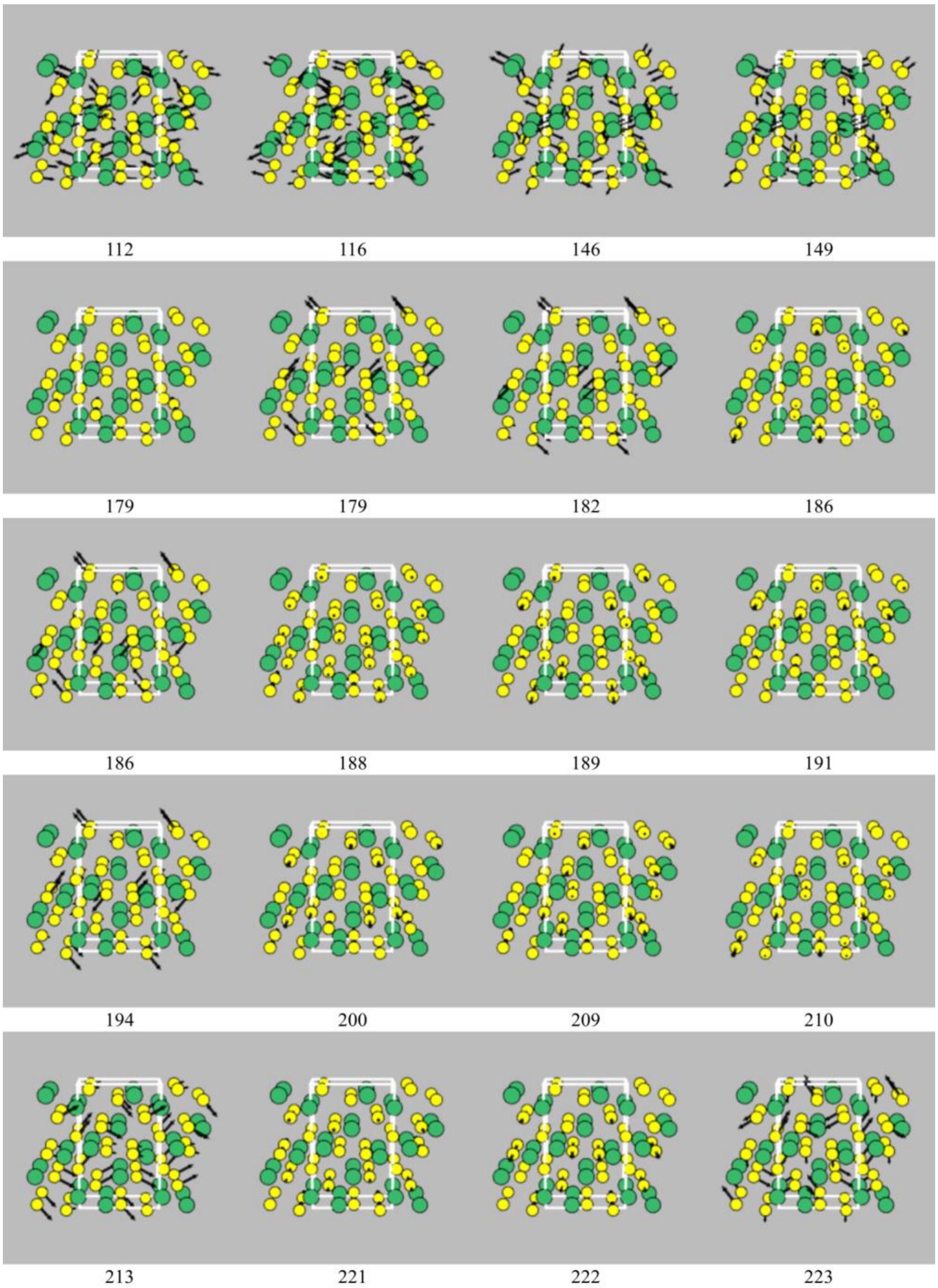

Figure S3 Eigenvectors of the 60 Γ-point phonon modes of Sn_2S_3 with frequencies as marked (cm^{-1}). The Sn and S atoms are coloured green and yellow, respectively. The three acoustic modes, which correspond to rigid translations of the crystal lattice, necessarily have zero frequency and are spectroscopically inactive, and so the frequencies of these modes are not shown. These images were generated using the ascii-phonons software.[4]

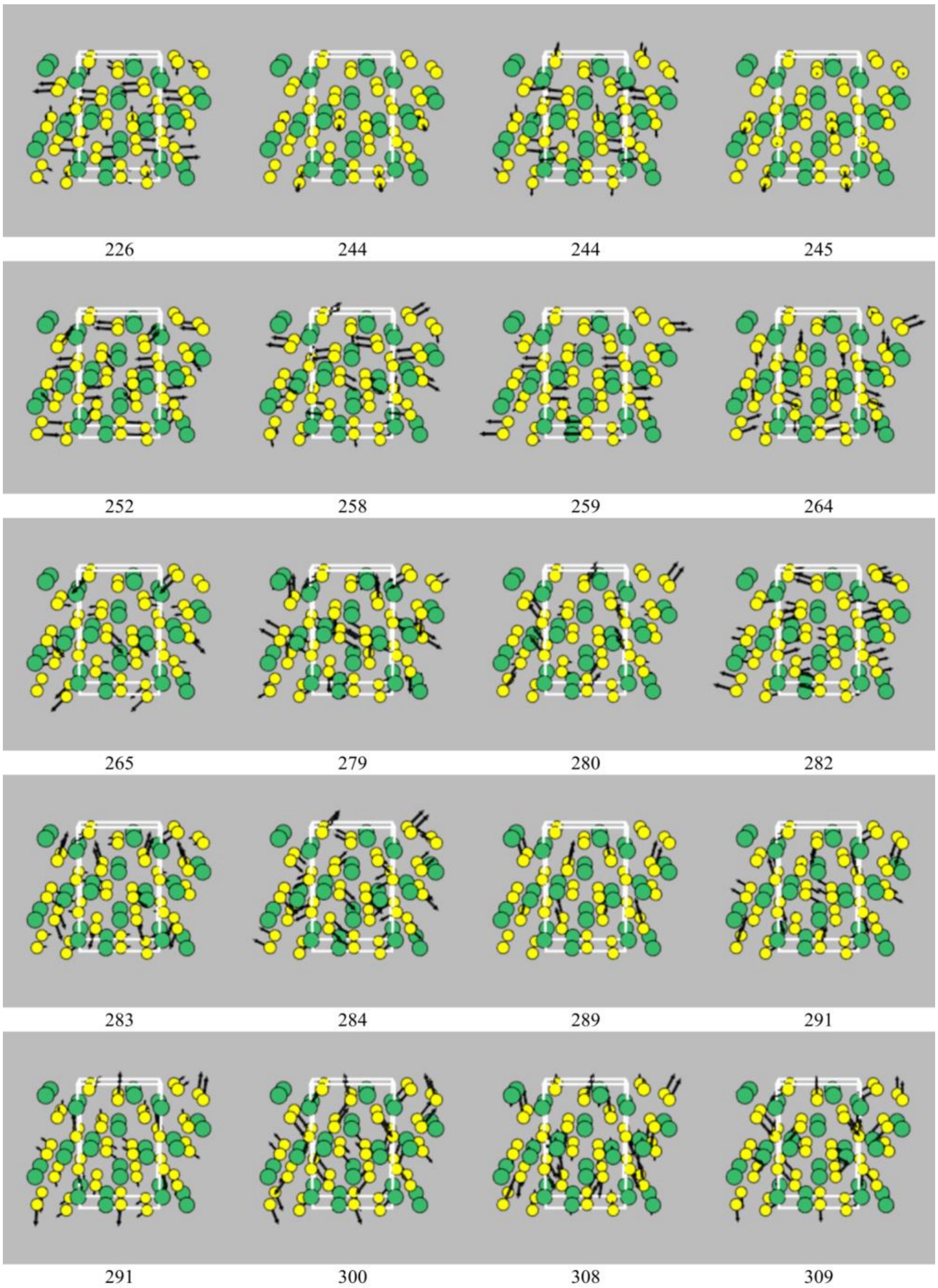

Figure S3 Eigenvectors of the 60 Γ-point phonon modes of Sn_2S_3 with frequencies as marked (cm^{-1}). The Sn and S atoms are coloured green and yellow, respectively. The three acoustic modes, which correspond to rigid translations of the crystal lattice, necessarily have zero frequency and are spectroscopically inactive, and so the frequencies of these modes are not shown. These images were generated using the ascii-phonons software.[4]

4. Dependence of the calculated Raman intensities on the displacement-step size

In principle, the calculated Raman intensities obtained by performing small steps along the mode eigenvectors may be sensitive to the choice of the step size used to compute the polarisability derivatives. This can be a particular issue for systems with small bandgaps at the generalised-gradient approximation (GGA) level of theory used in these calculations, for which certain atomic displacements (or combinations of displacements) could lead to disproportionately large changes in the electronic structure.[5]

To check this, we computed the Raman intensities of the four compounds using three different step sizes, *viz.* 0.005, 0.01 and 0.015 $\mathrm{amu}^{\frac{1}{2}}$ Å (Tables S9-S12). For Sn_2S_3, we also calculated the intensities for the 0.01 $\mathrm{amu}^{\frac{1}{2}}$ Å step using a denser **k**-point mesh (Table S12). As noted in the text, the Raman intensities were found for these systems to be relatively insensitive to the choice of the step size, with the various parameters yielding quantitatively very similar intensities.

We note that, due to the number of modes and the large size of the π-cubic model, when assessing the effect of the step size on the calculated mode intensities in this system we only performed calculations for a subset of the modes, *viz.* every 5th mode plus the ten modes with the largest intensities (49 modes in total; see Table S11).

ν_{DFPT} [cm^{-1}]	I_{Raman} [10^3 Å^4 amu^{-1}]		
	$\Delta Q(\nu)$ = 0.5 × 10^{-2} Å	$\Delta Q(\nu)$ =10^{-2} Å	$\Delta Q(\nu)$ = 1.5 × 10^{-2} Å
200.26	0.01	0.01	0.01
203.03	0.00	0.00	0.00
304.71	2.69	2.69	2.70
339.46	0.00	0.00	0.00

Table S9 Calculated Raman intensities of the Γ-point vibrational modes of SnS_2, using three different displacement steps along the eigenvectors to compute the polarizability derivatives. The doubly-degenerate modes are identified based on the phonon frequencies, and the intensities and linewidths given are the average of the two modes in each pair.

ν_{DFPT} [cm^{-1}]	I_{Raman} [10^4 Å^4 amu^{-1}]		
	$\Delta Q(\nu)$ = 0.5 × 10^{-2} Å	$\Delta Q(\nu)$ =10^{-2} Å	$\Delta Q(\nu)$ = 1.5 × 10^{-2} Å
40.83	0.04	0.04	0.04
48.14	0.02	0.02	0.02
54.63	0.00	0.00	0.00
66.27	0.00	0.00	0.00
66.47	0.06	0.06	0.06
66.89	0.01	0.01	0.01
80.59	0.00	0.00	0.00
91.55	0.30	0.30	0.30
96.83	0.00	0.00	0.00
140.52	0.00	0.00	0.00
160.70	1.38	1.38	1.38
174.63	0.00	0.00	0.00
179.55	0.00	0.00	0.00
188.50	3.70	3.70	3.70
188.97	0.00	0.00	0.00
203.23	0.00	0.00	0.00
208.92	0.00	0.00	0.00
218.30	0.11	0.11	0.11
219.96	2.17	2.17	2.17
221.82	0.00	0.00	0.00
274.88	0.07	0.07	0.07

Table S10 Calculated Raman intensities of the Γ-point vibrational modes of *Pnma* SnS, using three different displacement steps along the eigenvectors to compute the polarizability derivatives.

ν_{DFPT} [cm^{-1}]	I_{Raman} [10^4 Å^4 amu^{-1}] $\Delta Q(\nu)$ = 0.5 × 10^{-2} Å	$\Delta Q(\nu)$ =10^{-2} Å	$\Delta Q(\nu)$ = 1.5 × 10^{-2} Å
36.38	0.00	0.00	0.00
43.20	0.01	0.01	0.01
48.05	0.04	0.04	0.04
50.81	0.00	0.00	0.00
53.54	0.01	0.01	0.01
55.83	0.00	0.00	0.00
59.31	0.20	0.21	0.21
63.81	0.00	0.00	0.00
67.62	0.24	0.24	0.24
71.06	0.01	0.01	0.01
72.17	0.00	0.00	0.00
79.08	0.13	0.13	0.13
83.56	0.60	0.62	0.61
85.73	0.00	0.00	0.01
89.17	0.00	0.00	0.00
91.98	0.06	0.07	0.06
99.07	0.01	0.00	0.00
106.50	0.03	0.03	0.03
112.04	0.14	0.14	0.14
165.22	0.04	0.03	0.03
165.78	1.56	1.55	1.60
165.78	1.56	1.58	1.63
166.21	0.00	0.00	0.00
173.35	19.72	19.70	19.76
174.22	0.01	0.01	0.01
176.99	0.47	0.46	0.50
176.99	0.48	0.47	0.49
177.74	0.00	0.00	0.00
179.69	0.00	0.00	0.00
183.30	1.47	1.50	1.47
183.30	1.47	1.52	1.49
186.65	8.09	8.07	8.07
194.79	0.00	0.00	0.00
200.56	0.00	0.00	0.00
201.83	0.00	0.01	0.00
202.30	7.97	7.95	7.92
208.86	0.05	0.05	0.05
212.21	0.02	0.02	0.02
218.59	0.02	0.02	0.02
219.60	0.21	0.21	0.21

Table S11 Calculated Raman intensities of selected Γ-point vibrational modes of π-cubic SnS using three different displacement steps along the eigenvectors to compute the polarizability derivatives. The selection of modes includes every fifth non-acoustic mode, plus the ten modes with the highest calculated intensities (49 modes in total).

ν_{DFPT} [cm^{-1}]	I_{Raman} [10^3 Å^4 amu^{-1}]		
	$\Delta Q(\nu)$ = 0.5 × 10^{-2} Å	$\Delta Q(\nu)$ =10^{-2} Å	$\Delta Q(\nu)$ = 1.5 × 10^{-2} Å
220.60	0.80	0.82	0.82
224.17	0.00	0.00	0.00
226.80	0.00	0.00	0.00
228.69	0.03	0.04	0.04
241.92	0.01	0.01	0.00
252.03	0.13	0.11	0.11
256.73	0.10	0.11	0.10
266.29	0.00	0.00	0.00
269.31	0.01	0.00	0.01

Table S11 Calculated Raman intensities of selected Γ-point vibrational modes of π-cubic SnS using three different displacement steps along the eigenvectors to compute the polarizability derivatives. The selection of modes includes every fifth non-acoustic mode, plus the ten modes with the highest calculated intensities (49 modes in total).

v_{DFPT} [cm^{-1}]	I_{Raman} [10^4 Å^4 amu^{-1}]			
	$\Delta Q(v)$ = 0.5 × 10^{-2} Å	$\Delta Q(v)$ =10^{-2} Å	$\Delta Q(v)$ = 1.5 × 10^{-2} Å	$\Delta Q(V)$ = 0.01, **k** = 6×10×5
30.93	0.00	0.00	0.00	0.00
32.27	0.00	0.00	0.00	0.00
41.60	0.00	0.00	0.00	0.00
45.09	0.04	0.03	0.03	0.04
52.09	0.02	0.02	0.02	0.02
52.60	0.00	0.00	0.00	0.00
55.04	0.00	0.00	0.00	0.00
55.87	0.00	0.00	0.00	0.00
57.99	0.06	0.06	0.06	0.06
60.57	0.00	0.00	0.00	0.00
63.96	0.00	0.00	0.00	0.00
69.22	0.00	0.00	0.00	0.00
69.91	0.02	0.02	0.02	0.01
70.03	0.00	0.00	0.00	0.00
78.55	0.01	0.01	0.01	0.01
78.99	0.00	0.00	0.00	0.00
79.73	0.11	0.11	0.11	0.11
111.11	0.00	0.00	0.00	0.00
115.61	0.00	0.00	0.00	0.00
145.69	0.11	0.11	0.11	0.10
148.58	0.00	0.00	0.00	0.00
178.72	0.00	0.00	0.00	0.00
179.35	0.00	0.00	0.00	0.00
181.50	1.07	1.08	1.07	0.91
185.59	0.00	0.00	0.00	0.00
185.84	0.00	0.00	0.00	0.00
187.93	0.00	0.00	0.00	0.00
189.30	0.00	0.00	0.00	0.00
191.24	0.03	0.03	0.03	0.03
193.98	0.00	0.00	0.00	0.00
200.23	0.04	0.04	0.04	0.04
209.09	0.03	0.03	0.03	0.02
209.54	0.19	0.19	0.19	0.18
212.78	0.00	0.00	0.00	0.00
220.91	0.00	0.00	0.00	0.00
222.48	0.00	0.00	0.00	0.00
223.10	0.00	0.00	0.00	0.00
226.13	0.96	0.96	0.97	0.51
243.96	0.18	0.18	0.18	0.18
244.51	0.09	0.09	0.09	0.06

Table S12 Calculated Raman intensities of the Γ-point vibrational modes of Sn_2S_3, using three different displacement steps along the eigenvectors, and also a denser **k**-point sampling mesh with $\Delta Q(V)$ = 0.01, to compute the polarizability derivatives.

	I_{Raman} [10^4 Å^4 amu^{-1}]			
v_{DFPT} [cm^{-1}]	$\Delta Q(v)$ = 0.5 × 10^{-2} Å	$\Delta Q(v)$ =10^{-2} Å	$\Delta Q(v)$ = 1.5 × 10^{-2} Å	$\Delta Q(v)$ = 10^{-2} Å, **k** = 6×10×5
244.64	0.02	0.02	0.02	0.02
252.00	0.66	0.66	0.66	0.65
258.35	0.00	0.00	0.00	0.00
258.64	0.01	0.01	0.01	0.01
263.77	0.00	0.00	0.00	0.00
264.71	0.00	0.00	0.00	0.00
278.57	0.10	0.10	0.10	0.08
279.82	0.00	0.00	0.00	0.00
282.21	0.00	0.00	0.00	0.00
282.97	0.15	0.15	0.15	0.10
283.85	0.00	0.00	0.00	0.00
289.04	0.01	0.00	0.00	0.00
291.08	15.03	15.02	15.04	13.13
291.19	0.05	0.05	0.05	0.07
300.01	3.16	3.17	3.16	2.21
307.60	0.00	0.00	0.00	0.00
309.07	0.01	0.01	0.01	0.00

Table S12 Calculated Raman intensities of the Γ-point vibrational modes of Sn_2S_3, using three different displacement steps along the eigenvectors, and also a denser **k**-point sampling mesh with $\Delta Q(V)$ = 0.01 Å, to compute the polarizability derivatives.

5. Convergence of the Γ-point spectral linewidths with respect to the lifetime-sampling mesh

The computational cost of post processing the third-order calculations to calculate the phonon lifetimes, spectral linewidths and lattice thermal conductivity scales unfavourably with the size of the reciprocal-space mesh used to evaluate the Brillouin-zone integrals.

The phonon linewidths of even “simple” binary compounds have been shown to vary non-smoothly with respect to phonon wavevector,[6] and it is therefore important explicitly to check convergence of these properties with respect to the Brillouin-zone sampling.

We therefore calculated the linewidths of the Γ-point phonon modes with systematically-increasing lifetime-sampling meshes (a subset of the post processing required to calculate the full thermal-conductivity tensors, and therefore a less computationally-demanding task). We also tested two different methods of interpolating between **q**-points during the Brillouin-zone integration, *viz.* the linear tetrahedron method and a Gaussian smearing scheme with a width, σ, of 0.1 THz. The tetrahedron method is, in principle, more accurate, but Gaussian smearing typically converges faster with respect to the size of the sampling mesh.

Figs. S4-S7 show the results of the tests performed on SnS_2, *Pnma* and π-cubic SnS, and Sn_2S_3. For all four compounds, the linewidths typically show oscillatory behaviour at smaller sampling meshes, which are then damped at larger mesh sizes. The speed of convergence is dependent on both the system and the specific phonon mode; whereas convergence is reached rapidly for the SnS_2 and π-cubic SnS linewidths, it is generally slower for modes in *Pnma* SnS and Sn_2S_3, which exhibit the largest linewidths among the four compounds. In particular, many of the modes in *Pnma* SnS display striking large-amplitude oscillations at smaller mesh sizes. Interestingly, this slow convergence does not seem to manifest in the calculated thermal-conductivity curves (see Section 8, Fig. S19), perhaps because in most cases the broad linewidths (short lifetimes) would mean that these modes would invariably make a negligible contribution to the overall thermal conductivity.

Despite the non-smooth convergence of the *Pnma* SnS and Sn_2S_3 linewidths, these tests do indicate that the mesh sizes used to generate the simulated spectra in Fig. 3 in the text are well converged, at least within the limitations of other technical parameters (e.g. the supercell size used to calculate the second- and third-order force constants).

We note that, for π-cubic SnS, we were only able to perform tests with the tetrahedron method. This is due to a performance optimisation in the Phono3py code,[6] which makes the Gaussian smearing considerably more expensive for systems with large numbers of phonon bands. However, for the other systems the two integration methods appear to give similar results, and, as noted above, the convergence of the linewidths with respect to mesh sampling appears to be relatively rapid in this system.

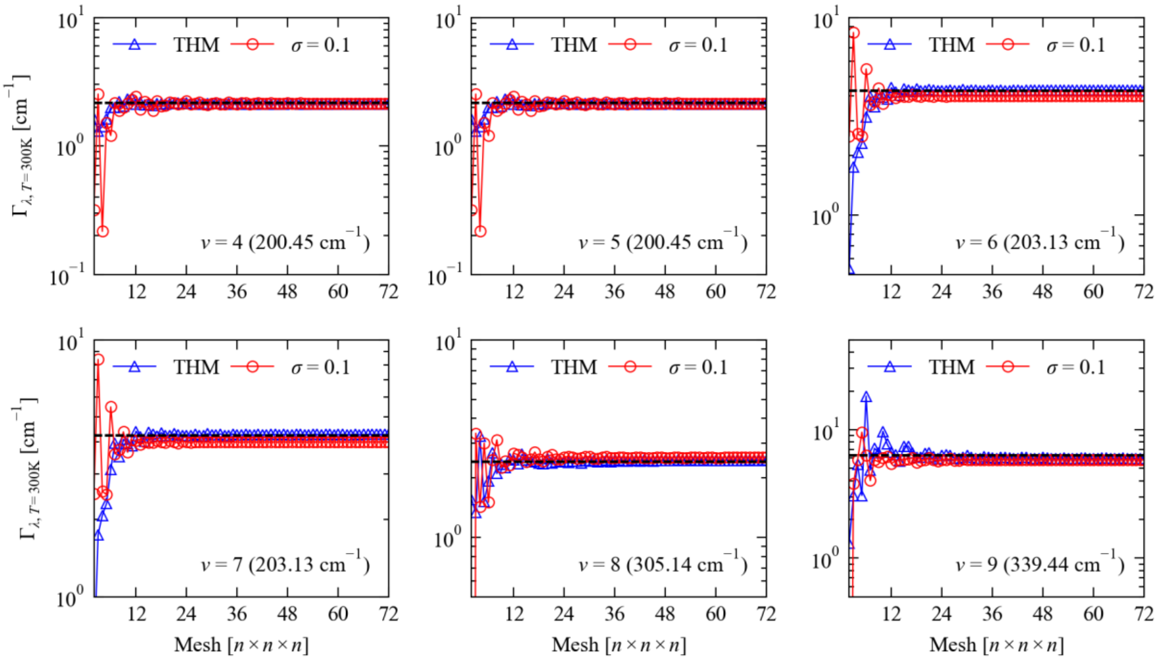

Figure S4 Convergence of the 300 K spectroscopic linewidths of the Γ-point optic modes of SnS_2 with the size of the lifetime-sampling **q**-point mesh, using the linear tetrahedron method (blue triangles) and a Gaussian broadening with a width, σ, of 0.1 THz (red circles) to integrate the Brillouin zone.

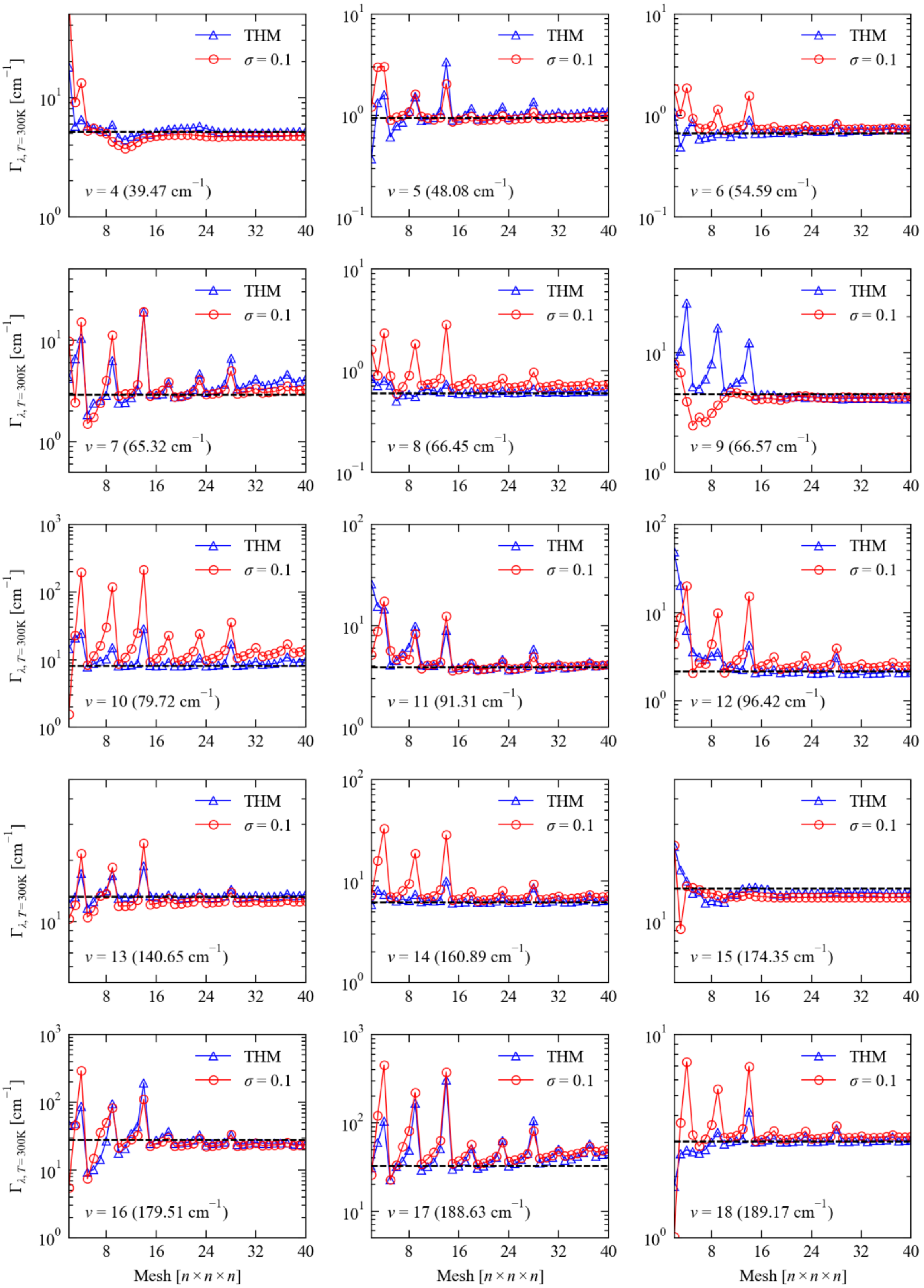

Figure S5 Convergence of the 300 K spectroscopic linewidths of the Γ-point optic modes of *Pnma* SnS with the size of the lifetime-sampling **q**-point mesh, using the linear tetrahedron method (blue triangles) and a Gaussian broadening with a width of 0.1 THz (red circles) to integrate the Brillouin zone.

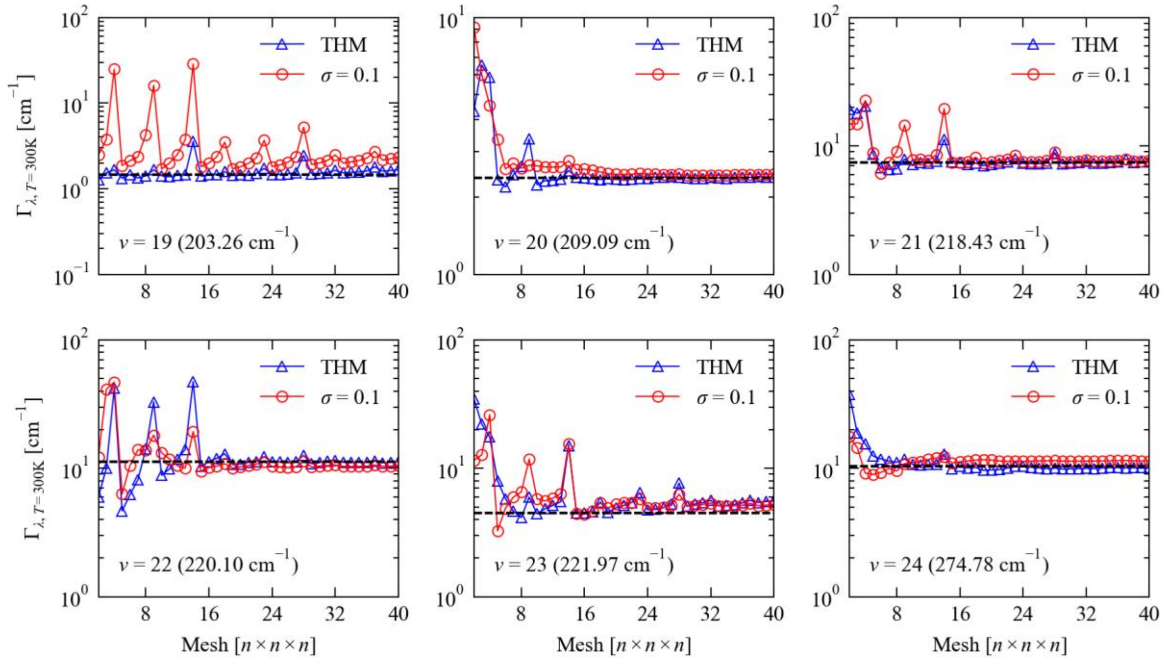

Figure S5 Convergence of the 300 K spectroscopic linewidths of the Γ-point optic modes of *Pnma* SnS with the size of the lifetime-sampling **q**-point mesh, using the linear tetrahedron method (blue triangles) and a Gaussian broadening with a width of 0.1 THz (red circles) to integrate the Brillouin zone.

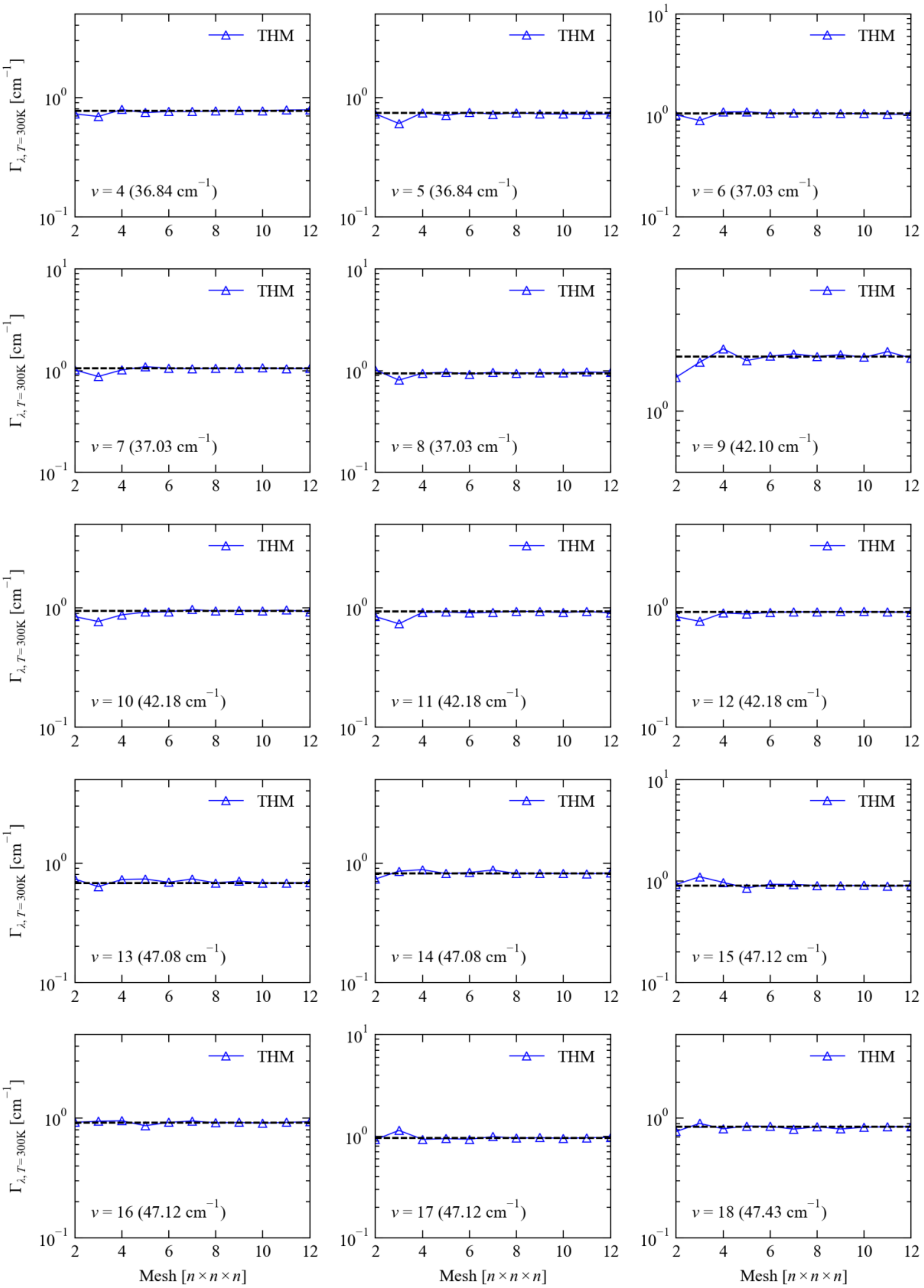

Figure S6 Convergence of the 300 K spectroscopic linewidths of the Γ-point optic modes of π-cubic SnS with the size of the lifetime-sampling **q**-point mesh, using the linear tetrahedron method to integrate the Brillouin zone.

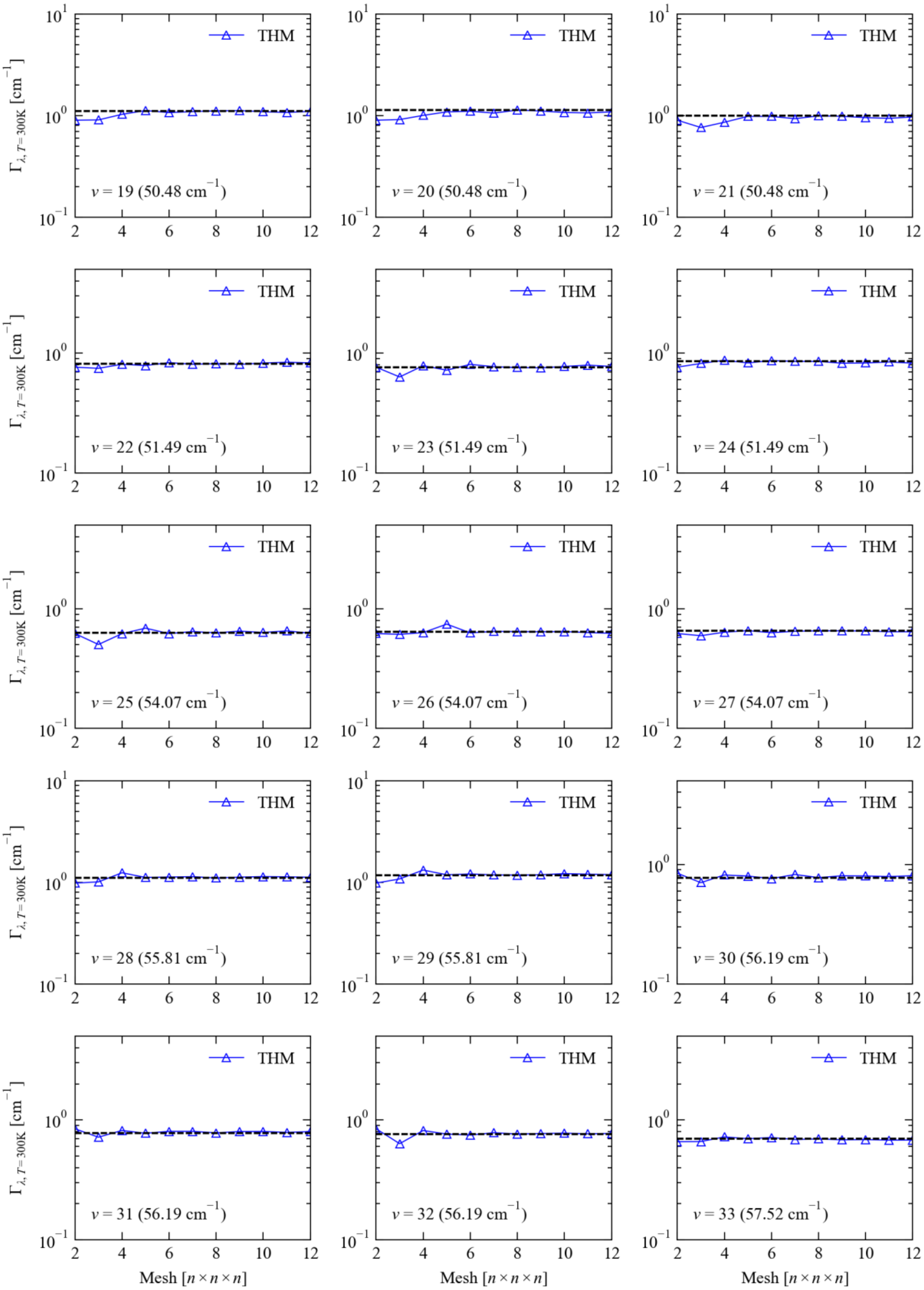

Figure S6 Convergence of the 300 K spectroscopic linewidths of the Γ-point optic modes of π-cubic SnS with the size of the lifetime-sampling **q**-point mesh, using the linear tetrahedron method to integrate the Brillouin zone.

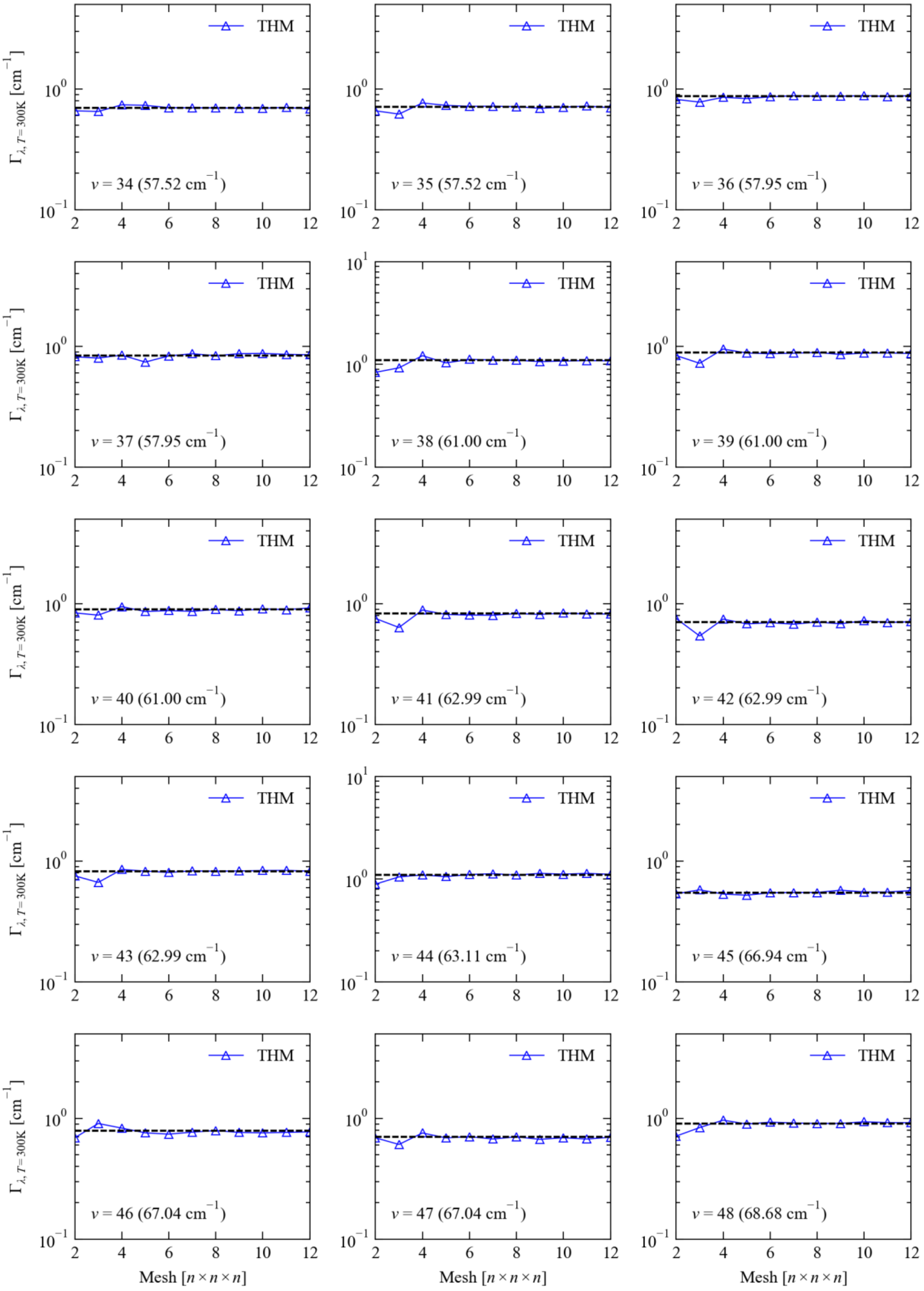

Figure S6 Convergence of the 300 K spectroscopic linewidths of the Γ-point optic modes of π-cubic SnS with the size of the lifetime-sampling **q**-point mesh, using the linear tetrahedron method to integrate the Brillouin zone.

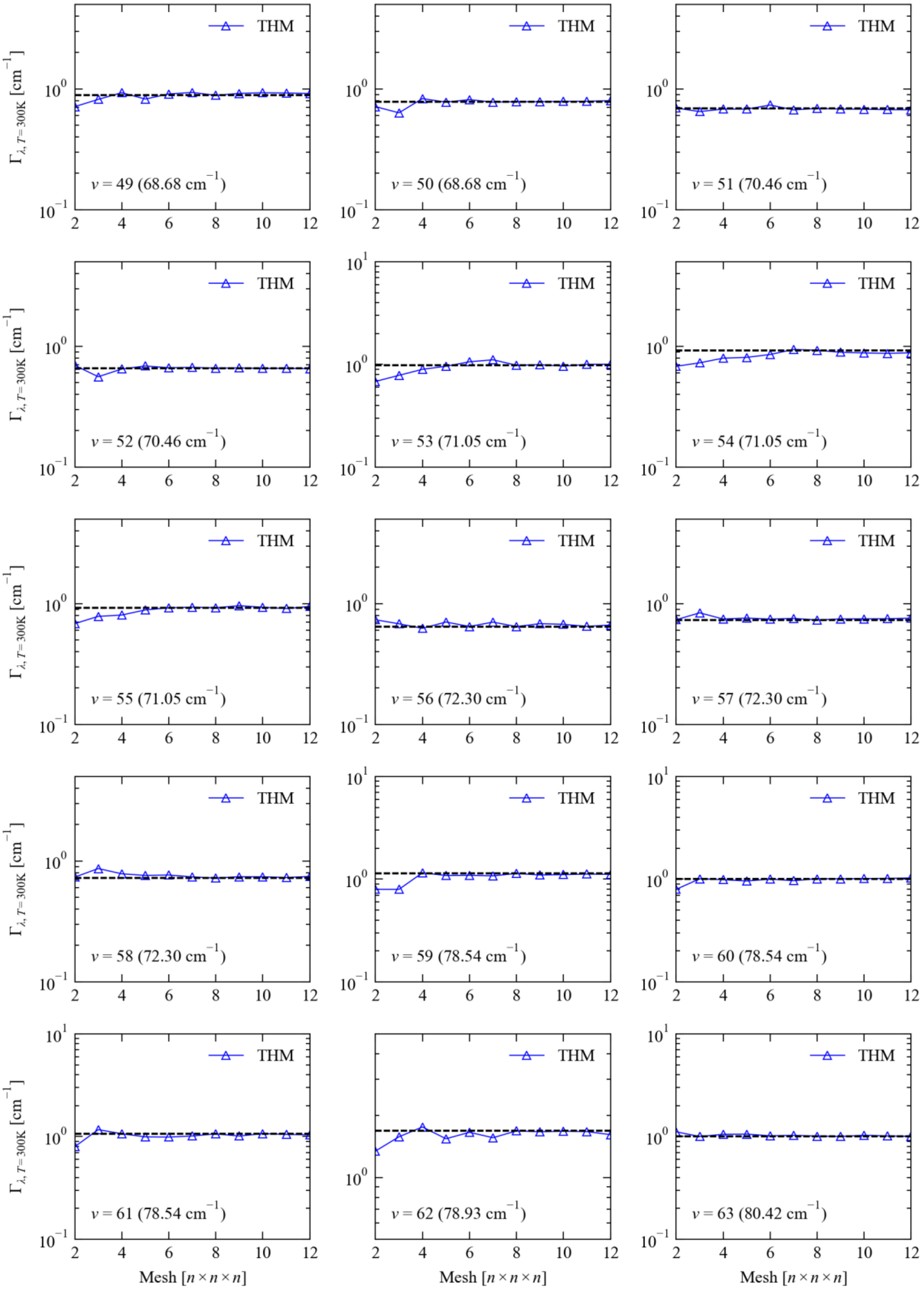

Figure S6 Convergence of the 300 K spectroscopic linewidths of the Γ-point optic modes of π-cubic SnS with the size of the lifetime-sampling **q**-point mesh, using the linear tetrahedron method to integrate the Brillouin zone.

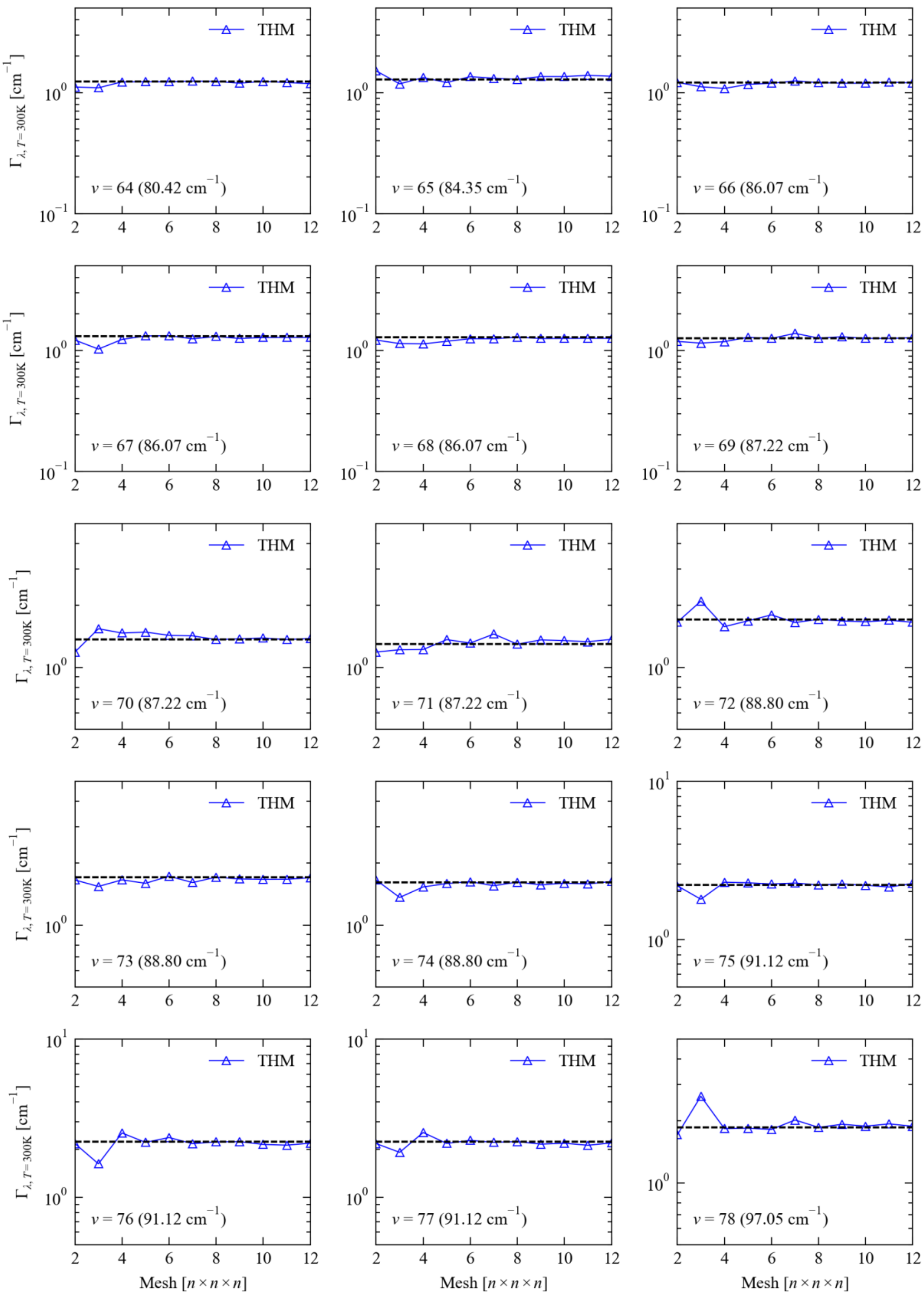

Figure S6 Convergence of the 300 K spectroscopic linewidths of the Γ-point optic modes of π-cubic SnS with the size of the lifetime-sampling **q**-point mesh, using the linear tetrahedron method to integrate the Brillouin zone.

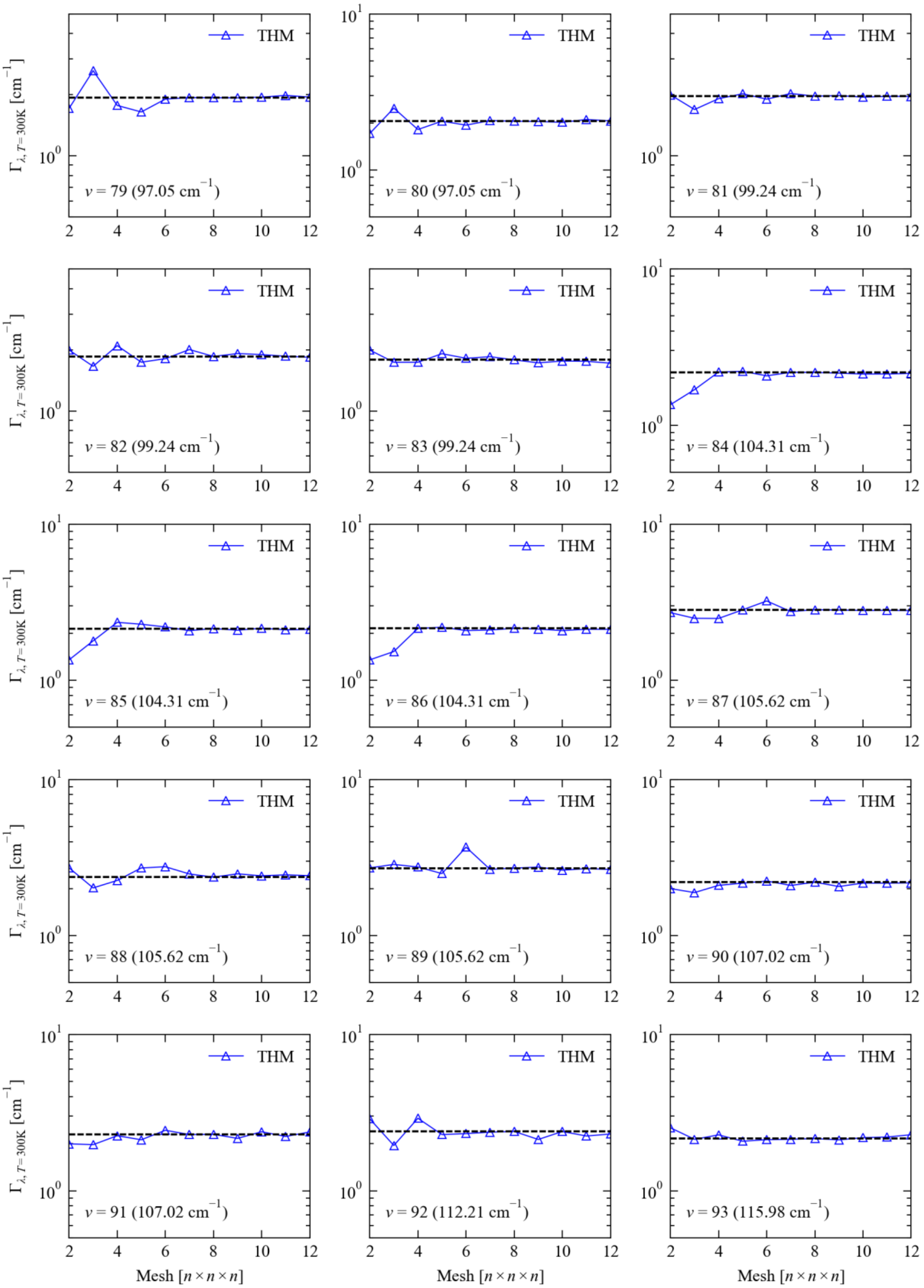

Figure S6 Convergence of the 300 K spectroscopic linewidths of the Γ-point optic modes of π-cubic SnS with the size of the lifetime-sampling **q**-point mesh, using the linear tetrahedron method to integrate the Brillouin zone.

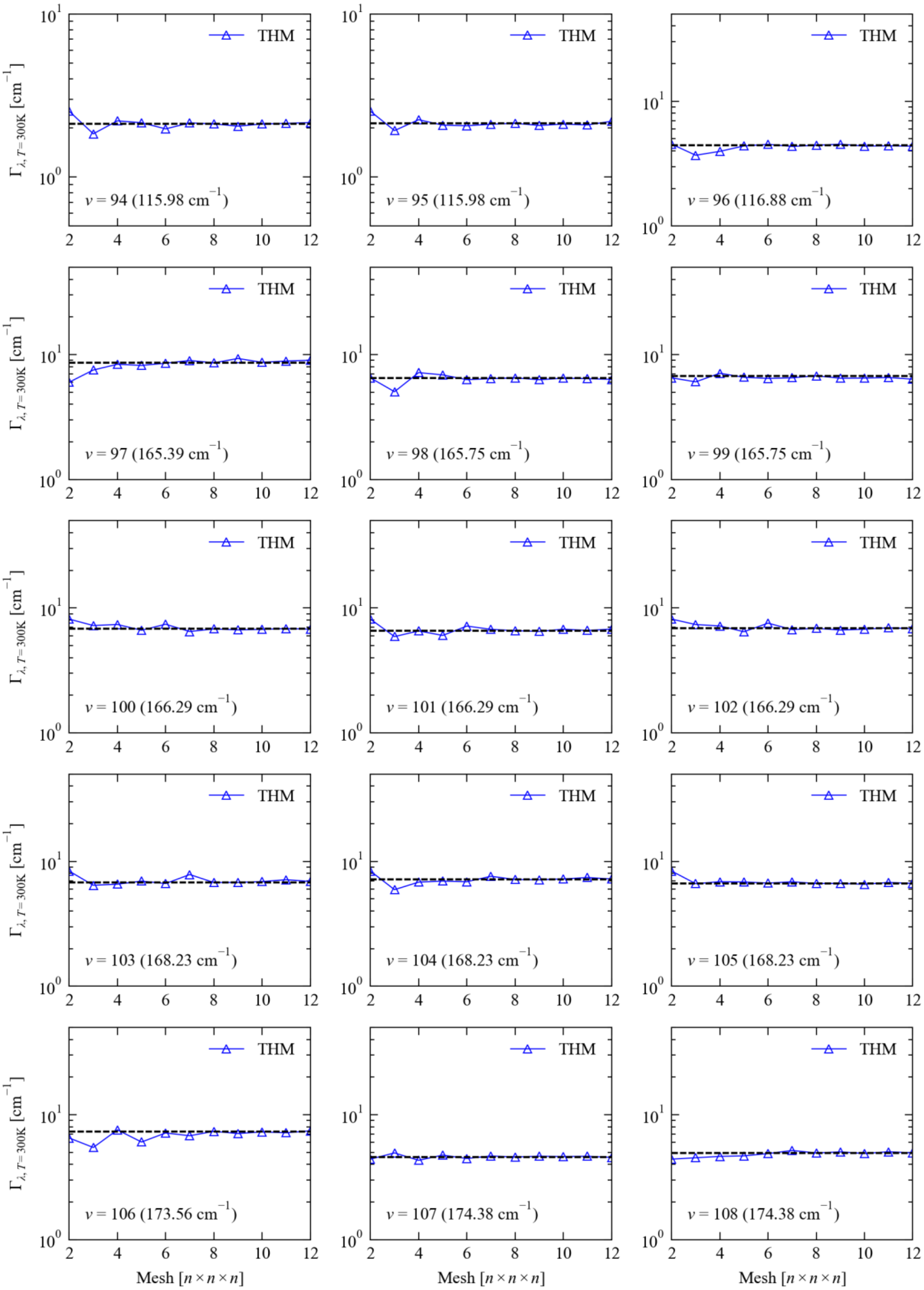

Figure S6 Convergence of the 300 K spectroscopic linewidths of the Γ-point optic modes of π-cubic SnS with the size of the lifetime-sampling **q**-point mesh, using the linear tetrahedron method to integrate the Brillouin zone.

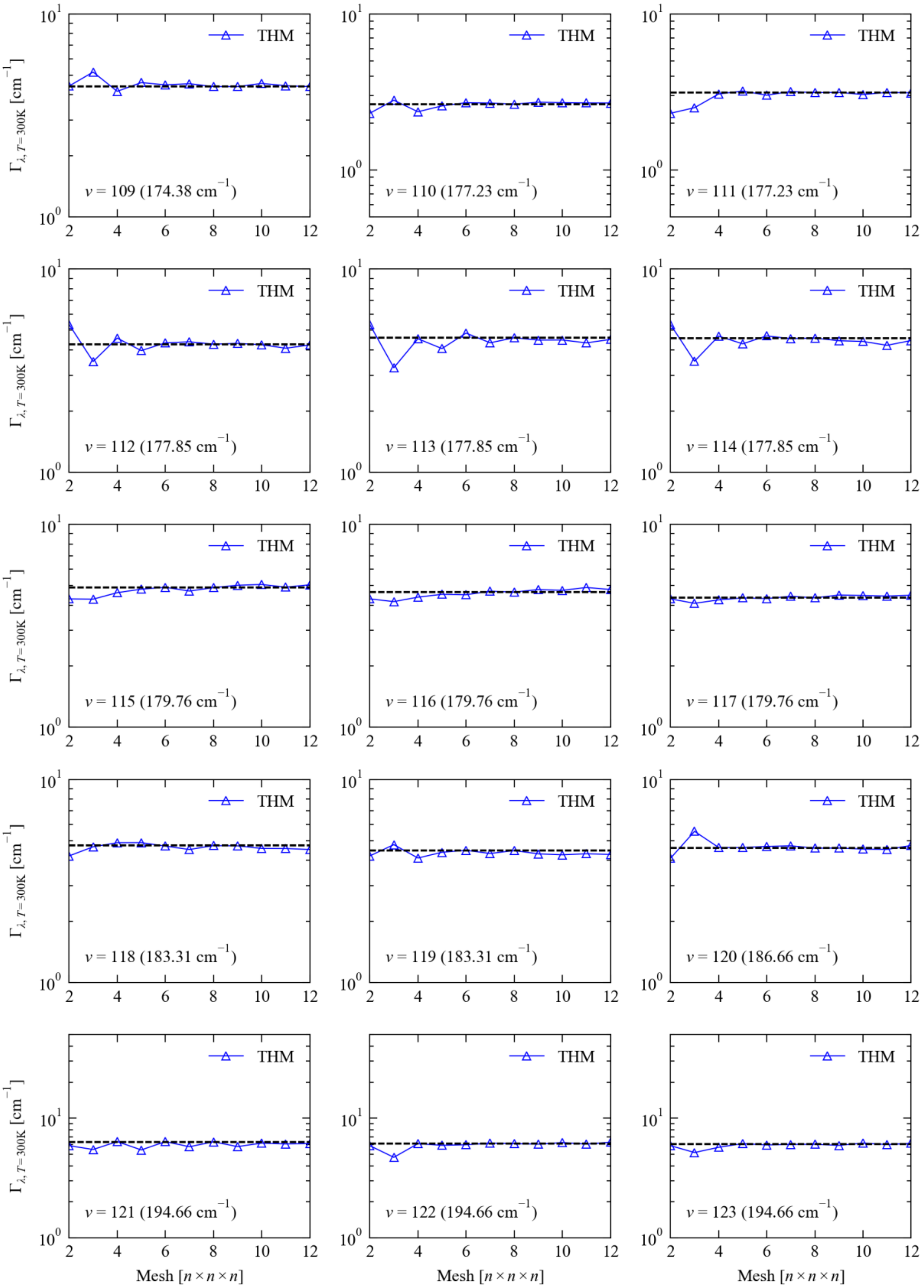

Figure S6 Convergence of the 300 K spectroscopic linewidths of the Γ-point optic modes of π-cubic SnS with the size of the lifetime-sampling **q**-point mesh, using the linear tetrahedron method to integrate the Brillouin zone.

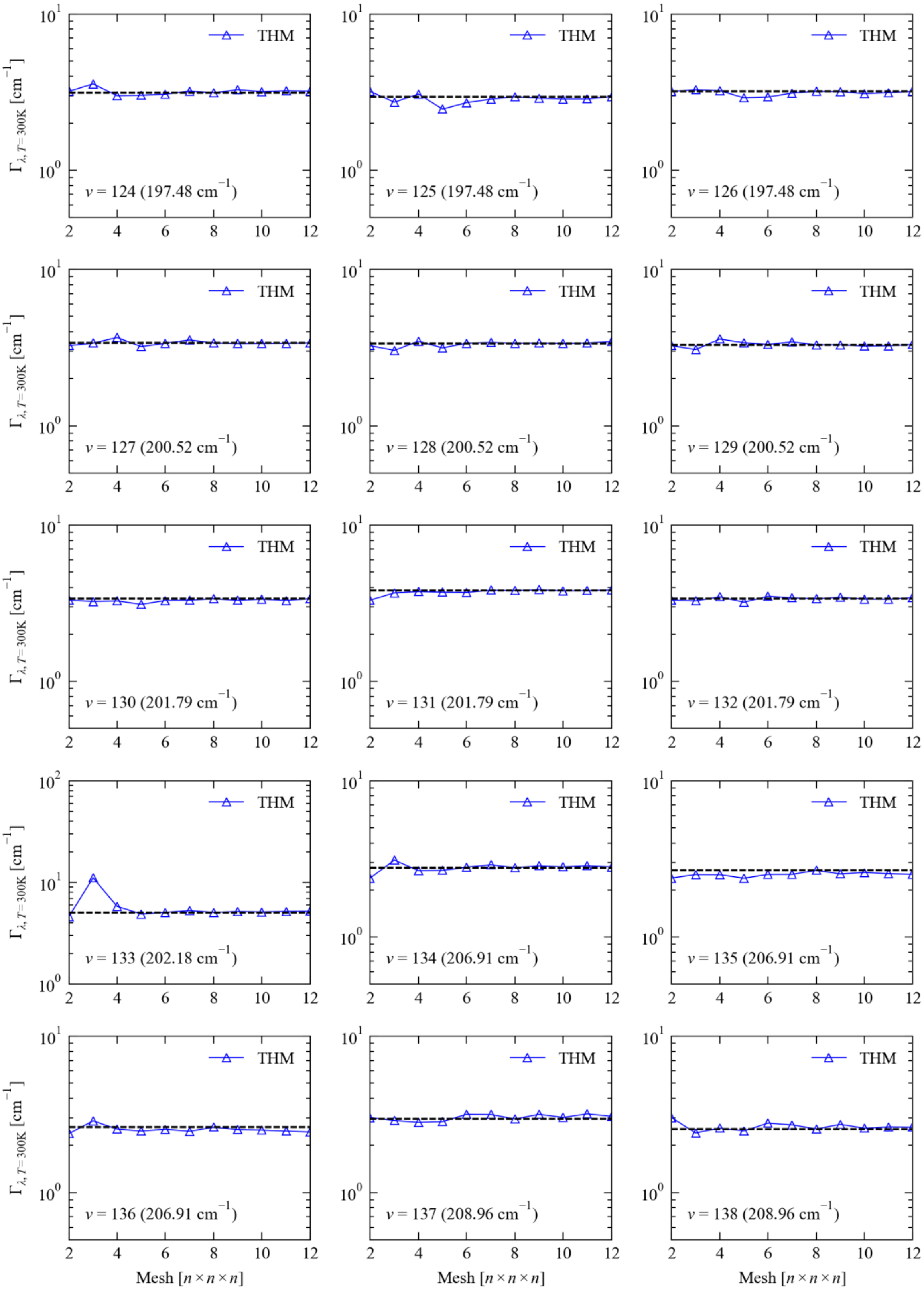

Figure S6 Convergence of the 300 K spectroscopic linewidths of the Γ-point optic modes of π-cubic SnS with the size of the lifetime-sampling **q**-point mesh, using the linear tetrahedron method to integrate the Brillouin zone.

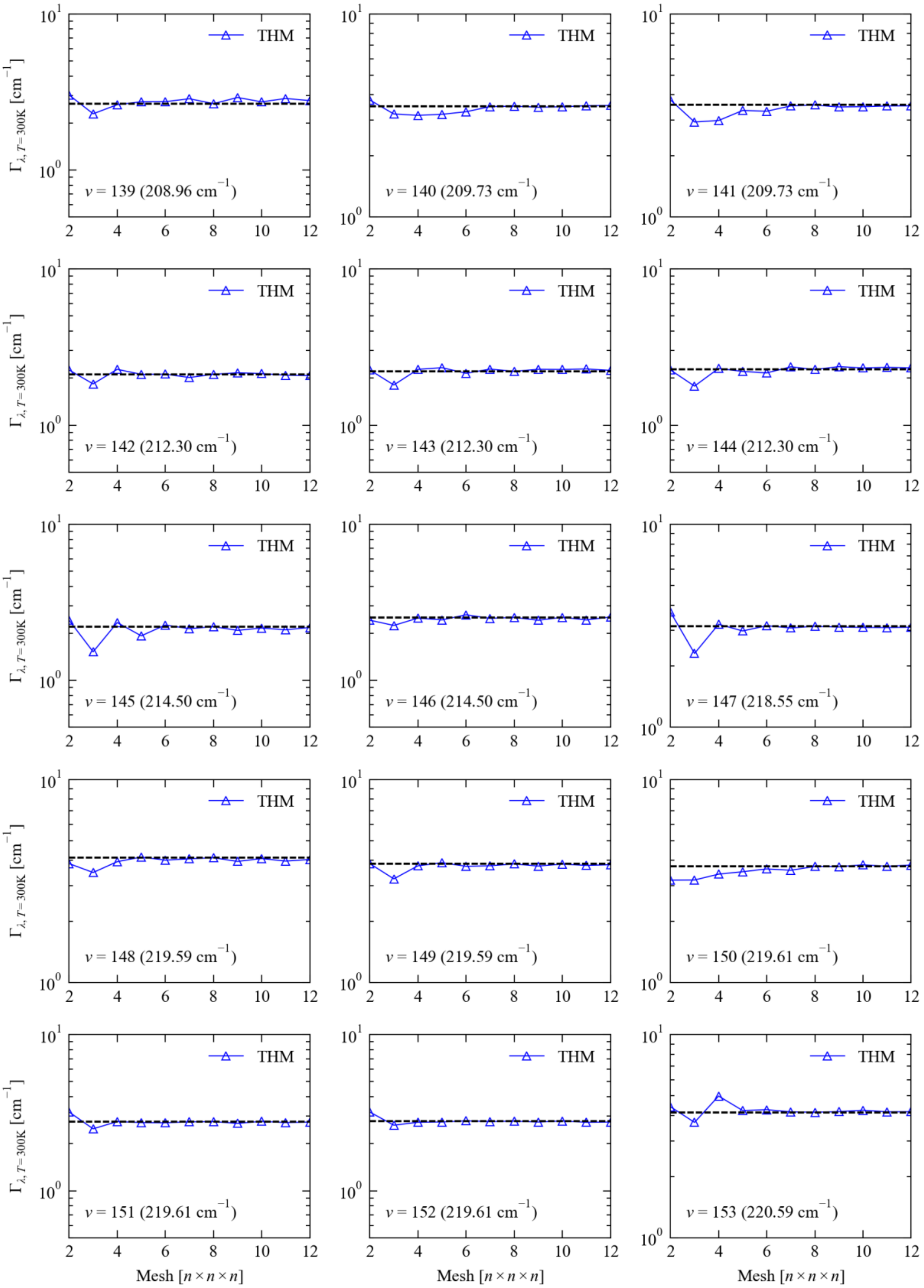

Figure S6 Convergence of the 300 K spectroscopic linewidths of the Γ-point optic modes of π-cubic SnS with the size of the lifetime-sampling **q**-point mesh, using the linear tetrahedron method to integrate the Brillouin zone.

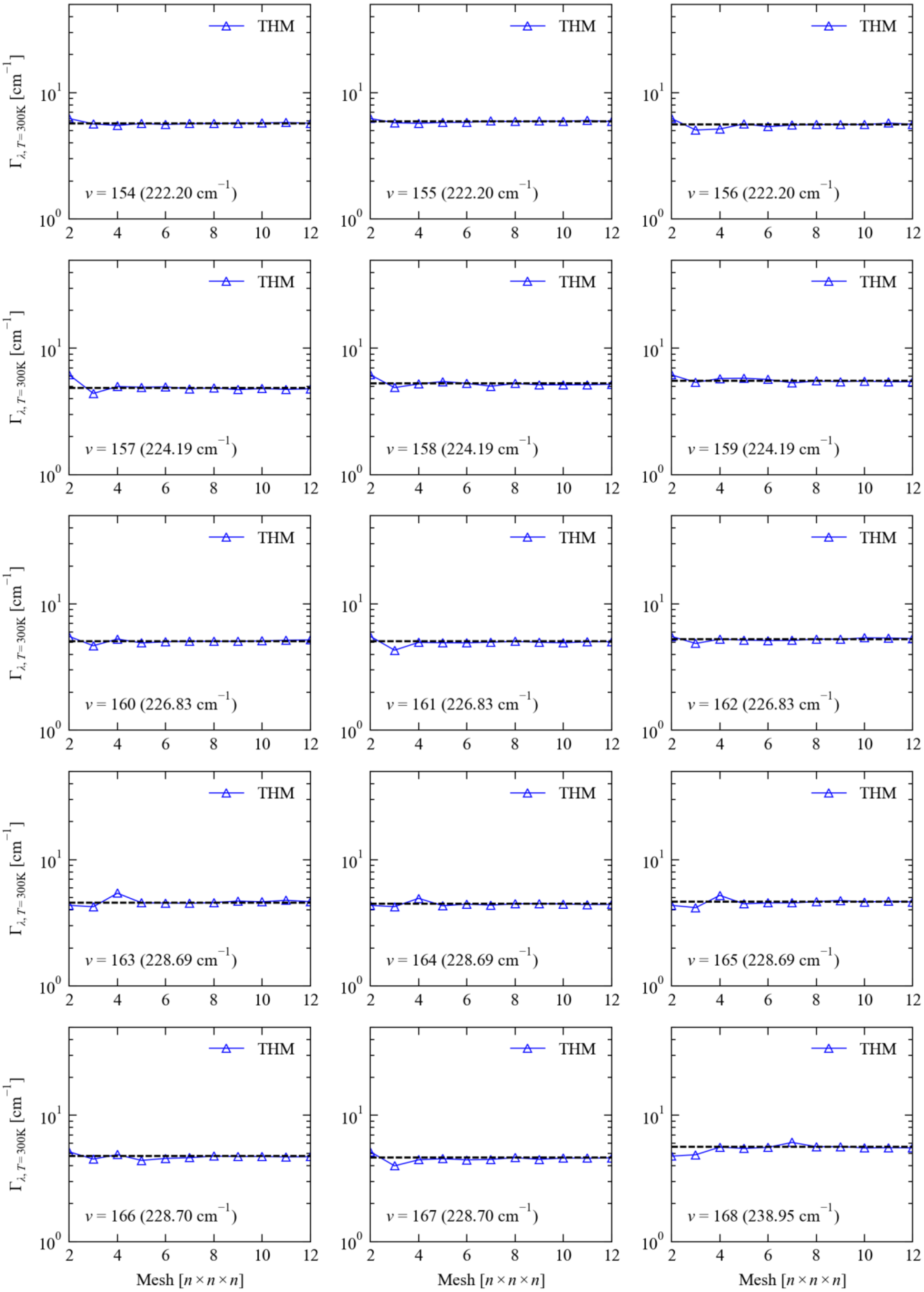

Figure S6 Convergence of the 300 K spectroscopic linewidths of the Γ-point optic modes of π-cubic SnS with the size of the lifetime-sampling **q**-point mesh, using the linear tetrahedron method to integrate the Brillouin zone.

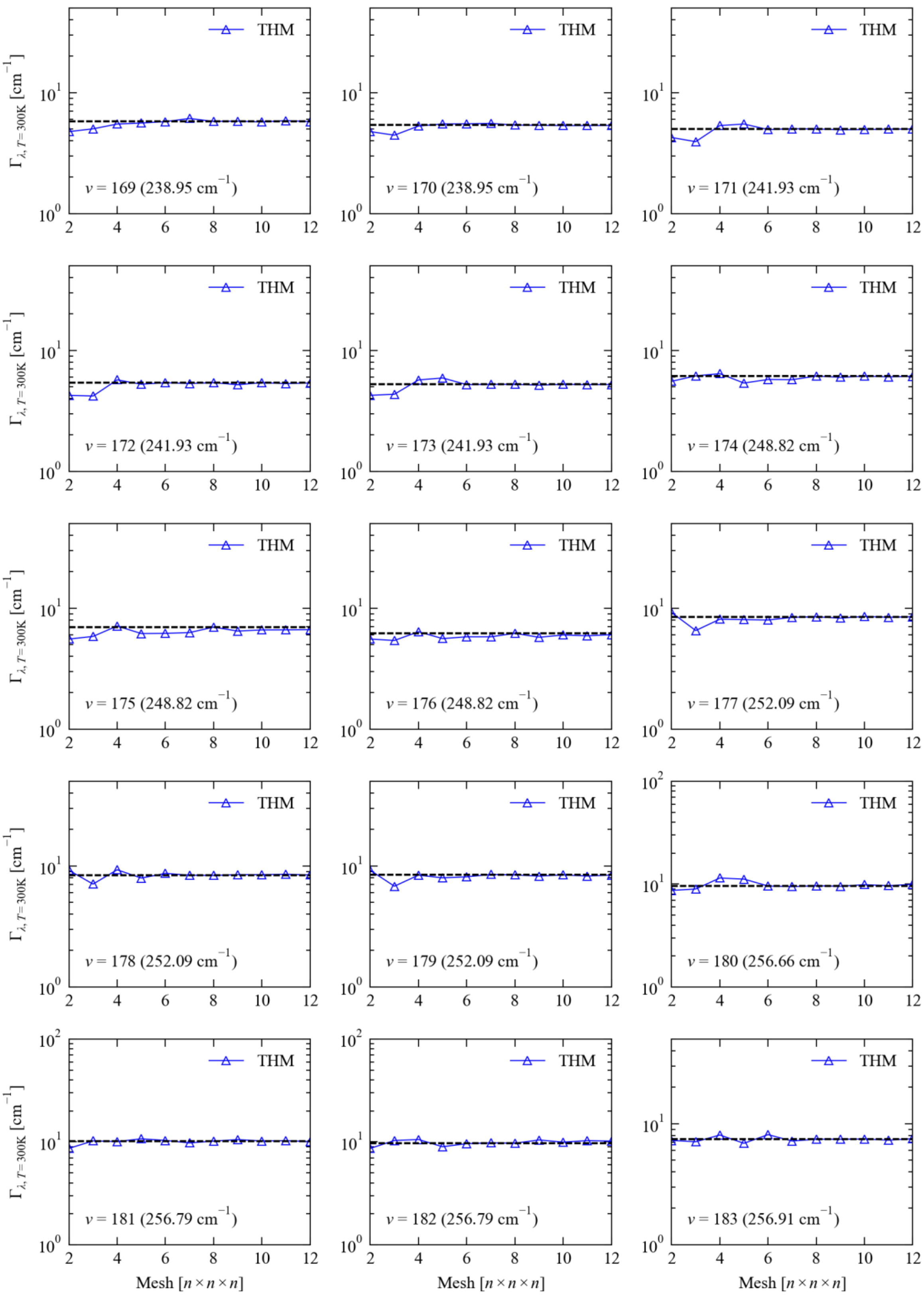

Figure S6 Convergence of the 300 K spectroscopic linewidths of the Γ-point optic modes of π-cubic SnS with the size of the lifetime-sampling **q**-point mesh, using the linear tetrahedron method to integrate the Brillouin zone.

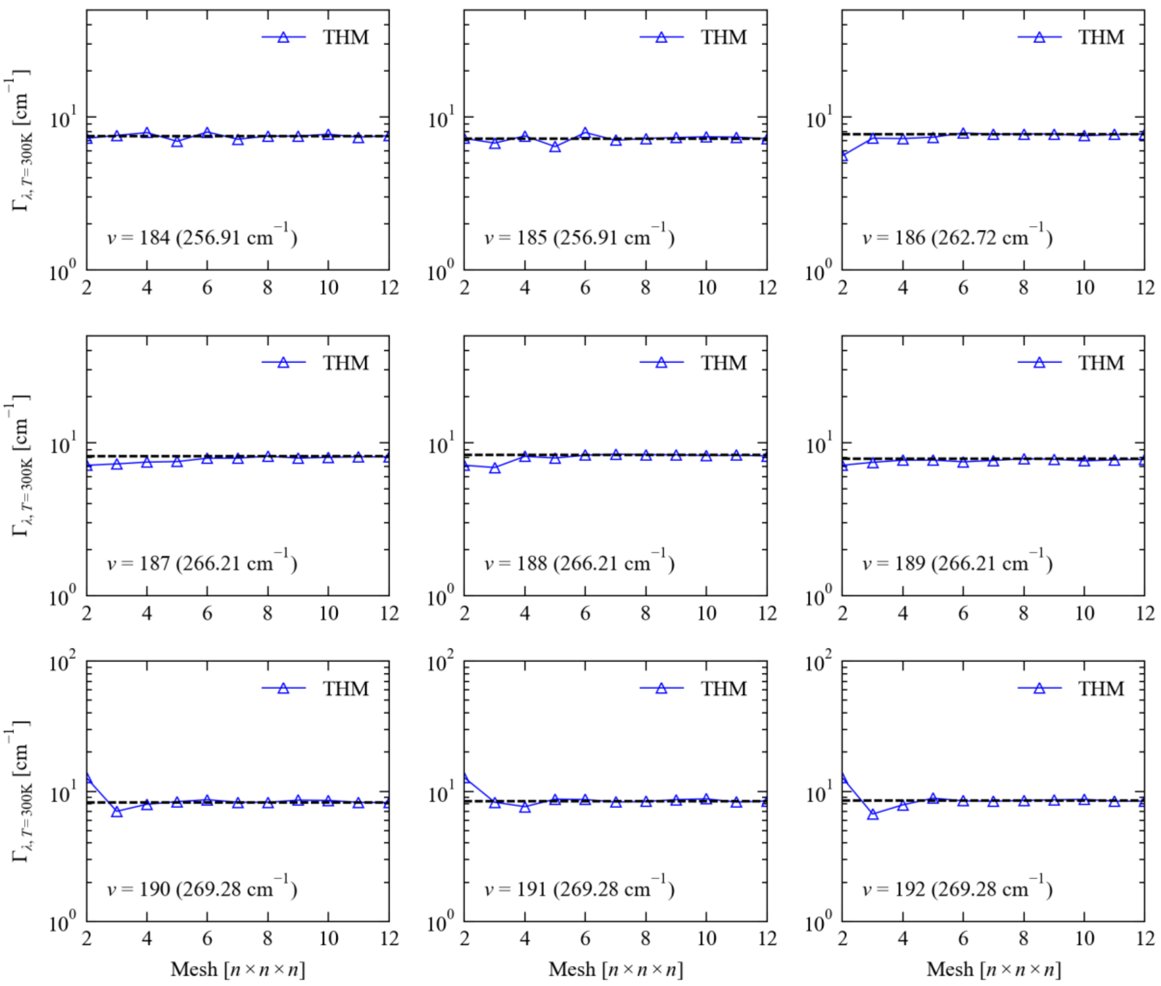

Figure S6 Convergence of the 300 K spectroscopic linewidths of the Γ-point optic modes of π-cubic SnS with the size of the lifetime-sampling **q**-point mesh, using the linear tetrahedron method to integrate the Brillouin zone.

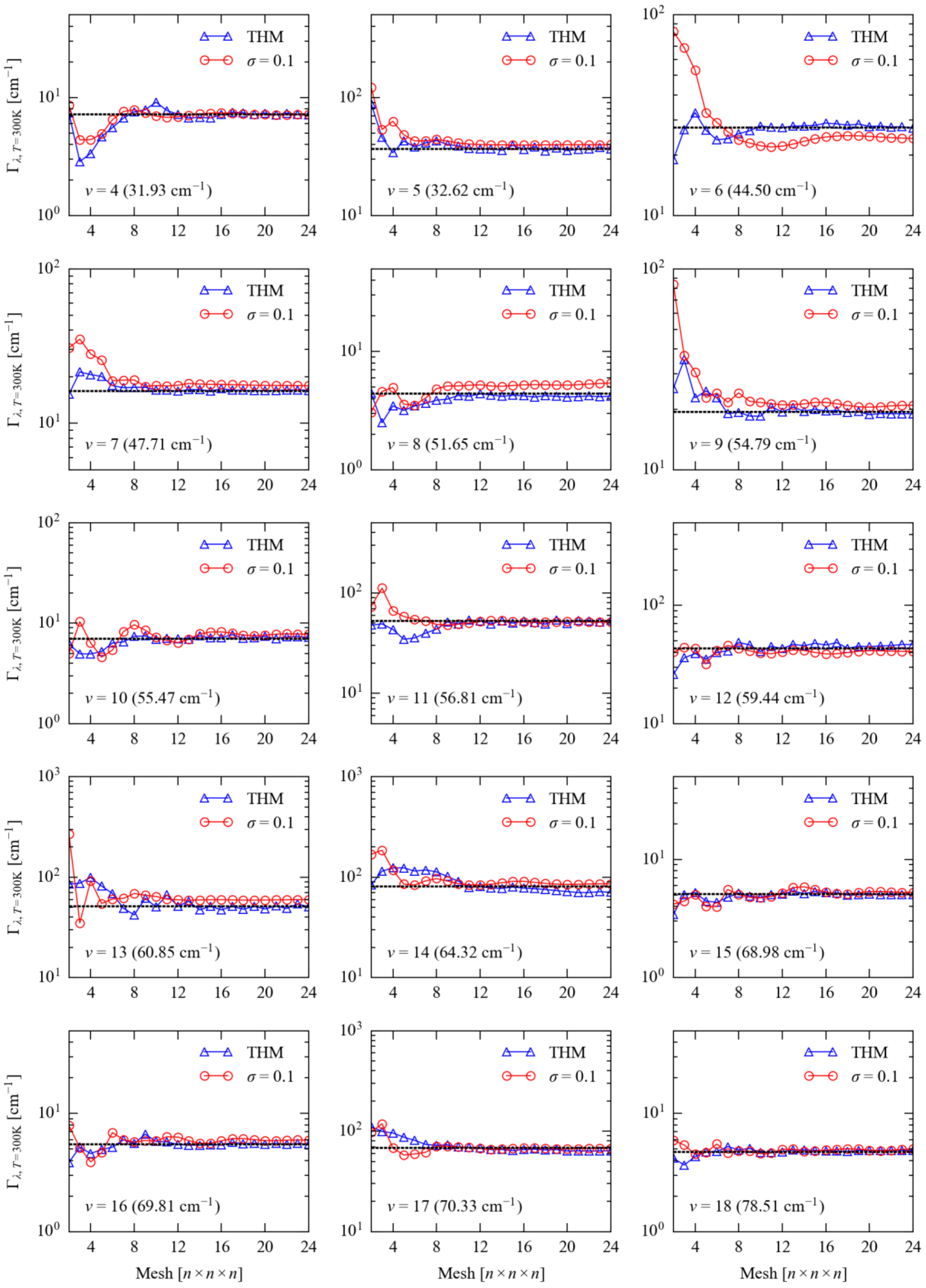

Figure S7 Convergence of the 300 K spectroscopic linewidths of the Γ-point optic modes of Sn_2S_3 with the size of the lifetime-sampling **q**-point mesh, using the linear tetrahedron method (blue triangles) and a Gaussian broadening with a width of 0.1 THz (red circles) to integrate the Brillouin zone.

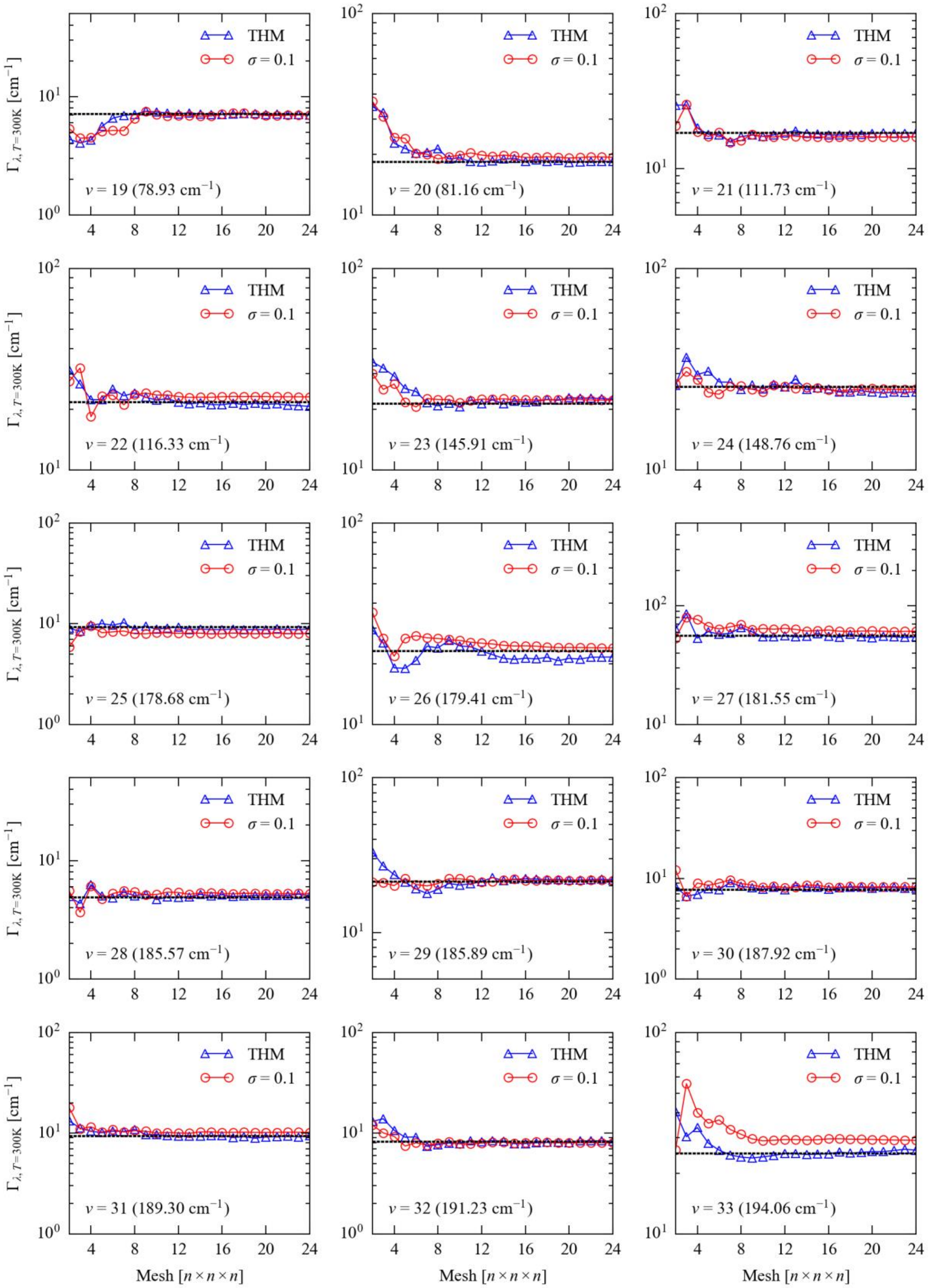

Figure S7 Convergence of the 300 K spectroscopic linewidths of the Γ-point optic modes of Sn_2S_3 with the size of the lifetime-sampling **q**-point mesh, using the linear tetrahedron method (blue triangles) and a Gaussian broadening with a width of 0.1 THz (red circles) to integrate the Brillouin zone.

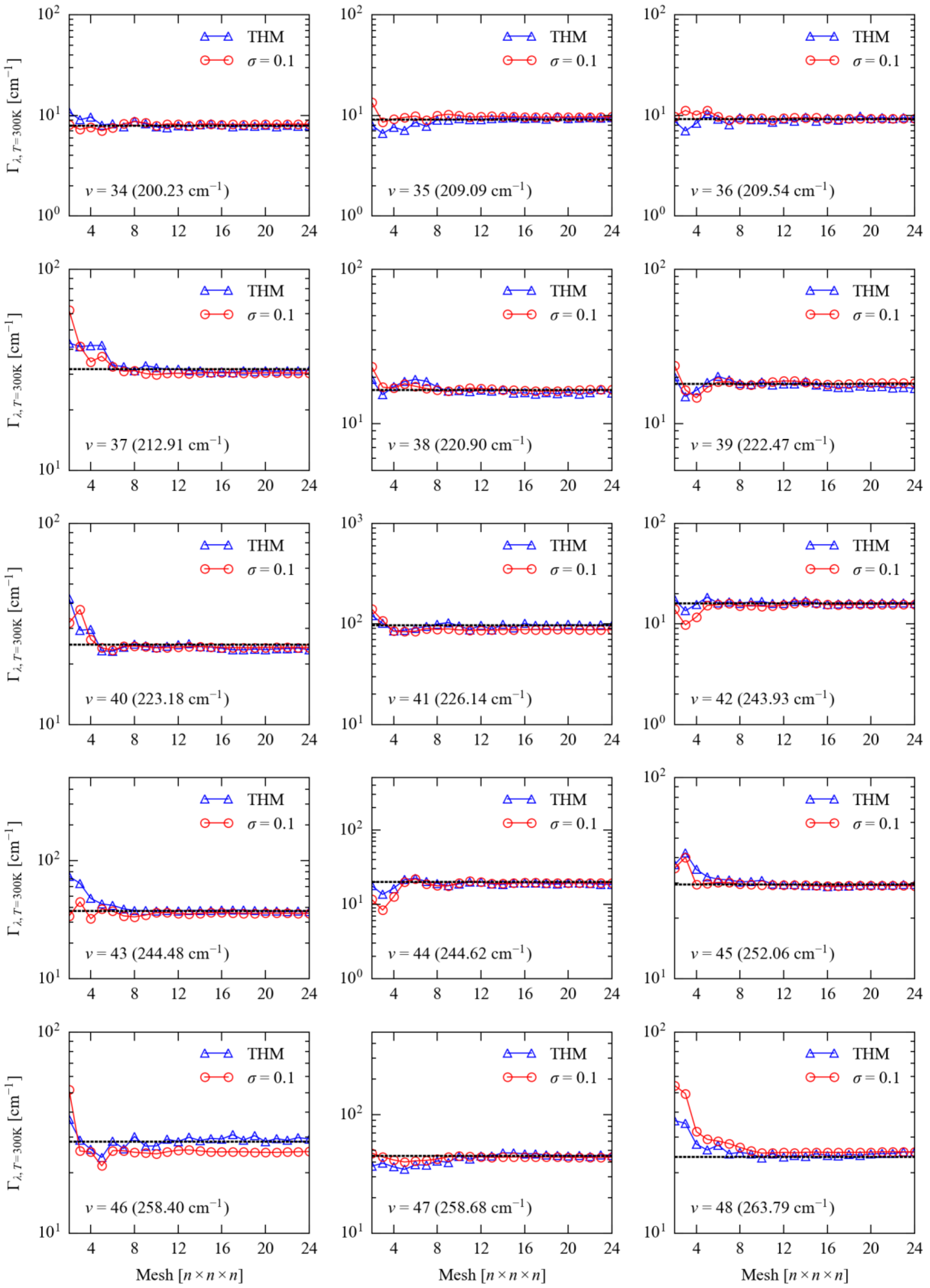

Figure S7 Convergence of the 300 K spectroscopic linewidths of the Γ-point optic modes of Sn_2S_3 with the size of the lifetime-sampling **q**-point mesh, using the linear tetrahedron method (blue triangles) and a Gaussian broadening with a width of 0.1 THz (red circles) to integrate the Brillouin zone.

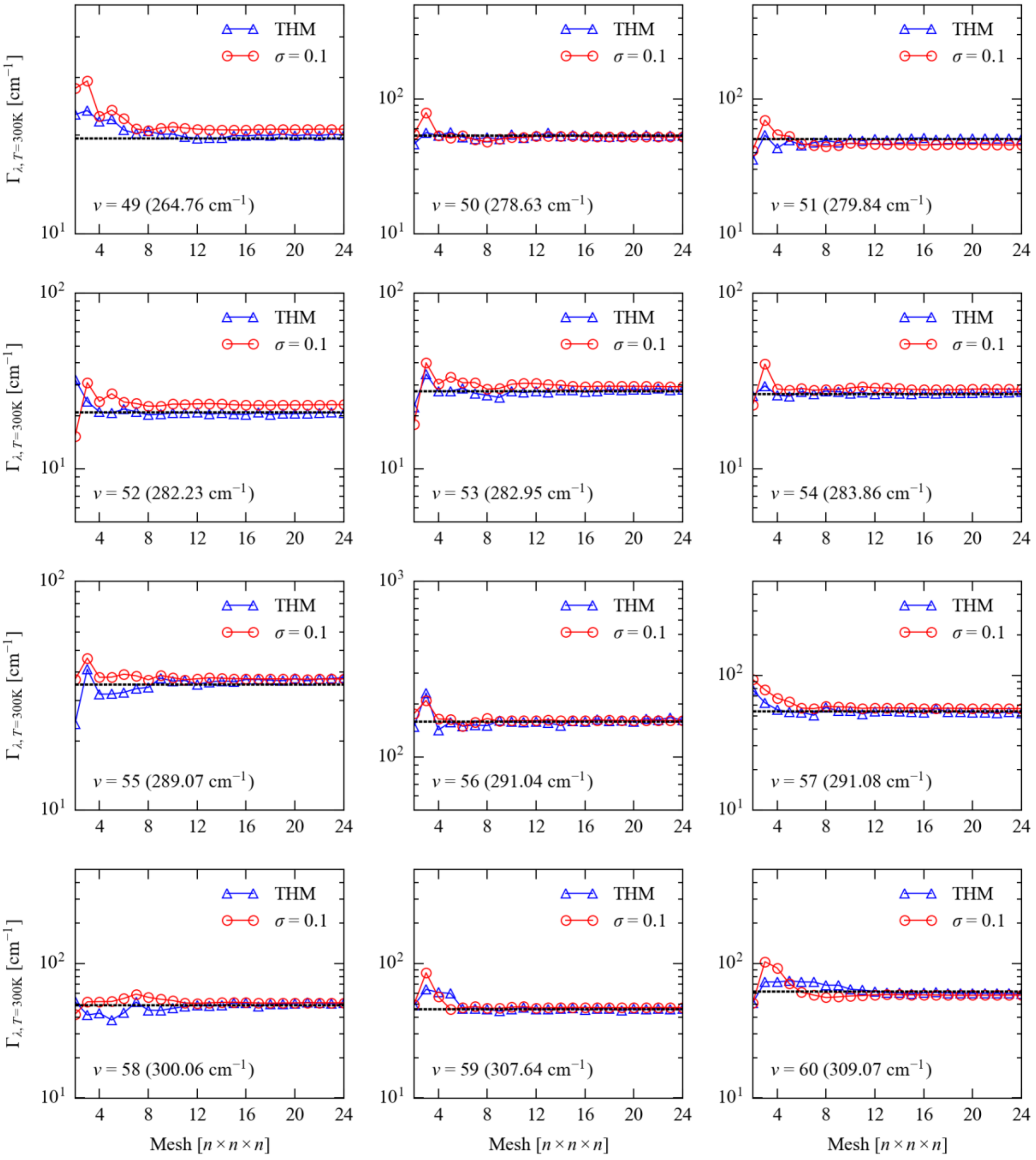

Figure S7 Convergence of the 300 K spectroscopic linewidths of the Γ-point optic modes of Sn_2S_3 with the size of the lifetime-sampling **q**-point mesh, using the linear tetrahedron method (blue triangles) and a Gaussian broadening with a width of 0.1 THz (red circles) to integrate the Brillouin zone.

6. Axial anisotropy in the lattice thermal conductivity of SnS_2, *Pnma* SnS and Sn_2S_3

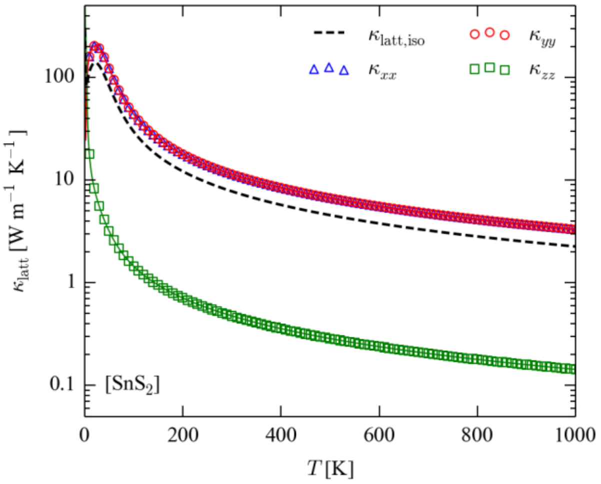

Figure S8 Axial anisotropy in the lattice thermal conductivity ($\boldsymbol{\kappa}_{\mathrm{latt}}$) of SnS_2. The plot shows the three diagonal components of the $\boldsymbol{\kappa}_{\mathrm{latt}}$ tensor as a function of temperature (κ_{xx} - blue triangles, κ_{yy} - red circles, κ_{zz} - green squares), plus the isotropic average plotted in Fig. 5 in the text ($\kappa_{\mathrm{latt,iso}}$, dashed black line).

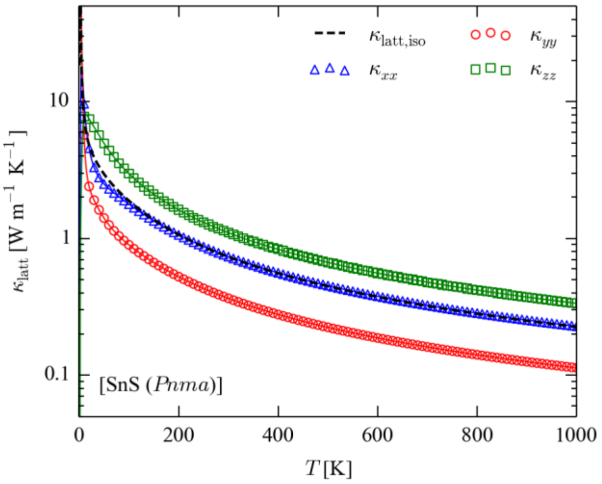

Figure S9 Axial anisotropy in the lattice thermal conductivity ($\boldsymbol{\kappa}_{\mathrm{latt}}$) of *Pnma* SnS. The plot shows the three diagonal components of the $\boldsymbol{\kappa}_{\mathrm{latt}}$ tensor as a function of temperature (κ_{xx} - blue triangles, κ_{yy} - red circles, κ_{zz} - green squares), plus the isotropic average plotted in Fig. 5 in the text ($\kappa_{\mathrm{latt,iso}}$, dashed black line).

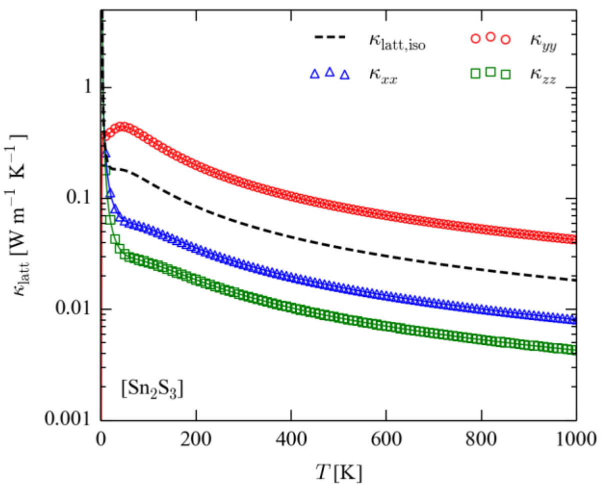

Figure S10 Axial anisotropy in the lattice thermal conductivity ($\boldsymbol{\kappa}_{\mathrm{latt}}$) of Sn_2S_3. The plot shows the three diagonal components of the $\boldsymbol{\kappa}_{\mathrm{latt}}$ tensor as a function of temperature (κ_{xx} - blue triangles, κ_{yy} - red circles, κ_{zz} - green squares), plus the isotropic average plotted in Fig. 5 in the text ($\kappa_{\mathrm{latt,iso}}$, dashed black line).

7. Effect of mass variance on the calculated thermal conductivities

	$\kappa_{\mathrm{latt,300K}}$ [W m^{-1} K^{-1}]							
	RTA				RTA + Isotope			
Compound	κ_{xx}	κ_{yy}	κ_{zz}	κ_{iso}	κ_{xx}	κ_{yy}	κ_{zz}	κ_{iso}
SnS_2	11.40	11.40	0.48	7.76	11.17	11.17	0.47	7.60
SnS (*Pnma*)	0.74	0.36	1.10	0.73	0.73	0.36	1.09	0.72
SnS (π-cubic)	-	-	-	0.13	-	-	-	0.13
Sn_2S_3	0.03	0.14	0.01	0.06	0.03	0.14	0.01	0.06
SnSe (*Pnma*)[a]	1.44	0.53	1.88	1.28	1.40	0.50	1.83	1.24
Cu_2ZnSnS_4[b]	1.75	1.75	1.57	1.69	1.65	1.65	1.50	1.60
$Cu_2ZnSnSe_4$[b]	4.68	4.68	3.98	4.44	4.59	4.59	3.89	4.36

Table S13 Room-temperature lattice thermal conductivities (κ_{latt}) of SnS_2, *Pnma* and π-cubic SnS and Sn_2S_3, calculated within the relaxation-time approximation (RTA) with and without isotope effects. The diagonal components of the thermal-conductivity tensors are given alongside the isotropic average $\kappa_{\mathrm{iso}} = (\kappa_{xx} + \kappa_{yy} + \kappa_{zz})/3$. The thermal conductivities of *Pnma* SnSe and Cu_2ZnSnS_4 and $Cu_2ZnSnSe_4$ (CZTS/Se), computed with and without isotope effects, are also given for comparison. [a]RTA data on SnSe from Ref. [7]. [b]RTA data on CZTS/Se from Ref. [8].

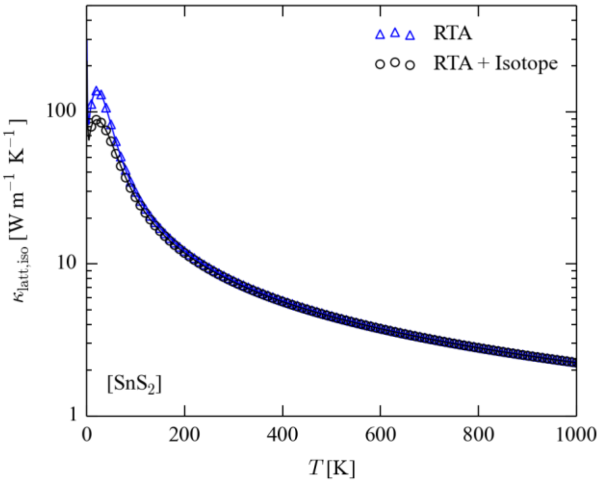

Figure S11 Isotropically-averaged lattice thermal conductivity (κ_{latt}) of SnS_2 as a function of temperature, calculated within the relaxation-time approximation (RTA) with (blue triangles) and without (black circles) isotope effects.

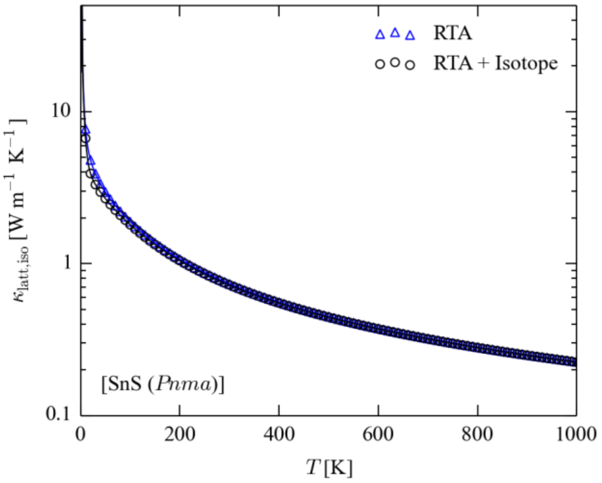

Figure S12 Isotropically-averaged lattice thermal conductivity (κ_{latt}) of *Pnma* SnS as a function of temperature, calculated within the relaxation-time approximation (RTA) with (blue triangles) and without (black circles) isotope effects.

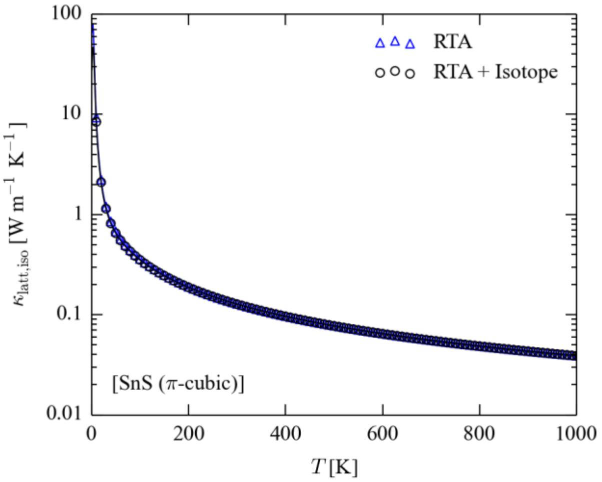

Figure S13 Isotropically-averaged lattice thermal conductivity (κ_{latt}) of π-cubic SnS as a function of temperature, calculated within the relaxation-time approximation (RTA) with (blue triangles) and without (black circles) isotope effects.

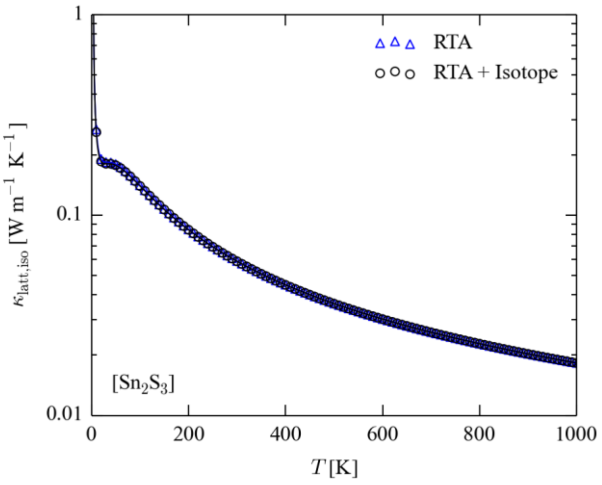

Figure S14 Isotropically-averaged lattice thermal conductivity (κ_{latt}) of Sn_2S_3 as a function of temperature, calculated within the relaxation-time approximation (RTA) with (blue triangles) and without (black circles) isotope effects.

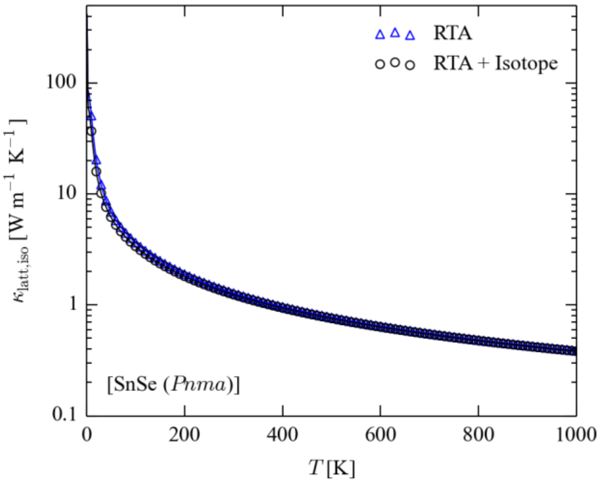

Figure S15 Isotropically-averaged lattice thermal conductivity (κ_{latt}) of *Pnma* SnSe as a function of temperature, calculated within the relaxation-time approximation (RTA) with (blue triangles) and without (black circles) isotope effects. The RTA data was taken from Ref. [7].

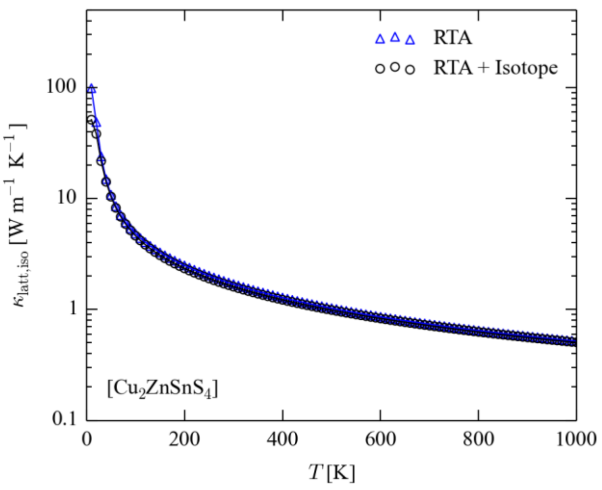

Figure S16 Isotropically-averaged lattice thermal conductivity (κ_{latt}) of Cu_2ZnSnS_4 (CZTS) as a function of temperature, calculated within the relaxation-time approximation (RTA) with (blue triangles) and without (black circles) isotope effects. The RTA data was taken from Ref. [8].

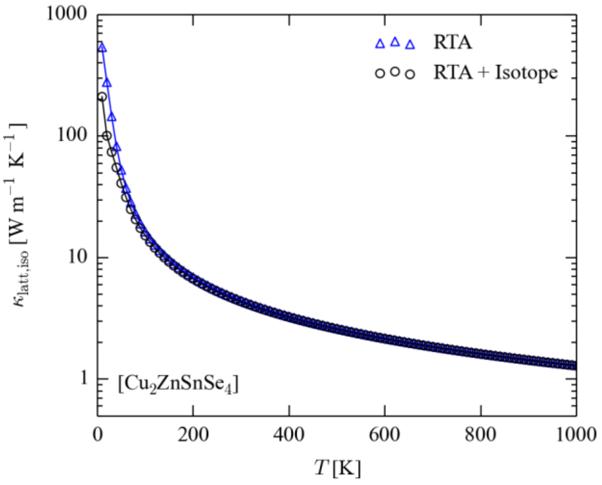

Figure S17 Isotropically-averaged lattice thermal conductivity (κ_{latt}) of $Cu_2ZnSnSe_4$ (CZTSe) as a function of temperature, calculated within the relaxation-time approximation (RTA) with (blue triangles) and without (black circles) isotope effects. The RTA data was taken from Ref. [8].

8. Convergence of the lattice thermal conductivity with respect to the lifetime-sampling mesh

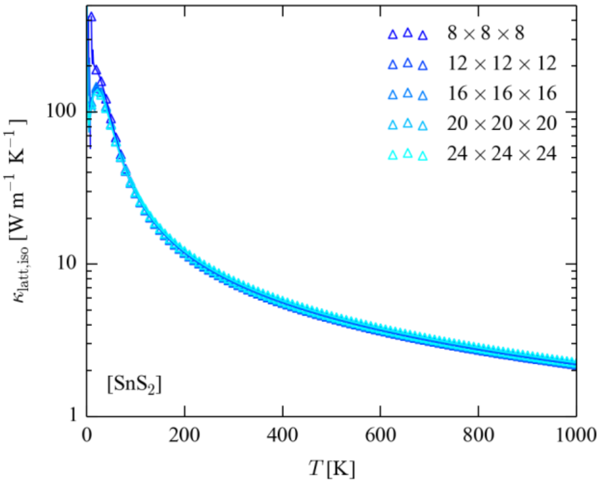

Figure S18 Isotropically-averaged lattice thermal conductivity (κ_{latt}) of SnS_2 computed with various lifetime-sampling **q**-point meshes.

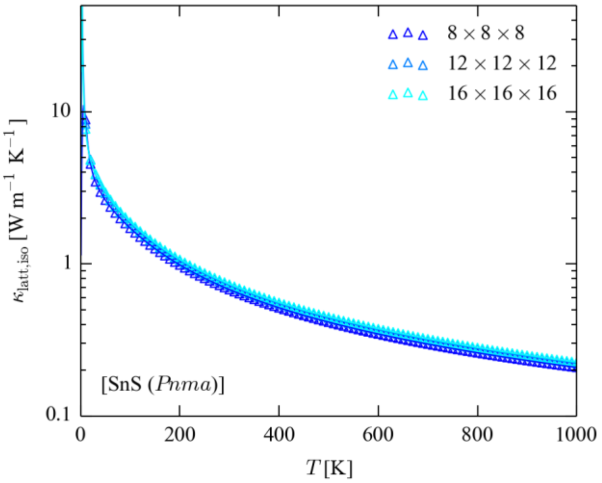

Figure S19 Isotropically-averaged lattice thermal conductivity (κ_{latt}) of *Pnma* SnS computed with various lifetime-sampling **q**-point meshes.

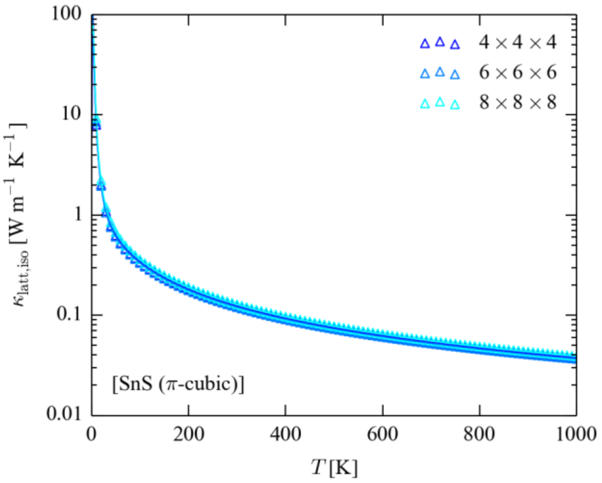

Figure S20 Isotropically-averaged lattice thermal conductivity (κ_{latt}) of π-cubic SnS computed with various lifetime-sampling **q**-point meshes.

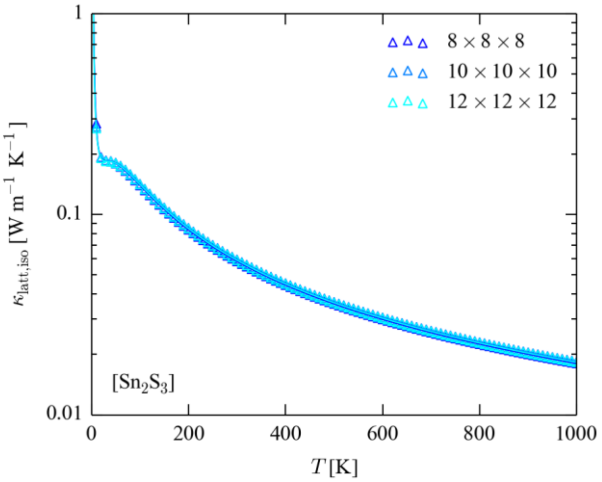

Figure S21 Isotropically-averaged lattice thermal conductivity (κ_{latt}) of Sn_2S_3 computed with various lifetime-sampling **q**-point meshes.

9. References

1. G. Kresse and J. Hafner, *Phys. Rev. B*, 1993, 47, 558(R).
2. A. Togo, F. Oba and I. Tanaka, *Phys. Rev. B*, 2008, **78**, 134106.
3. A. Togo and I. Tanaka, *Scr. Mater.*, 2015, **108**, 1.
4. A. J. Jackson, ascii-phonons, https://github.com/ajjackson/ascii-phonons.
5. J. M. Skelton, D. Tiana, S. C. Parker, A. Togo, I. Tanaka and A. Walsh, *J. Chem. Phys.*, 2015, **143**, 064710.
6. A. Togo, L. Chaput and I. Tanaka, *Phys. Rev. B*, 2015, **91**, 094306.
7. J. M. Skelton, L. A. Burton, S. C. Parker, A. Walsh, C.-E. Kim, A. Soon, J. Buckeridge, A. A. Sokol, C. R. A. Catlow, A. Togo and I. Tanaka, *Phys. Rev. Lett.*, 2016, **117**, 075502.
8. J. M. Skelton, A. J. Jackson, M. Dimitrievska, S. K. Wallace and A. Walsh, *APL Mater.*, 2015, **3**, 041102.
